# Study on interfacial shear characteristics and progressive failure model of geotextile bags

**DOI:** 10.1371/journal.pone.0321058

**Published:** 2025-06-02

**Authors:** Hui Li, Miao Shi, Jia Zhang, Liangliang Peng

**Affiliations:** 1 Institute of Urban Rail Transit, Liaoning Railway Vocational and Technical College, Jinzhou, China; 2 School of Civil Engineering, Liaoning University of Technology, Jinzhou, China; 3 Institute of Railway Engineering, Liaoning Railway Vocational and Technical College, Jinzhou, China; University of Sharjah, UNITED ARAB EMIRATES

## Abstract

Geotextile bag dams withstand the earth pressure of tailings in tailings reservoir by the shear stress on interface of geotextile bags. To improve the interfacial friction characteristics, pull-out tests were conducted on geotextile bags filled with fine tailings slurry containing various cement content, and the influences of cement content on the interfacial shear characteristics were explored. To describe the progressive failure of the interface, the theoretical analysis of interface pull-out behavior was performed. The results revealed that adding content cement into fine tailings slurry not only augmented the shear strength and residual strength ratio of the interface but also postponed interface softening. The optimal amount of cement was 3%, and the apparent friction angle which played a dominant role in interface strength was increased 17.4%, compared with no cement used. The elastic-plastic model and trilinear softening model were respectively improved to conform to the interfacial shear characteristics obtained in pull-out tests under low normal stress (< 60 kPa) and high normal stress (≥60 kPa). The interface shear stiffness of geotextile bag with the interfacial displacement within 20 mm and the dynamic compression modulus along the drawing direction were regarded as significant model parameters. The comparative analysis of shear stress-displacement curves, which were obtained from model calculating and test measuring, was carried out to verify the reliability and rationality of the model. The evolution patterns of the plastic zone, softening zone and residual zone of the interface were analyzed based on the model, and the warning values for interface failure were proposed. The development degree of interfacial plastic zone, softening zone and residual zone can be approximately calculated by quadratic polynomial of normalized pull-out force. The research results of this paper will provide reliable theoretical support for the design, construction and operation management of geotextile bag dams.

## 1 Introduction

Geotextile bags are filled with sand slurry, which after multiple fillings, dewatering, and consolidating processes, form a composite soil body enveloped in woven fabric [1]. Geotextile bag technology has been widely used by scholars domestically and internationally in projects such as dam construction [2], embankment reinforcement [3], land reclamation [4], coastline management [5,6], and sludge dewatering [7], achieving significant results and accumulating extensive construction experience. Utilizing geotextile bag technology to construct dams with fine tailings not only recycles fine tailings, alleviating resource shortages for dam construction but also reduces the volume of fine tailings stored in tailings ponds, effectively suppressing dust in the storage area and indirectly increasing the effective storage capacity. Additionally, the containment and restraint provided by the geotextile fabric improve the stability of the dam, ensuring the safety of life and property for downstream residents.

It will be significantly contributed to the development of geotextile bag dam technology to improve the interfacial strength and master the failure evolution mechanisms of geotextile bag interface [8]. Scholars both domestically and internationally have conducted extensive pull-out tests to research interfacial shear characteristics [9]. They found that many factors affected interface pull-out behavior, such as loading method [10], geotextile type [11], normal stress [12], grain composition and water con-tent [13]. However, overcoming the conflict between permeability and utilization ratio of fine tailings was also a critical problem in the construction of geotextile bag dams [14]. To improve the construction efficiency and utilization ratio of fine tailings, the solidification technology from dredging, foundation, and subgrade engineering can be referenced [15]. Li [16] and Wang [17] used low content cement, PAM, and PAC to improve dewatering progress of geotextile bags. Therefore, the interfacial shear strength of these bags, whose filling characteristics were improved, were rarely studied.

Scholars have proposed and improved models such as the ideal elastic-plastic model [18], hyperbolic model [19], trilinear model [20], quadlinear model [21], and exponential model [22] to describe the relationship between shear stress and shear dis-placement at the contact interfaces of different materials, providing valuable experience for the study of geosynthetic interfaces. Pierozan [23] proposed analytical models to establish the relationship between soil-geosynthetic interface pullout resistance and to predict pullout responses. Gao [24] divided the pullout curves into three stages as follows: rapid growth stage, development transition stage and yielding stabilization stage. Liu [25] believed that the friction characteristics of the geogrid-soil interface in pullout tests were related to the tensile modulus of the geogrid and the initial stiffness of the soil reinforcement interface. When the interface displacement was small, the interface shear stress and displacement follow a linear relationship. Huang [26] employed the hyperbolic model to examine the changes in interface shear stress along the interface length, yet overlooked the elastic characteristics of shear stress and dis-placement during the initial stage of pullout. Makkar [27] and Cheng [28] proposed interface softening models based on statistical damage theory. Many scholars have confirmed through experimental studies that the shear stress distribution at the inter-face of geosynthetics is non-uniform, and the interface failure exhibits distinct progressive characteristics [29–31]. To more accurately reflect the progressive failure at the interface in reinforced structures, some scholars have adopted progressive failure theory to describe interface models. Hong [32] used ideal elastic-plastic models to simulate the progressive failure processes of the soil nailing interface on slopes and the pile-soil interface, respectively. Zhu [33] and Chen [34] employed trilinear interface models to describe the progressive failure characteristics of fiber-reinforced soil inter-faces and grouted anchor interfaces, obtaining analytical solutions for interface shear stress and axial load at different stages. Lai [35] analyzed the relationship between interface shear stress and displacement in pullout tests of geogrid-soil interfaces, finding that after the peak shear stress, the interface shear stress decreases approximately exponentially with increasing displacement. They proposed an elastic-exponential softening model to reflect the progressive failure characteristics of the interface. However, studies on failure models for geotextile bag interfaces were exceedingly rare.

In this study, pull-out tests were performed between geotextile bags filled by fine tailings slurry mixed with low content cement. The influences of cement content on the interfacial shear characteristics were explored, and the optimal cement content was discussed. To reveal the evolution of failure at the interface, improved ideal elastic-plastic and trilinear softening models were proposed to elucidate the evolution patterns of the plastic, softening, and residual zones at the interface. The warning values for interface failure were proposed, and summarize empirical formulas relating the development of these zones to the normalized thrust at the loaded end.

## 2 Pull-out test of geotextile bag interface

### 2.1 Testing materials

**Fig 1:**
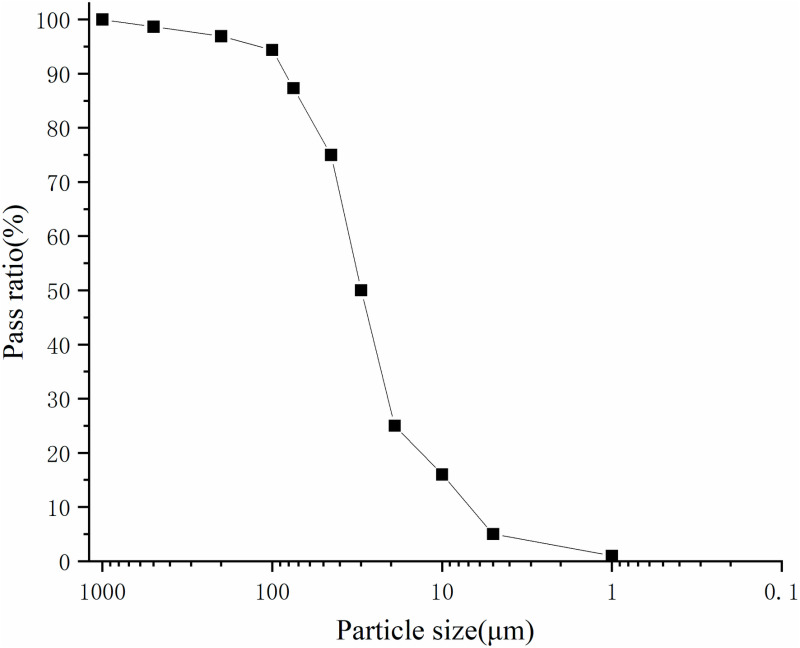
Gradation curve of fine tailings used in filling tests.

Geotextile bags with sizes of 300 × 300 mm were sewn with polypropylene woven fabrics and nylon thread whose strength exceeded 180 N. A filling port with a diameter of 45 mm and a height of 100 mm was set at the top center of geotextile bags. The filling port was tightly stitched with the bags to ensure no slurry leakage at the joints. The fine tailings used in filling tests were gold tailings, whose passing rate exceeded 80% when sieving by the 0.075mm screen, as shown in Fig 1. The filling slurry with a mass concentration of 40% was configured by these gold tailings, and was denoted as sample S0 [14]. Ordinary Portland cement (P.O42.5) was selected as curing agent. Cement accounting for 1%, 3% and 5% of the mass of gold tailings was mixed into sample S_0_, and the uniformly mixed slurries were recorded as sample S_1_, S_3_ and S_5_, respectively.

**Fig 2:**
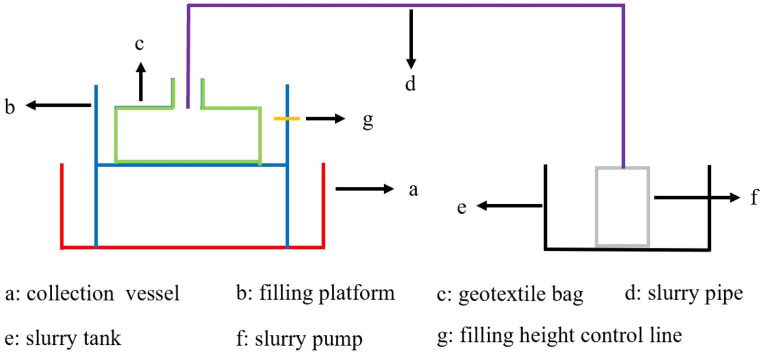
Filling tests device.

**Fig 3:**
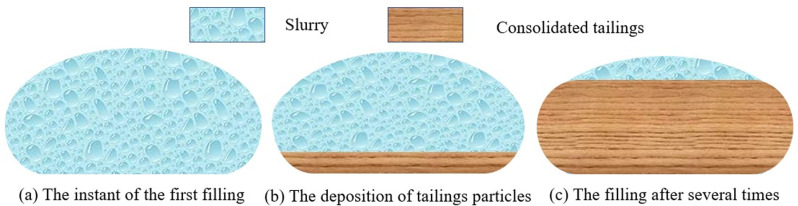
The filling and consolidating processes.

The tailings slurry filled into the geotextile bags while stirring as illustrated in Fig 2, then the filling and consolidating processes were described in Fig 3. The discharged slurry volume in the collection vessel was measured every 20 minutes. If the discharged slurry volume was less than 500 mL within 20 minutes, slurry dis-charge was considered complete, and the next filling cycle was started. Generally, the best reinforcement effect was achieved when the volume of consolidation tailings in geotextile bags reached 85%-90% of the geotextile bag capacity [36]. It was inevitable that some water was discharged even after completion of filling tests, which decreased the height of consolidated tailings inside the bags. Therefore, the optimal filling ratio in this paper was determined to be 90%, where the height of consolidated tailings in geo-textile bag first reached 7 cm. 12 geotextile bags were prepared with the same filling sample. The improvement of the interfacial friction properties between the cement-solidified geotextile bags primarily depended on the hydration reaction of cement within filling slurry. Therefore, the filled geotextile bags were consolidated for 7 days to facilitate early strength development.

### 2.2 Testing instruments

**Fig 4:**
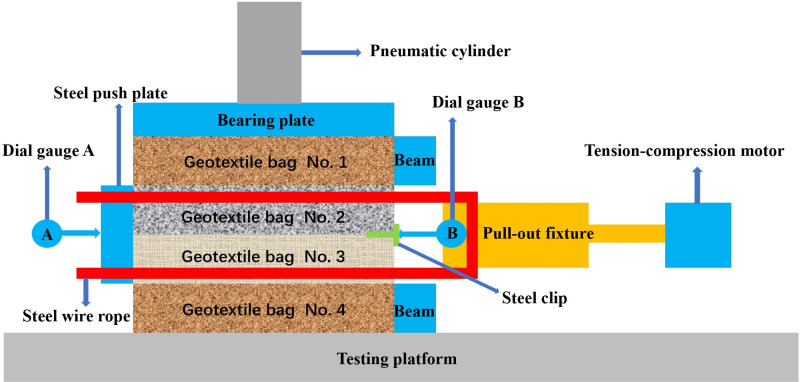
Schematic diagram of the geotextile bag interface pull-out test.

**Fig 5:**
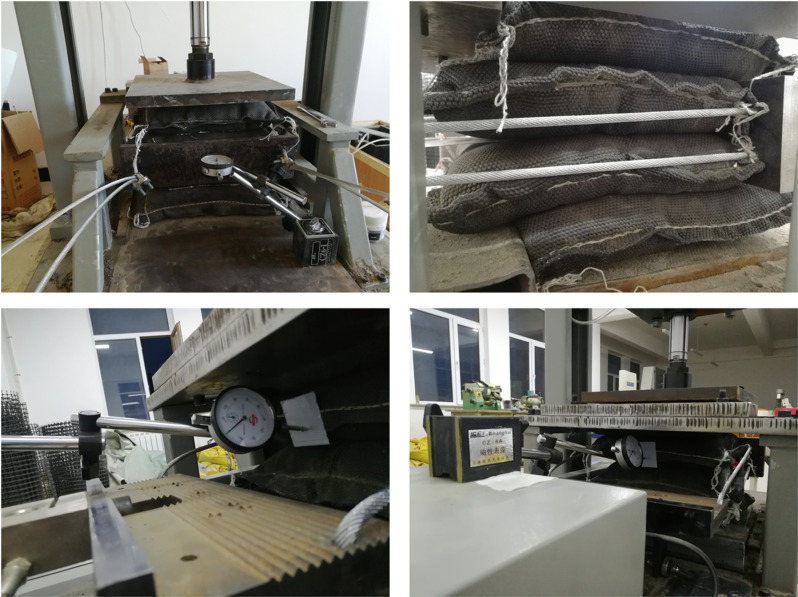
The progress of pull-out test.

The pull-out test was carried out utilizing a modified YT1200 geosynthetic pull-out testing system, as shown in Figs 4 and 5. A pneumatic cylinder equipped with pressure sensors was utilized to apply vertical loading, exerting the necessary overlying pressure via a reaction device. A bearing plate with sizes of 300 × 300× 10 mm was positioned under the pneumatic cylinder. A tension-compression motor, equipped with a controllable loading rate and a tension sensor, was utilized for horizontal loading. This motor was capable of applying a consistent loading rate within the range of 0–5 mm/min over a 100 mm distance and also measuring the pull-out force.

### 2.3 Testing design

As shown in Fig 5, four geotextile bags filled with the same sample were stacked vertically. To enhance the interface strength between geotextile bags, the weft of the woven fabric on the bottom of the bags should be aligned parallel to the pull-out direction when stacked vertically [37]. To reduce the impact of the filling ports on the interfacial friction properties, they were eliminated before bags vertically stacked. The filling port of the top bag was positioned upwards, the port of the bottom bag was directed downwards, and the ports of the two middle bags were aligned facing each other. Two fixed beams were utilized to ensure that the top and bottom bags remained stationary during pull-out test. At the free end of the bags, a steel clip was placed on the front section of the two middle bags. To simulate the vertical loads at various depths of a dam constructed with geotextile bags, a vertical loading system was utilized to apply pressures of 1.8, 3.6, 5.4, and 7.2 kN. The interface profiles of the bags were then meticulously marked. Based on these markings, the normal stress acting on the interface could be calculated. Two steel wires were threaded through the bolt holes, serving as the means to connect the pull-out fixture to a steel push plate with dimensions of 400 × 100 × 20 mm. The tensile-compression motor’s loading rate was adjusted to 2 mm/min, with the steel wires functioning to actuate the push plate. This configuration enabled the application of a precisely measurable horizontal force to the two middle bags, effectively simulating the earth pressure imposed on the dam. To eliminate the effect of steel wire deformation on the interface pull-out displacement, two dial gauges were used to measure the displacement of the push plate and clip every minute, which might be respectively considered as the displacement at the loaded end and free end of the bags. To reduce the discreteness of pull-out tests, three parallel tests were conducted for various cement content geotextile bags. If the deviation was within 10%, the average value was taken for result calculation.

## 3 Results and analysis

### 3.1 Distribution pattern of shear stress at the interface

When the push plate exerted a horizontal thrust on the two middle bags, shear stress developed at the interface to prevent their sliding as shown in Fig 5. Given the typically high length-to-width ratio of geotextile bags, the force acting on the interface can be approximated as a plane strain problem. With the free end of the geo-textile bag serving as the origin, and the positive *x-axis* directed opposite to the bag’s pull-out direction, the shear stress at position *x* along the interface was denoted as *τ*(*x*). Consequently, the force resisting sliding at the interface was formulated as:


$$T\left(x\right)=2\int_{0}^{x}\tau \left(x\right)$$ (1)

Assuming that the interface thickness and elastic modulus remained constant throughout the pull-out process, denoted as *h* and *E*, respectively. Consequently, the stress and strain at position *x* along the interface can be expressed as follows:


$$\sigma \left(x\right)=\frac{\left[2\int_{0}^{x}\tau \left(x\right)dx\right]}{h}$$ (2)

$$\varepsilon \left(x\right)=\left[2\int_{0}^{x}\left(x\right)\tau dx\right]/Eh$$ (3)

The displacement observed at the interface, located at position *x*, was as follows:


$$L\left(x\right)=L_{A}+\int_{0}^{x}\varepsilon \left(x\right)dx=L_{A}+\int_{0}^{x}\left[2\int_{0}^{x}\tau \left(x\right)dx\right]/Ehdx$$ (4)

where *L*_A_ represented the sliding displacement of the geotextile bag.

If the shear stress was uniformly distributed across the interface, then the dis-placement at the loaded end would be expressed as follows:


$$L_{\text{B}}=L_{\text{A}}+\frac{D^{2}\tau }{Eh}$$ (5)

where *D* represented the interface length measured along the pull-out direction, and the displacement difference between the loaded end and the free end remained constant.

**Fig 6:**
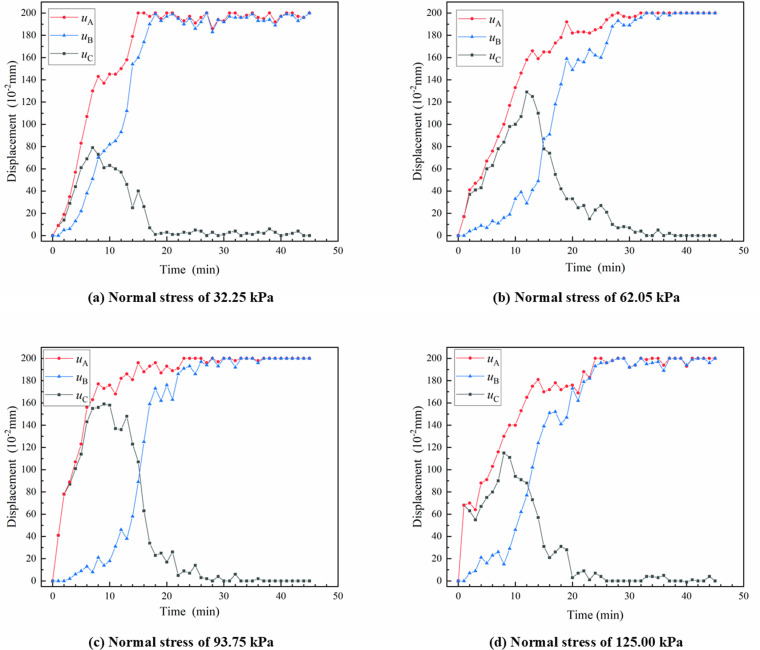
Time-history curves of the displacement for the force end and free end of the geotextile bags.

During the pull-out test, the displacements of the push plate and the clip were used as proxies for the displacements at the loaded end and the free end, respectively. Using the geotextile bag interface with a cement content of 3% and consolidated for 7 days as an illustrative example, the distribution pattern of shear stress at the interface was examined. Fig 6 displayed the time history curves of the push plate displacement (*l*_*A*_), clip displacement (*l*_*B*_), and their displacement difference (*l*_*C*_), which were measured at one-minute intervals under various levels of normal stress.

As depicted in Fig 6, both *l*_*A*_ and *l*_*B*_ exhibited an increasing trend over time, ultimately stabilizing and synchronizing with the loading rate of the pull-out fixture. The progression of *l*_*B*_ lagged behind *l*_*A*_, with the degree of lag intensifying as the normal stress increased. Initially, *l*_*C*_ first increased, followed by a decrease, and eventually stabilized at zero. The time required for *l*_*C*_ to converge to zero increased with the rise in normal stress. Under the same normal stress, the stabilization times for both *l*_*B*_ and *l*_*C*_ were approximately equivalent. Prior to the stabilization of *l*_*C*_, the displacement difference between the loaded and free ends initially augmented and subsequently di-minished over time. During this period, the shear stress distribution at the geotextile bag interface was uneven. This phenomenon might be attributed to the gradual trans-fer of shear stress at the interface, progressing from the loaded end towards the free end. A higher normal stress corresponded to greater resistance to this transfer, resulting in a slower transfer process. Therefore, before the stabilization of *l*_*C*_, the displacement at the loaded end was primarily attributed to compression deformation, with the magnitude of this deformation being equivalent to the accumulated value of *l*_*C*_. After *l*_*C*_ stabilized, the displacement rates at both the loaded and free ends increased in tandem with the pull-out speed. Under the influence of horizontal thrust, the geotextile bag underwent overall translation, and the shear stress at the geotextile bag interface was uniformly distributed. This finding provided some evidence that progressive failure characteristics were occurred at the interface of geotextile bags.

### 3.2 Influence of cement content on the shear stress-pullout displacement curve

**Fig 7:**
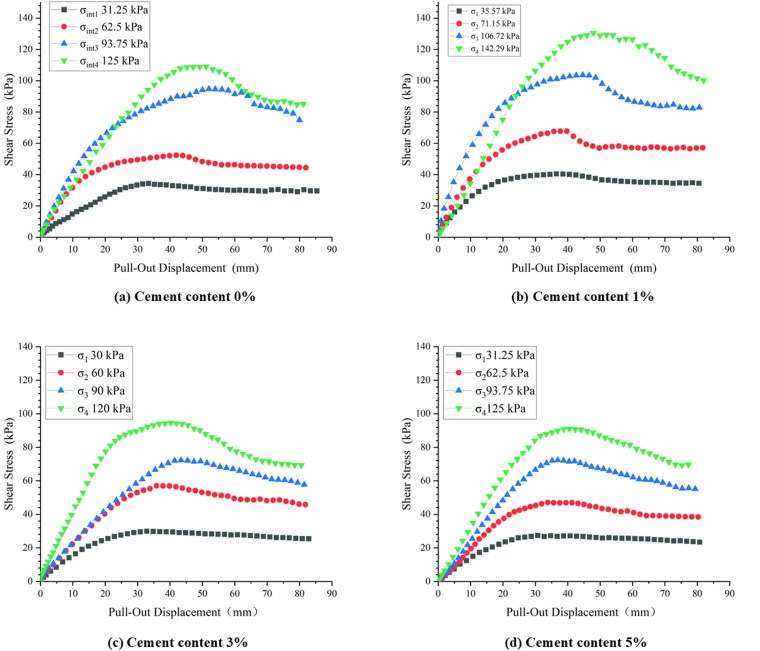
Shear stress and pull-out displacement curves at different cement contents.

To examine the effect of cement content on the friction characteristics at the interface, it was postulated that the shear stress was evenly distributed throughout the pull-out test, and the displacement of the push plate was taken as the measure of pull-out displacement at the geotextile bag interface. Fig 7 displayed the shear stress-pullout displacement curves obtained at various cement.

**Fig 8:**
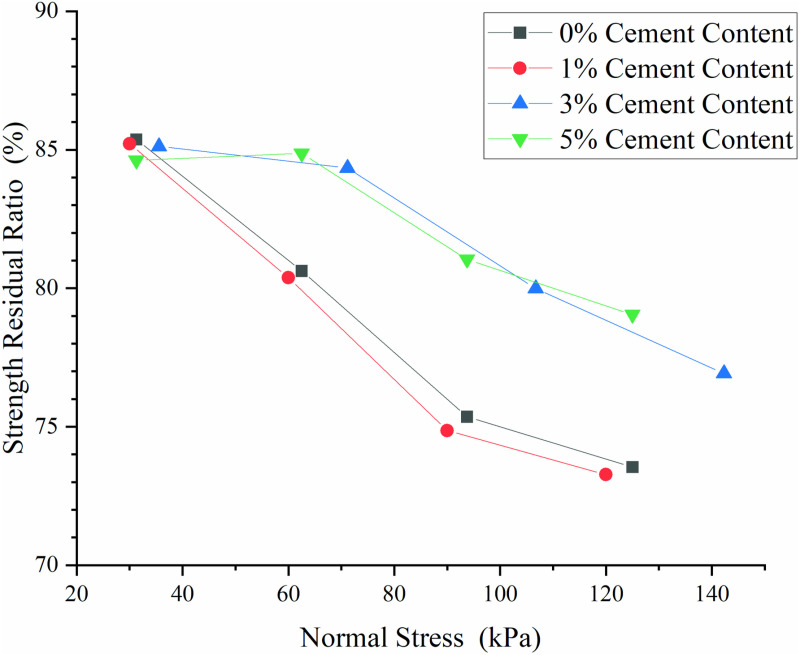
Residual Strength Rate of Interfaces at Various Cement Contents.

As illustrated in Fig 7, the variation in shear stress with pull-out displacement exhibited a generally consistent pattern across all interfaces. Upon reaching the peak shear stress, the interfaces experienced varying degrees of softening, gradually stabilizing thereafter. The shear stress corresponding to a pull-out displacement of 80 mm was designated as the large-displacement strength, and the ratio of this value to the peak shear stress was termed the residual strength ratio. This ratio served as a quantitative metric to analyze the influence of cement content on the shear stress-pullout displacement relationship, as illustrated in Fig 8. The residual strength ratio exhibited a range of 70% to 85% and demonstrated a negative correlation with normal stress. When the normal stress was less than 40.00 kPa, the cement content had a negligible impact on the residual strength ratio. In the absence of cement or with the addition of 1% cement, the residual strength ratio decreased rapidly with increasing normal stress, with minimal difference observed between these two conditions at the same normal stress. Conversely, when the cement content was elevated to 3% and 5%, the residual strength ratio began to decline significantly only at normal stresses exceeding 60.00 kPa, with a slower rate of decrease compared to lower cement contents. This finding suggested that a cement content of either 3% or 5% not only augmented the residual strength ratio but also postponed interface softening, albeit with minimal difference between these two cement levels.

### 3.3 Influence of cement content on the peak displacement at the interface

**Fig 9:**
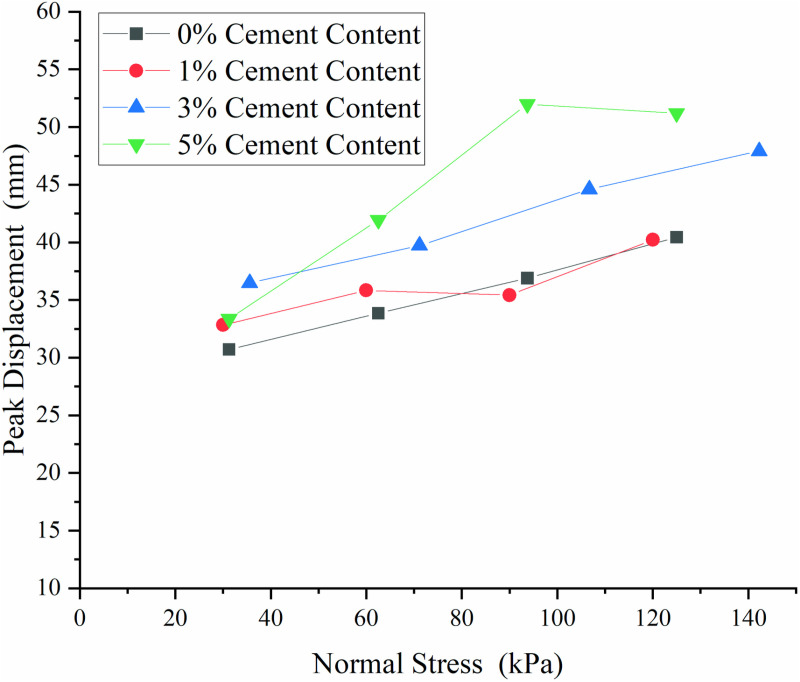
Peak displacement of interfaces at different cement content.

The peak displacement corresponding to the peak shear stress at various normal stresses was obtained from Fig 7, and its correlation with normal stress was depicted in Fig 9. As normal stress increased, the transmission of pull-out displacement and shear stress became more difficult, leading to an increase in peak displacement. This observation was in agreement with the data presented in Fig 6. When 1% cement was incorporated, a slight increase in peak displacement was observed for normal stress below 80.00 kPa. As the normal stress rose, the influence of cement con-tent on peak displacement became negligible. In comparison to S_0_, the peak displacement of S_3_ witnessed an approximate 20% enhancement. Notably, when normal stress surpassed 80.00 kPa, the peak displacement of S5 increased by a range of 25% to 40%. This suggested that as normal stress increased, the impact of cement content on peak displacement becomes more pronounced.

### 3.4 Influence of cement content on the interface shear strength

**Fig 10:**
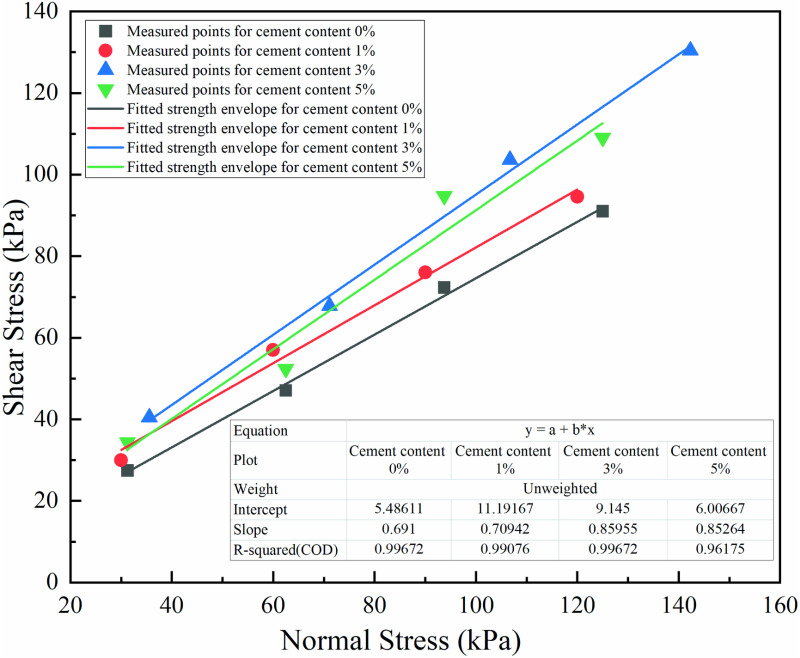
Failure envelopes of interfaces at different cement contents.

The interface shear strength of geotextile bags with different cement contents was fitted, and the strength envelope was plotted as shown in Fig 10. Notably, the correlation coefficients of the fitted interface strength envelopes all exceeded 0.95.

The interface shear strength was governed by both the apparent friction angle and apparent cohesion, with the apparent friction angle serving as the primary determinant, while the apparent cohesion fluctuating within a range of 5–10 kPa. Specifically, the S_0_ interface exhibited an apparent friction angle of 34.64°, whereas the S_1_, S_3_, and S_5_ interfaces demonstrated angles of 35.35°, 40.68°, and 40.45°, respectively, marking increases of 2.05%, 17.44%, and 16.77%. This indicated that, at a cement content of 1%, the enhancement in interface strength was primarily attributed to an increase in ap-parent cohesion, albeit with minimal impact on overall interface strength. Conversely, for cement contents of 3% and 5%, the improvement was predominantly driven by an increase in the apparent friction angle, with the enhancement becoming more significant as normal stress increased. As depicted in Fig 10, the interface strength lines for cement contents of 3% and 5% were nearly parallel, implying that their contributions to augmenting the apparent friction angle were virtually indistinguishable.

Figs 7 to 10 shown that adding 1% cement had a negligible effect on improving the frictional properties of the geotextile bag interface. When the cement content was increased to 3%–5%, the interface frictional properties were significantly enhanced, though the difference between these two levels was minimal. To save construction cost, it was recommended to set the optimal cement content for improving the frictional properties of the geotextile bag interface at 3%.

## 4 Progressive failure model of the geotextile bag interface

### 4.1 Control equation of the geotextile bag interface

**Fig 11:**
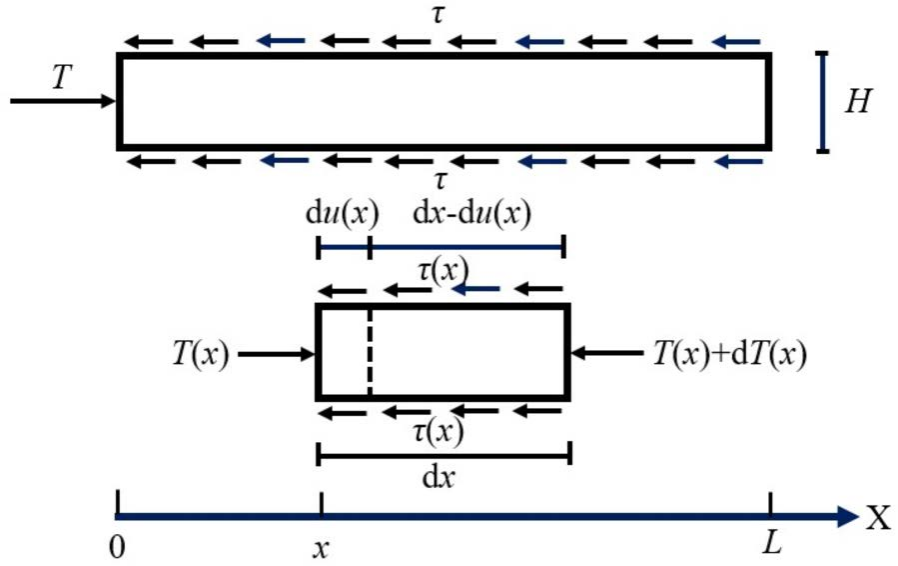
Force analysis of micro-unit body of geotextile tube.

In the pull-out test, the two middle geotextile bags, which were pulled out collectively, were treated as a singular unit possessing a unit weight of γ, thickness H, length L, and width W. When these bags resisted the earth pressure T exerted by the tailings stored in the reservoir, shear stress τ was observed at the interface of the bags. Along the direction of pull-out, a microelement of length d*x*, located at a distance *x* from the loaded end, was chosen for detailed force analysis, as illustrated in Fig 11.

Before the two middle bags sliding, the shear stress was unevenly distributed on the interface, as mentioned in 2.1. Given the minimal length of the microelement d*x*, it was postulated that the shear stress was uniformly distributed across d*x* when con-structing the mechanical model of the interface. *u(x)* represented the displacement of the microelement dx along the interface under the action of the thrust *T(x)*. If the microelement was in equilibrium, then the following static equilibrium equation must exist:


$$T\left(x\right)-2W\left[\text{d}x-\varepsilon \left(x\right)dx\right]\tau \left(x\right)-\left[T\left(x\right)+dT\left(x\right)\right]=0$$ (6)

In the Equation (6):

*T(x)* represented the thrust acting on the microelement d*x*, measured in kN;

*τ(x)* denoted the interface shear stress on the microelement d*x*, measured in kPa;

$-\varepsilon \left(x\right)dx$ indicated the interface displacement caused by the thrust *T(x)* on the microelement d*x*, measured in meters;

*W* stood for the width of the geotextile bag, measured in meters.

Since the value of in pull-out test was extremely small and could even be neglected, equation (6) can be rearranged and reformulated as follows:


$$\frac{dT\left(x\right)}{\text{d}x}=-2W\tau \left(x\right)$$ (7)

Under the action of thrust *T(x)*, the strain at the microelement dx satisfied the following relationship:


$$E\left(x\right)=\frac{\sigma \left(x\right)}{\text{ε}\left(x\right)}$$ (8)

In the Equation (8):

*E(x)* represented the compression modulus of the microelement d*x*;

$\sigma \left(x\right)$ denoted the normal stress acting on the cross-section of the microelement d*x*.

Assuming the cross-sectional area of the two middle bags remained constant as during the pull-out test, then $\sigma \left(x\right)=\frac{T\left(x\right)}{HW}$. Based on the calculation, the normal stress acting $\sigma \left(x\right)$ was much smaller than the compressive strength of the geotextile bags, meaning that the compression deformation of the two middle bags must be in the elastic phase. Therefore, the compression modulus *E(x)* at any position on the geotextile bags can be replaced with the compression modulus *E*.

Thus, the thrust *T(x)* can be expressed as:


$$T\left(x\right)=-E\cdot H\cdot W\cdot \frac{du\left(x\right)}{\text{d}x}$$ (9)

Substituting equation (9) into equation (7), then:


$$EH\frac{d^{2}u\left(x\right)}{dx^{2}}=2\tau \left(x\right)$$ (10)

Throughout the entire pull-out test, the shear stress and displacement at any position on the interface satisfied equation (10), which can be used as the control equation of the geotextile bag interface.

### 4.2 Derivation of the interfacial progressive failure model

**Fig 12:**
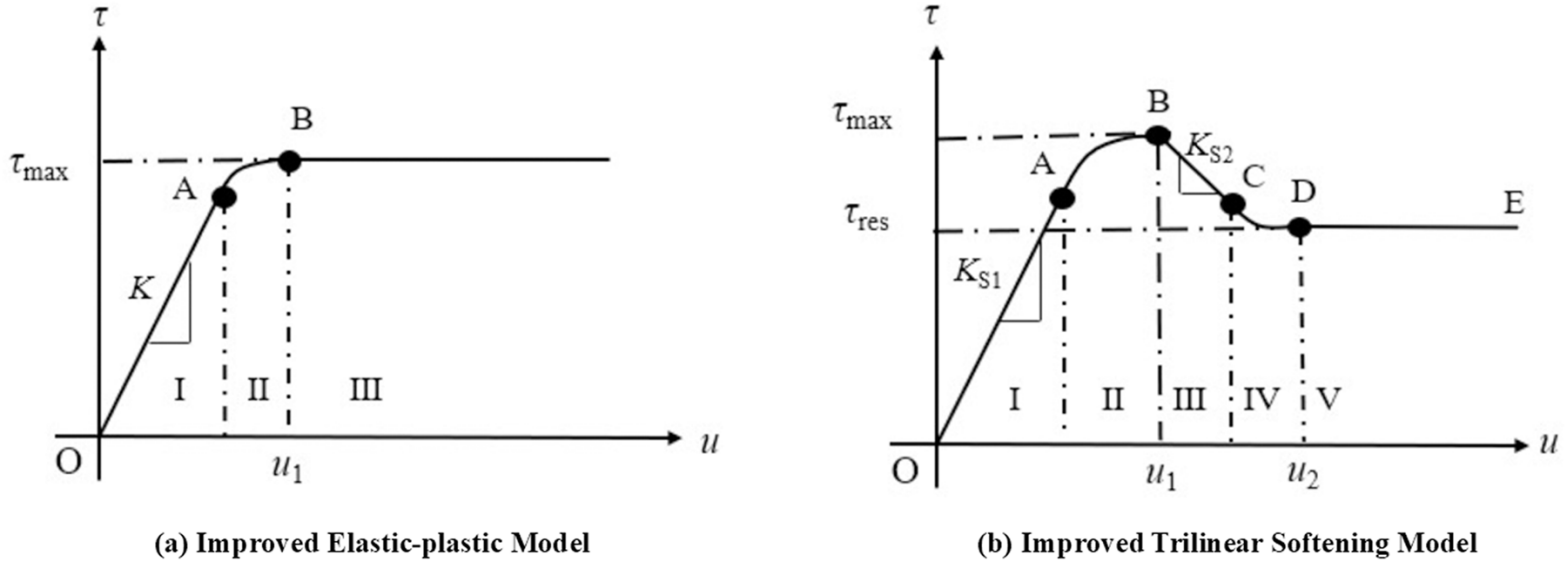
The interfacial constitutive model between geotextile bags after improving.

To illustrate the pull-out behavior shown in Fig 7, the models depicting progressive failure under conditions of low normal stress (<60 kPa) and high normal stress (≥60 kPa) can be approximately represented by Fig 12(a) and (b).

#### 4.2.1 Interfacial progressive failure model under low normal stress.

The improved ideal elastic-plastic model shown in Fig 12(a) can be categorized into three stages: a fully elastic, an elastic-plastic transition, and a fully plastic.

1. Fully elastic stage

At this stage, a linear relationship was observed between the shear stress and displacement on the interface. When this relationship was substituted into Equation (10), we derived the following:


$$\frac{d^{2}T}{dx^{2}}-\alpha^{2}T=0$$ (11)

Where α was the interface characteristic influence factor, denoted as $\alpha =\sqrt{\frac{2K}{EH}}$.

In the pull-out test, the two middle bags obtained compression deformation in the direction of pull-out as a consequence of the applied thrust. However, the com-pression deformation of the bags was constrained by the normal stress acting on them, with increased normal stress resulting in greater constraint. Therefore, the compression deformation in the pull-out test was completed under confined conditions. If the interface characteristic influence factor was calculated based on the elastic modulus derived from the compression strength test of geotextile bags, it will impart notable inaccuracies into the computational results. Therefore, the displacement occurring at the free end should be considered. The difference in displacement between the loaded and the free ends should be employed as the measure of compressive deformation, which in turn should be utilized for computing the strain in the geotextile bag.

Let the thrust at the loaded end to be T_0_. Based on the boundary conditions at interface, solving Equation (11), the thrust *T*_e_(*x*), interface shear stress *τ*_e_(*x*), and interface displacement *u*_e_(*x*) at a distance *x* from the loaded end were obtained as follows:


$$T_{e}\left(x\right)=\frac{T_{0}}{1-{\text{e}}^{-2\alpha L}}\cdot \left({\text{e}}^{-\alpha x}-{\text{e}}^{-2\alpha L+\alpha x}\right)$$ (12)

$$\tau_{e}\left(x\right)=\frac{\alpha }{2}\cdot \frac{T_{0}}{W}\cdot \frac{{\text{e}}^{-\alpha x}+{\text{e}}^{-2\alpha L+\alpha x}}{1-{\text{e}}^{-2\alpha L}}$$ (13)

$$u_{e}\left(x\right)=\frac{T_{0}}{W}\cdot \frac{{\text{e}}^{-\alpha x}+{\text{e}}^{-2\alpha L+\alpha x}}{E\cdot H\cdot \alpha \cdot \left(1-{\text{e}}^{-2\alpha L}\right)}$$ (14)

It was evident that *T*_e_(*x*), *τ*_e_(*x*) and *u*_e_(*x*) were all related to the interface characteristic influence factor, and *τ*_e_(*x*) decayed exponentially from the loaded end to the free end. In Equation (14), setting *x* = 0, the displacement at the loaded end can be calculated using Equation (15), which shown a linear relationship with the thrust at the loaded end.


$$u_{e}\left(0\right)=\frac{T_{0}}{W}\cdot \frac{1}{E\cdot H\cdot \alpha }\cdot \frac{1+{\text{e}}^{-2\alpha L}}{\alpha \left(1-{\text{e}}^{-2\alpha L}\right)}$$ (15)

Substituting *x* = 0 and *τ*_e_(0) = *τ*_max_ into Equation (13), the critical thrust, donated as *T*_e-p_, at the loaded end for the transition from the fully elastic stage to the elastic-plastic transition stage can be obtained as follows:


$$T_{e-p}\left(0\right)=2\cdot W\cdot \tau_{max}\cdot \frac{1-{\text{e}}^{-2\alpha L}}{\alpha \left(1-{\text{e}}^{-2\alpha L}\right)}$$ (16)

Thus, the range of thrust at the loaded end when the interface was in the fully elastic stage was determined as $T_{0}\in \left[0,T_{e-p}\left(0\right)\right]$.

2. Elastic-Plastic transition stage

As the thrust at the loaded end increased, the plastic zone of the interface ex-tended from the loaded end toward the free end. Assume the interface length of the plastic zone was *l*_*p*_ (0≤*l*_*p*_≤L). When 0≤*x*≤*l*_*p*_, the interface was in the plastic stage with the shear stress constant at *τ*_max_; when *l*_*p*_≤*x*≤L, the interface remained in the elastic stage.

Within the elastic zone (*l*_*p*_≤*x*≤L), the internal thrust of the two middle bags *T*_e_*’* (*x*), the shear stress at the interface *τ*_e_*’* (*x*) and the displacement *u*_e_*’* (*x*) were similar to those in the fully elastic stage. The thrust at the loaded end during the elastic-plastic transition stage was as follows:


$$T_{p}\left(0\right)=2W\tau_{max}l_{p}+2W\tau_{max}\cdot \frac{1}{\alpha }\cdot \frac{1-{\text{e}}^{-2\alpha \left(L-l_{p}\right)}}{1+{\text{e}}^{-2\alpha \left(L-l_{p}\right)}}$$ (17)

If the thrust at the loaded end during the elastic-plastic transition phase was known, the plastic zone range *l*_*p*_ can be back-calculated using Equation (17). By setting in Equation (17), the thrust at the loaded end when the entire geotextile bag interface entered the plastic zone, donated as $T_{\text{p-L}}\left(0\right)$, can be determined as follows:


$$T_{p-L}\left(0\right)=2\cdot W\cdot L\cdot \tau_{max}$$ (18)

From Equation (18), the range of thrust at the loaded end during the elastic-plastic transition phase can be determined as $T_{0}\in \left[0,T_{e-p}\left(0\right)\right]$. The interface displacement in the plastic zone (0≤*x*≤*l*_*p*_) was as follows:


$$u_{p}\left(x\right)=\frac{\tau_{max}}{EH}\left(x^{2}-l_{p}^{2}\right)-\frac{1}{EH}\cdot \frac{T_{p}\left(0\right)}{W}\cdot \left(x-l_{p}\right)+\frac{\tau_{max}}{K}$$ (19)

In this equation, *K* was the linear coefficient between interface stress and inter-face displacement.

Substituting *x* = 0 into Equation (19), the relationship between the thrust at the loaded end and its displacement during the elastic-plastic transition phase can be ex-pressed as follows:


$$T_{p}\left(0\right)=W\cdot l_{p}\cdot \tau_{max}+\frac{W\cdot E\cdot H}{l_{p}}\cdot \left[u_{p}\left(0\right)-\frac{\tau_{max}}{K}\right]$$ (20)

It can be seen that the thrust at the loaded end and its displacement exhibited a nonlinear relationship.

In Equation (19), setting *x* = 0, *l*_p_ = *L*, and combining it with Equation (18), the loaded end interface displacement at the critical point between the elastic-plastic transition phase and the fully plastic phase can be determined as follows:


$$u_{p-L}\left(0\right)=\frac{\tau_{max}}{K}+\frac{\tau_{max}}{EH}L^{2}$$ (21)

3. Fully plastic stage

When the thrust at the loaded end remained constant, the two middle bags will slide along the interface as a whole. At a certain moment in the fully plastic stage, the displacement increment at any position on the interface was the same and can be rep-resented as Δu=t⋅v, where *t* was the time duration during which the interface was in the fully plastic stage, and v was the pull-out rate, Δu represented the total displacement due to bags sliding. Therefore, in the fully plastic stage, the interface displacement at any position can be described as:


up'x=up−Lx+Δu=τmaxEHx2−2LτmaxEHx+u (22)

In the equation, u represented the displacement at the loaded end during the fully plastic stage, $u=u_{p-L}\left(0\right)+\Delta u$.

#### 4.2.2 Interfacial progressive failure model under high normal stress.

As shown in Fig 12(b), under high normal stress, the improved trilinear softening model was divided into five stages: a fully elastic, an elastic-softening transition, a fully softened, a softening-residual transition, and a fully residual.

1. Fully elastic stage

The thrust, interface shear stress, and interface displacement during the fully elastic stage can be characterized as follows:


$$T_{e}\left(x\right)=\frac{T_{0}}{1-{\text{e}}^{-2\alpha^{\prime }L}}\cdot \left({\text{e}}^{-\alpha^{\prime }x}-{\text{e}}^{-2\alpha^{\prime }L+\alpha^{\prime }x}\right)$$ (23)

$$\tau_{e}\left(x\right)=\frac{\alpha^{\prime }}{2}\cdot \frac{T_{0}}{W}\cdot \frac{{\text{e}}^{-\alpha^{\prime }x}+{\text{e}}^{-2\alpha^{\prime }L+\alpha^{\prime }x}}{1-{\text{e}}^{-2\alpha^{\prime }L}}$$ (24)

$$u_{e}\left(x\right)=\frac{\alpha^{\prime }}{2K_{S1}}\cdot \frac{T_{0}}{W}\cdot \frac{{\text{e}}^{-\alpha^{\prime }x}+{\text{e}}^{-2\alpha^{\prime }L+\alpha^{\prime }x}}{1-{\text{e}}^{-2\alpha^{\prime }L}}$$ (25)

Where $\alpha^{\prime }=\sqrt{\frac{2K_{S1}}{EH}}$ was the pre-peak interface characteristic influence factor; KS1 was the pre-peak interface shear stiffness.

When τ_e_(0) = τ_max_, the loaded end first finished the fully elastic stage and will enter the softening stage. From Equation (24), the critical thrust between the fully elastic stage and the elastic-softening transition stage was:


Te−L'0=2⋅W⋅τmaxα′⋅1−e−2α′L1+e−2α′L (26)

Then, the range of loaded end thrust when the interface was in the fully elastic stage was determined as T0∈0,Te−L'0.

2. Elastic-Softening transition phase

From the initial softening at the loaded end of the interface until the softening ex-tended to the free end, the geotextile bag interface was in the elastic-softening transition phase. If the length of the softening zone at the geotextile bag interface was donated as ls (where 0≤*l*_*s*_≤L), then softening occurred at the interface within the range 0≤*x*≤*l*_*s*_. For the range *l*_*s*_≤*x*≤L, the interface remained in the elastic phase.

When 0≤*x*≤*l*_*s*_, substituting the relationship between the shear stress and dis-placement into the interface control equation, we can obtain as follows:


$$\frac{d^{2}T\left(x\right)}{dx^{2}}+\beta^{2}T\left(x\right)=0$$ (27)

Where *u*_1_ was the post-peak interface characteristic influence factor; *K*_S2_ was the post-peak interface shear stiffness. According to the boundary conditions of the softening zone, the thrust, interface shear stress, and interface displacement within the softening zone （0≤*x*≤*l*_*s*_） can be determined as follows:


Ts'x=2⋅W⋅τmaxcosβls−xα′⋅1−e−2α′L−ls1+e−2α′L−ls+sinβls−xβ (28)

$$\tau_{s}\left(x\right)=\tau_{max}\left\{cos\left[\beta \left(l_{s}-x\right)\right]-\frac{\beta sin\left[\beta \left(l_{s}-x\right)\right]}{\alpha^{\prime }}\cdot \frac{1-{\text{e}}^{-2\alpha^{\prime }\left(L-l_{s}\right)}}{1+{\text{e}}^{-2\alpha^{\prime }\left(L-l_{s}\right)}}\right\}$$ (29)

$$u_{s}\left(x\right)=\frac{\tau_{max}}{K_{s2}}\left\{cos\left[\beta \left(l_{s}-x\right)\right]-\frac{\beta sin\left[\beta \left(l_{s}-x\right)\right]}{\alpha^{\prime }}\cdot \frac{1-{\text{e}}^{-2\alpha^{\prime }\left(L-l_{s}\right)}}{1+{\text{e}}^{-2\alpha^{\prime }\left(L-l_{s}\right)}}\right\}-\frac{\tau_{max}}{K_{s2}}+u_{1}$$ (30)

Where *u*_1_ represented the interface displacement when the interface shear stress just reached the interface strength. When the softening zone reached the free end, that was *l*_*s*_ = L, it signaled the impending transition into the fully softened phase. By substituting *x* = 0 and *l*_*s*_ = L into Equation (28), the critical thrust at the loaded end for both the elastic-softening transition phase and the fully softened phase can be determined as follows:


$$T_{s}L\left(0\right)=2\cdot W\cdot \tau_{max}\cdot \frac{sin\left(\beta L\right)}{\beta }$$ (31)

Then, the range of thrust at the loaded end when the interface was in the elastic-softening transition phase was determined as T0∈\Te−L'0,Ts'0.

3. Fully softened stage

According to the boundary condition equations, the thrust, interface shear stress, interface displacement, and the displacement at the loaded end can be calculated as follows:


$$T_{s-L}\left(x\right)=T_{s-L}\left(0\right)\left[cos\left(\beta x\right)-cot\left(\beta L\right)sin\left(\beta x\right)\right]$$ (32)

$$\tau_{s-L}\left(x\right)=\frac{\beta T_{s-L}\left(0\right)}{2W}\cdot \left[sin\left(\beta x\right)+cot\left(\beta L\right)\cdot cos\left(\beta x\right)\right]$$ (33)

$$u_{s-L}\left(x\right)=\frac{\beta T_{s-L}\left(0\right)}{2\cdot W\cdot K}\cdot \left[sin\left(\beta x\right)+cot\left(\beta L\right)\cdot cos\left(\beta x\right)\right]-\frac{\tau_{max}}{K_{s2}}+u_{1}$$ (34)

$$u_{s-L}\left(0\right)=\frac{\beta T_{s-L}\left(0\right)}{2\cdot W\cdot K_{s2}}\cdot cot\left(\beta L\right)-\frac{\tau_{max}}{K_{s2}}+u_{1}$$ (35)

In Fig 12(b), *u*_2_ represented the interface displacement when the interface shear stress just reached the residual strength. When *u*_S-L_(0) = *u*_2_, the interface at the loaded end was precisely in the residual phase, marking the interface entered the softening-residual transition phase. Therefore, the critical thrust between at the loaded end between the fully softened phase and the softening-residual transition phase was as follows:


$$T_{s-r}\left(0\right)=\frac{2\cdot W\cdot tan\left(\beta L\right)\cdot \tau_{r}}{\beta }$$ (36)

Then, the range of thrust at the loaded end when the interface was in the fully elastic stage was determined as T0∈Ts'0,Ts−r'0.

4. Softening-Residual transition phase

The residual zone extended from 0 to *l*_*r*_ (0≤*l*_*r*_≤L), within which the interface shear stress was constantly *τ*_res_. Combining the boundary conditions of the residual zone, the thrust and displacement in the residual zone can be determined as follows:


$$T_{r}\left(x\right)=2\cdot W\cdot \tau_{res}\cdot \left\{l_{r}-x+\frac{tan\left[\beta \left(L-l_{r}\right)\right]}{\beta }\right\}$$ (37)

$$u_{r}\left(x\right)=\frac{\tau_{res}}{EH}x^{2}-\frac{2\tau_{res}}{EH}\left\{l_{r}+\frac{tan\left[\beta \left(L-l_{r}\right)\right]}{\beta }\right\}\cdot x+u_{2}+\frac{\tau_{res}}{EH}l_{r}2+\frac{2\tau_{res}}{EH}\cdot \frac{tan\left[\beta \left(L-l_{r}\right)\right]}{\beta }l_{r}$$ (38)

By substituting *x* = 0 and *l*_*r*_ = L into Equations (37) and (38), the thrust and dis-placement at the loaded end can be determined when the entire interface just entered the residual stage:


$$T_{r}L\left(0\right)=2\cdot W\cdot \tau_{res}\cdot L$$ (39)

$$u_{r}L\left(0\right)=u_{r}+\frac{L^{2}}{EH}\cdot \tau_{res}$$ (40)

Then, the range of thrust at the loaded end when the interface was in the fully softened stage was determined as T0∈Ts−r'0,TrL0.

5. Fully residual stage

In this stage, the shear stress at any point on the interface was equal to the residual strength *τ*_res_. However, the interface displacement continued to increase. In Equation (38), by setting *l*_*r*_ = L, when the interface first entered the residual stage, the inter-face displacement at any point can be calculated as follows:


$$u_{r}L\left(x\right)=\frac{\tau_{res}}{EH}\cdot \left(x^{2}+L^{2}\right)-\frac{2\tau_{res}}{EH}\cdot Lx+u_{2}$$ (41)

Subsequently, as time progresses, the two middle bags will slide along the inter-face as a whole, with an incremental displacement of Δu=t⋅v at each position on the interface, where *t* was the time since entering the residual stage, and *v* was the pull-out rate. Therefore, the interface displacement at any point and at any moment during the residual stage was as follows:


$$u_{r-L}\left(x\right)=u_{r}L\left(x\right)+\Delta u=\frac{\tau_{res}}{EH}\cdot \left(x^{2}+L^{2}\right)-\frac{2\tau_{res}}{EH}\cdot Lx+u$$ (42)

Where *u* represented the interface displacement at the loaded end during the residual stage.

### 4.3 Validation of the progressive failure model for the geotextile bag interface

Based on the pull-out test data in this study, taking geotextile bags with 3% cement content and 7 days consolidation as an example, the proposed progressive failure model was validated. The pre-peak interface shear stiffness *K*_S1_ indicated the rate of increase in interface shear stress as a function of interface displacement, and its value affected the pre-peak interface characteristic influence factor *α*^′^, thereby impacting the reliability and applicability of the model. Therefore, during the validation process, three different attempts were conducted to determine the value of *K*_S1_.

**Fig 13:**
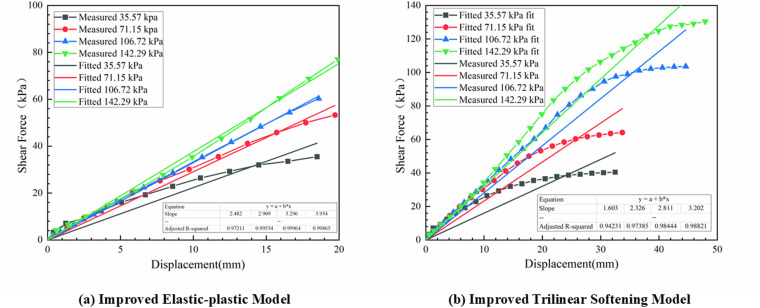
Initial shear stiffness of interface.

1. When the interface displacement was within 20 mm, the interface shear stress and displacement essentially satisfied a linear relationship. By linearly fitting the interface shear stress and displacement within this range, the slope of the fitted line was considered as the pre-peak interface shear stiffness *K*_S1_, as shown in Fig 13(a), and the corresponding model was donated as Model 1.
2. In Fig 7, all points before the peak shear stress were linearly fitted, and the slope of the fitted line was used as *K*_S1_, as shown in Fig 13(b). This corresponding model was designated as Model 2.
3. The interface shear stiffness was calculated utilizing the peak shear stress and its corresponding interface displacement, and this corresponding model was named as Model 3.

**Fig 14:**
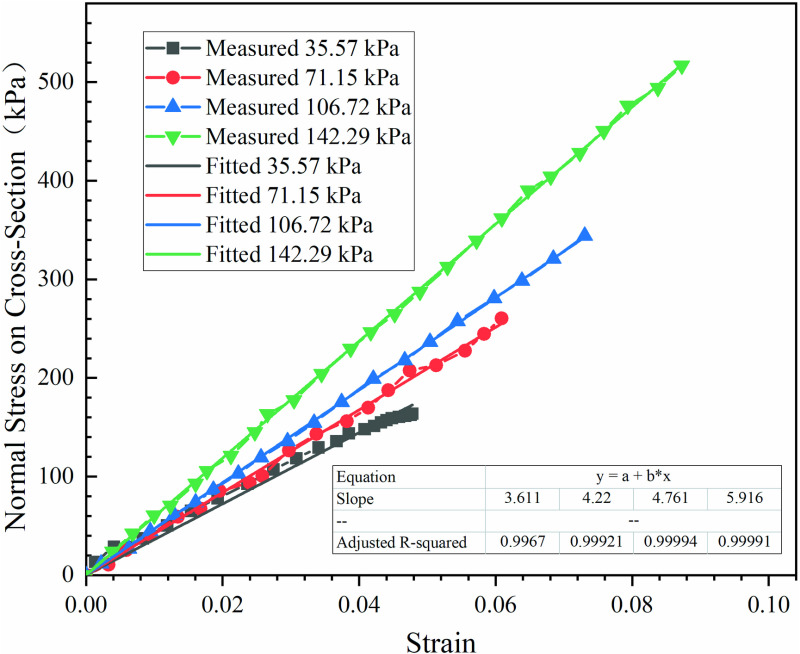
Dynamic compression modulus of geotextile tube along the drawing direction.

Assuming that the cross-sectional dimensions of the two middle bags was constant during the pull-out test. Based on the thrust at the loaded end recorded by the pull-out test data acquisition system, the normal stress can be calculated, which acted on the cross-section of the bags in the pull-out direction. Utilizing two dial gauges which maintained vertical contact with the push plate and the clip, the displacement at the loaded and free ends of the bags were respectively measured, and the strain was obtained throughout their difference. As depicted in Fig 14, the relationship be-tween the normal stress and strain on the cross-section of the bags was presented, and the compression modulus along the pull-out direction was also determined through linear fitting of these data points. It was evident that the compression modulus varied with the normal stresses. Therefore, the dynamic compression modulus should be used when validating and predicting the progressive failure of interface.

**Fig 15:**
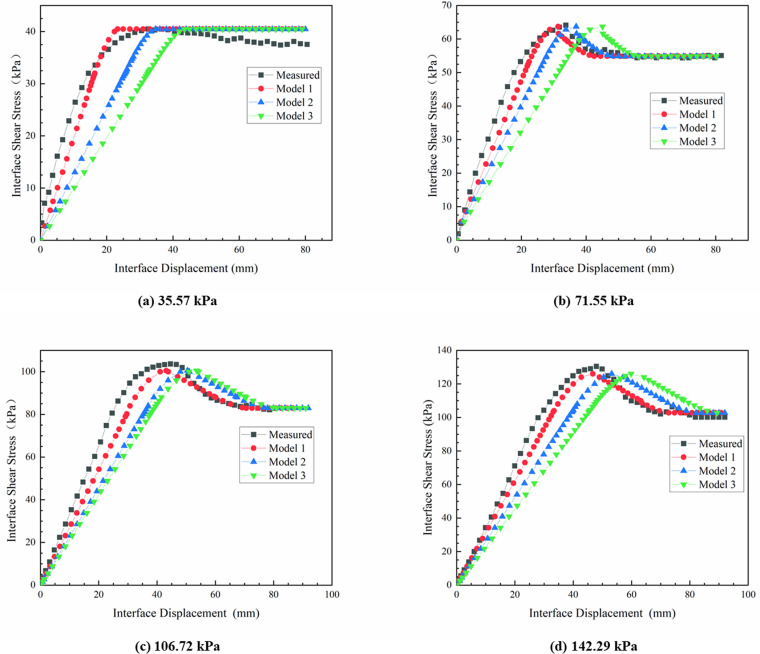
Comparison between tests and models.

The measured curves, along with the curves calculated by each model, under various normal stresses were presented in Fig 15.

As shown in Fig 15, the interface shear stiffness KS1 significantly impacted the accuracy of the progressive failure model. In comparison to Model 2 and Model 3, the computations of Model 1 exhibited a closer alignment with the test data, and reflected the behavior of plastic softening and plastic flow. Therefore, Model 1 was used to predict interfacial shear characteristics of geotextile bags. Additionally, incorporating an elastic-plastic transition stage and a softening-residual transition stage effectively simulated the nonlinear relationship between interface shear stress and displacement observed in pull-out test. In Model 1, while the interface shear stiffness and compression modulus exhibited variations with normal stress, the pre-peak interface characteristic influence factor remained relatively stable, fluctuating only within a narrow range of 3.65 to 3.75. Therefore, it can be inferred that the pre-peak interface characteristic influence factor exhibited a strong inertia to variations in the interface normal stress.

The interface shear strength and residual strength, as computed by each model, were identical, exhibiting a discrepancy of 3% compared to the experimentally measured values. In Model 1, the calculated u1 and u2 were both smaller than the measured. Specifically, when the interface normal stress was 35.57 kPa, the discrepancy in the interface displacement, which corresponded to the peak shear stress, was relatively significant at 16.9%. However, as the normal stress increased to 71.55 kPa, 106.72 kPa, and 142.29 kPa, it gradually decreased to 6.9%, 1.0%, and 2.5%, respectively.

The potential factors contributing to the significant displacement prediction error of Model 1 under low normal stress are analyzed as follows:

1. In comparison to high normal stress, the peak displacement at the interface is smaller. A nonlinear relationship between interfacial shear stress and displacement may have already developed within a displacement range of 20 mm, indicating that the interface has begun to exhibit characteristics of progressive failure.
2. Under conditions of low normal stress, the peak shear stress is not pronounced, and the interface exhibits a gradual softening behavior following the attainment of peak shear stress.

## 5 Discussion on evolution pattern of interface failure

**Fig 16:**
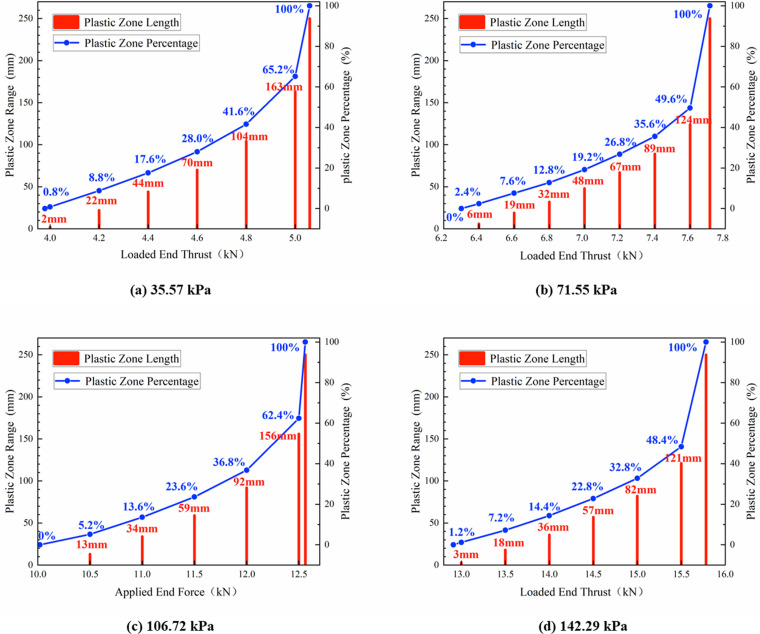
The law of development of interface plastic zone (softening zone) under different normal stresses.

**Fig 17:**
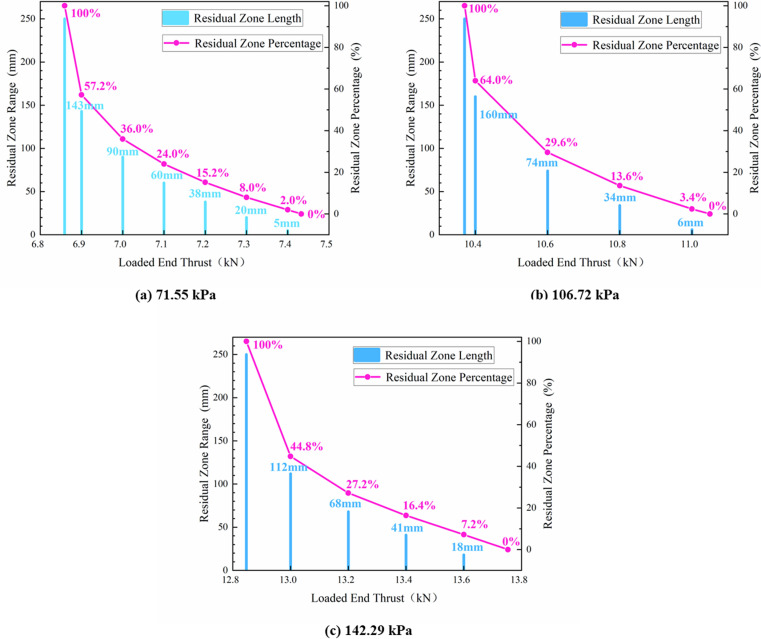
The law of development of interface residual zone under different normal stresses.

To study the evolution patterns of the plastic, softening, and residual zones at the interface during the elastic-plastic (softening) transition stage and the softening-residual transition stage, the lengths of these zones in Model 1 under various nor-mal stresses were calculated using MATLAB. Their development patterns in response to the thrust at the loaded end were depicted in Figs 16 and 17, respectively.

As shown in Fig 16, the length of the plastic (softening) zone exhibited nonlinear development with an increasing thrust, accompanied by a gradual acceleration in its growth rate. Particularly, in close proximity to the critical thrust values, there was a notable increase in the extent of the plastic (softening) zone. When the interface plastic (softening) zone reached 50%, further increases in thrust significantly raised the risk of plastic failure (softening) at the interface. Therefore, the thrust corresponding to a 50% plastic (softening) zone was identified as the upper limit of the early warning thresh-old for plastic failure (softening) at the interface, approximately 95% of the peak thrust.

Under high normal stress, the interface exhibited plastic softening characteristics and gradually transitioned to plastic flow. As depicted in Fig 17, the range of the residual zone at the interface expanded non-linearly during the softening-residual transition stage as the thrust decreased. Following a similar methodology mentioned above, we set the lower limit for the plastic flow early warning threshold at 90% of the peak thrust.

**Fig 18:**
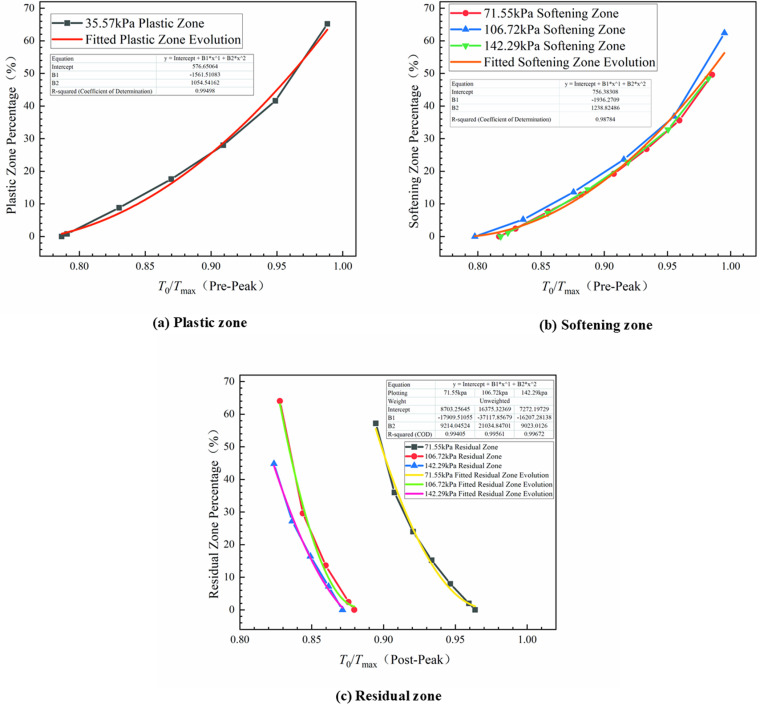
Evolution law of plastic zone, softening zone and residual zone.

To further analyze the evolution patterns of the plastic, softening, and residual zones at the interface, the normalized thrust which was donated by the ratio of thrust to peak thrust was proposed. Then, the relationship between them and their corresponding percentages of plastic, softening, and residual zones was illustrated in Fig 18, which was fitted to a quadratic polynomial with correlation coefficients exceeded 0.99.

Prior to reaching shear strength (when the normalized thrust, donated as *T*_0_/*T*_max_, was approximately 0.8), both plastic and softening zones initiated at the loaded end and extended towards the free end with an accelerating expansion rate as the normalized thrust increased, as shown in Fig 18(a) and 18(b). The evolution pattern of the softening zone remained fundamentally consistent despite variations in normal stresses, as illustrated in Fig 18(b). By successfully fitting a quadratic curve to this evolution pattern across various normal stresses, we attained a high correlation coefficient of 0.98. This suggested that the evolution was generally consistent and demonstrated considerable resistance to variations in normal stress within the context of the trilinear softening model. To some extent, this verified the conclusion drawn in 3.3: the pre-peak interface characteristic influence factor shown a strong inertia to the normal stress.

Following the attainment of peak shear stress, plastic softening ensued, subsequently transitioning into a state of plastic flow. As illustrated in Fig 18(c), it was evident that throughout the development of the residual zone, the normalized thrust consistently hovered around 0.06 while demonstrating a relatively uniform rate of progression. However, normal stress exerted a significant influence on this evolutionary process. As thrust gradually diminished, the interface transitioned into the residual phase at a normal stress level of 71.55 kPa, with *T*_0_/*T*_max_ approximately equal to 0.96. In contrast, at normal stresses of 106.72 kPa and 142.29 kPa, the interfaces remained within the fully softened phase and entered the residual phase at *T*_0_/*T*_max_ values of 0.88 and 0.87 respectively. This observation indicated that lower normal stresses precipitated an earlier onset of residual zone evolution and resulted in enhanced residual strength by the conclusion of this evolution.

## 6 Conclusions

In this study, to improve and reveal the interfacial shear characteristics of geotextile bags filled with fine tailings, pull-out tests were conducted on geotextile bags filled with varying cement contents. An interfacial progressive failure model was proposed, validated, and analyzed. The following conclusions were drawn:

1. The theoretical displacement difference between the loaded end and the free end of the geotextile bag was derived and subsequently compared with experimentally measured values, thereby confirming progressive failure at the interface. The interfacial shear strength can be described using Coulomb formula for soil, primarily influenced by both apparent friction angle and apparent cohesion; however, the former played a dominant role. To reduce construction costs while improving interface strength in geotextile bags, utilizing sample S_3_ to fill geotextile bags can achieve satisfactory results, whose apparent friction angle was increased 17.4% compared with geotextile bags filled with S_0_.
2. Through force analysis of the geotextile bag interface, a control equation was established for geotextile bag interface. Both elastic-plastic model and trilinear softening model were refined to accurately depict progressive failure characteristics under low normal stress (<60 kPa) and high normal stress (≥60 kPa). These models provided analytical solutions for the thrust, shear stress, and displacement at the mold-bag interface.
3. The pre-peak interface shear stiffness (*K*_S1_) was determined from the slope of a fitted line representing displacement within 20 mm against corresponding shear stress. Additionally, dynamic compression modulus, which varied with interfacial normal stress, was obtained through linear fitting. Taking the experimental data of geotextile which was filled with sample S_3_ and consolidated for 7 days as an example, the reliability and applicability of the model was verified; furthermore, it indicated that pre-peak interface characteristic influence factor exhibited significant inertia towards variations in normal stress.
4. The evolution patterns of the plastic, softening, and residual zones at the geotextile bag interface were revealed. By establishing a threshold at 50% development of these zones, empirical values for the early warning loads associated with plastic damage, plastic softening, and plastic flow at the interface were ascertained. Additionally, an empirical formula relating the development of the plastic, softening, and residual zones to normalized thrust was proposed.

## Supporting information

S1 Data All raw experimental data plots reported in this research can be reconstructed using the primary data files archived in “S1 Data”, ensuring complete traceability of results. (ZIP)

S1 Fig All raw experimental data plots, visual records of testing procedures, and apparatus schematics are compiled in “S1 Fig” as supporting documentation for this research. (ZIP)

## References

[1] Li Q, Ma G, Lu Y. An experimental and theoretical study on the tailings dam with geotextile bags. Sustainability. 2023;15(6):4768. doi: 10.3390/su15064768

[2] Zhang J, Song Z, Geng W. Analysis and prevention of tailings dam accidents in China. Modern Mining. 2023;3: 6–9. (in Chinese) doi: 10.3969/j.issn.1674-6082.2023.03.002

[3] Finklenburg B, Klopries E-M, Schüttrumpf H. 2D failure mechanisms and failure modes of a new type of geotextile tubes used for river dikes. Geotextiles and Geomembranes. 2024;52(4):690–703. doi: 10.1016/j.geotexmem.2024.03.009

[4] Wu H, Tian Z, Shu Y, et al. Experimental study on the dewatering characteristics of slurry-filled geotextile tube using hanging bag model. Journal of Tianjin University. 2021;5:487–96. doi: 10.11784/tdxbz202004075

[5] Lee EC, Douglas RS. Geotextile tubes as submerged dykes for shoreline management in Malaysia. Geotextiles and Geomembranes. 2012;30:8–15. doi: 10.1016/j.geotexmem.2011.01.003

[6] Tseng I-F, Hsu C-H, Cheng H-C, Chen Y-S. Application of geotextile tubes to coastal silt mitigation: a case study in Niaoyu fishing harbor. Sustainability. 2024;15(3):2024. doi: 10.3390/su15032024

[7] Sheng M, Zhu Q, Huang F, Zou C, Gao Y, Liu X. Study on the formation and dewatering process of the surface filter cake of geotextile on the lateral boundary of geotextile tubes under constant flow grouting. Front Earth Sci. 2024;12:1427659. doi: 10.3389/feart.2024.1427659

[8] Du C, Li D, Yi F, Wang L, Niu B. Analysis of interface mechanical properties between geotextiles and tailings during pull-out tests. PLoS One. 2022;17(10):e0276543. doi: 10.1371/journal.pone.0276543 36288404 PMC9605312

[9] Liu X, Wu Z, Guo F. Experimental study on the shear strength of different interfaces of fine-grained-tailing-filled geotextile tubes. Buildings. 2024;14(7):1934. doi: 10.3390/buildings14071934

[10] Liu J, Pan J, Liu Q, Xu Y. Experimental study on the interface characteristics of geogrid-reinforced gravelly soil based on pull-out tests. Sci Rep. 2024;14(1):8669. doi: 10.1038/s41598-024-59297-9 38622251 PMC11018815

[11] Zhao X, Gao P, Liu W, Yang Y, Meng L, Yang G, et al. Research on the interface characteristics of coal gangue with different geosynthetic reinforcements. Matéria (Rio J). 2024;29(2):e20240012. doi: 10.1590/1517-7076-rmat-2024-0012

[12] Carrubba P. Laboratory evaluation of geosynthetic interface friction under low stress. Polymers (Basel). 2024;16(17):2519. doi: 10.3390/polym16172519 39274151 PMC11398199

[13] Nguyen M-D, Ho M-P. The influence of saturation on the interface shear strength of clay and nonwoven geotextile. Journal of Science and Technology in Civil Engineering, 2021, 15(1):41–54. doi: 10.31814/stce.nuce2021-15(1)-04

[14] Liu X, Wu Z, He H, Xu Q. An experimental study focusing on the filling process and consolidation characteristics of geotextile tubes filled with fine-grained tungsten tailings. Sustainability. 2024;16(12):5270. doi: 10.3390/su16125270

[15] Zhang H, Sun H, Liu S, Geng X, Deng Y, Cai Y. Combined vacuum-assisted geotextile and geomembrane tubes for sludge dewatering: a theoretical switching point. Can Geotech J. 2023;61(5):978–91. doi: 10.1139/cgj-2022-0370

[16] Li H, Yi F, Jiang X, et al. Effect test of low cement content on filling characteristics of mold bag. Advances in Science and Technology of Water Resources. 2020;40(3):72–7.

[17] Cong H, Wang Q, Xia Z. Application of precision dosing system in geotextile bag solidification and dredging mud engineering. Jiangsu Water Resources. 2024;07(11):1–15.

[18] He P, Ma W, Mu Y. Experimental study and constitutive model of shear characteristics at the loess-mortar block interface. Rock and Soil Mechanics. 2019;40(1):82–90,98.

[19] Chen C, Wen Y, Zhu S. Shear creep model of anchor-soil interface considering shear stress level and soil dry density. Journal of Hunan University. 2021;48:1–9.

[20] Du C, Yi F. Full-process analysis of the elasto-plastic model for the reinforcement-soil pullout interface. Journal of China Coal Society. 2020;45(12):4062–73. doi: 10.13225/j.cnki.jccs.2019.1419

[21] Chen R, Li B, Hao D. Simulation method for the interaction of geogrid-reinforced soil interface based on the cohesion model. Chinese Journal of Geotechnical Engineering. 2020;42(5):934–40. doi: 10.11779/CJGE202005016

[22] Huang M, Zhou Z, Ou J. Nonlinear full-process analysis of the pullout force on the anchoring section of tension anchors. Chinese Journal of Rock Mechanics and Engineering. 2014;33(11):2190–9.

[23] Pierozan RC, Araujo GLS, Palmeira EM, Romanel C, Zornberg JG. Interface pullout resistance of polymeric strips embedded in marginal tropical soils. Geotextiles and Geomembranes. 2021;50(1):20–39. doi: 10.1016/j.geotexmem.2021.08.004

[24] Gao W, Lin Y, Wang X, Zhou T, Zheng C. Interface mechanics of double-twisted hexagonal gabion mesh with coarse-grained filler based on pullout test. Materials (Basel). 2023;17(1):164. doi: 10.3390/ma17010164 38204017 PMC10779748

[25] Xu L, Tang X, Shen H. Study on the distribution pattern of pullout force in reinforced soil structures. Chinese Journal of Geotechnical Engineering. 2013;35(4):800–4.

[26] Huang C-C, Hsieh H-Y, Hsieh Y-L. Hyperbolic models for a 2-D backfill and reinforcement pullout. Geosynthetics International. 2014;21(3):168–78. doi: 10.1680/gein.14.00007

[27] Makkar FM, Chandrakaran S, Sankar N. Performance of 3-D geogrid-reinforced sand under direct shear mode. International Journal of Geotechnical Engineering. 2019;13(3):227–35. doi: 10.1080/19386362.2017.1336297

[28] Cheng H, Wang X, Zhang J. Study on the shear characteristics and statistical damage softening model of reinforced coarse-grained soil interfaces. Journal of Railway Science and Engineering. 2018;15(11). doi: 10.19713/j.cnki.43-1423/u.2018.11.008

[29] Zhang C, Zhu H, Tang C. Progressive failure model of fiber-reinforced soil interface. Journal of Zhejiang University. 2015;49:1952–9. doi: 10.3785/j.issn.1008-973X.2015.10.018

[30] Li L, Lai F, Chen F. Theoretical analysis of the progressive pullout behavior of geosynthetic-reinforced soil. Interfaces Nonferrous Metals (Mining Section). 2016;68(4):74–80. doi: 10.3969/j.issn.1671-4172.2016.04.018

[31] Cao Y, Peng F, Xiao Z, et al. Numerical analysis of progressive deformation and failure of reinforced sand slopes. Chinese Journal of Rock Mechanics and Engineering. 2010;29(S2):3905–15.

[32] Hong C-Y, Yin J-H, Zhou W-H, Pei H-F. Analytical Study on Progressive Pullout Behavior of a Soil Nail. J Geotech Geoenviron Eng. 2012;138(4):500–7. doi: 10.1061/(asce)gt.1943-5606.0000610

[33] Zhu H-H, Zhang C-C, Tang C-S, Shi B, Wang B-J. Modeling the pullout behavior of short fiber in reinforced soil. Geotextiles and Geomembranes. 2014;42(4):329–38. doi: 10.1016/j.geotexmem.2014.05.005

[34] Chen J, Saydam S, Hagan PC. An analytical model of the load transfer behavior of fully grouted cable bolts. Construction and Building Materials. 2015;101:1006–15. doi: 10.1016/j.conbuildmat.2015.10.099

[35] Lai F, Li L, Chen F. Elastic-exponential softening model and behavior of the pullout interface in geogrid reinforced soil. Journal of Engineering Geology. 2018;26(4):825–60. doi: 10.13544/j.cnki.jeg.2017-199

[36] Fu S, Liu X, Liu X. An experimental study of the mechanical properties of tailings filling mold bag. Hydrogeol Eng Geol. 2018;45(1):83–8.

[37] Yi F, Li H, Zhang J, Jiang X, Guan M. Experimental Studies on Interfacial Shear Characteristics between Polypropylene Woven Fabrics. Materials (Basel). 2019;12(22):3649. doi: 10.3390/ma12223649 31698741 PMC6888349

