# Supplementary figures and images for: Study on interfacial shear characteristics and progressive failure model of geotextile bags
Source: PLoS One. 2025 Jun 2;20(6):e0321058. doi: 10.1371/journal.pone.0321058 (PMC12129224; doi:10.1371/journal.pone.0321058)

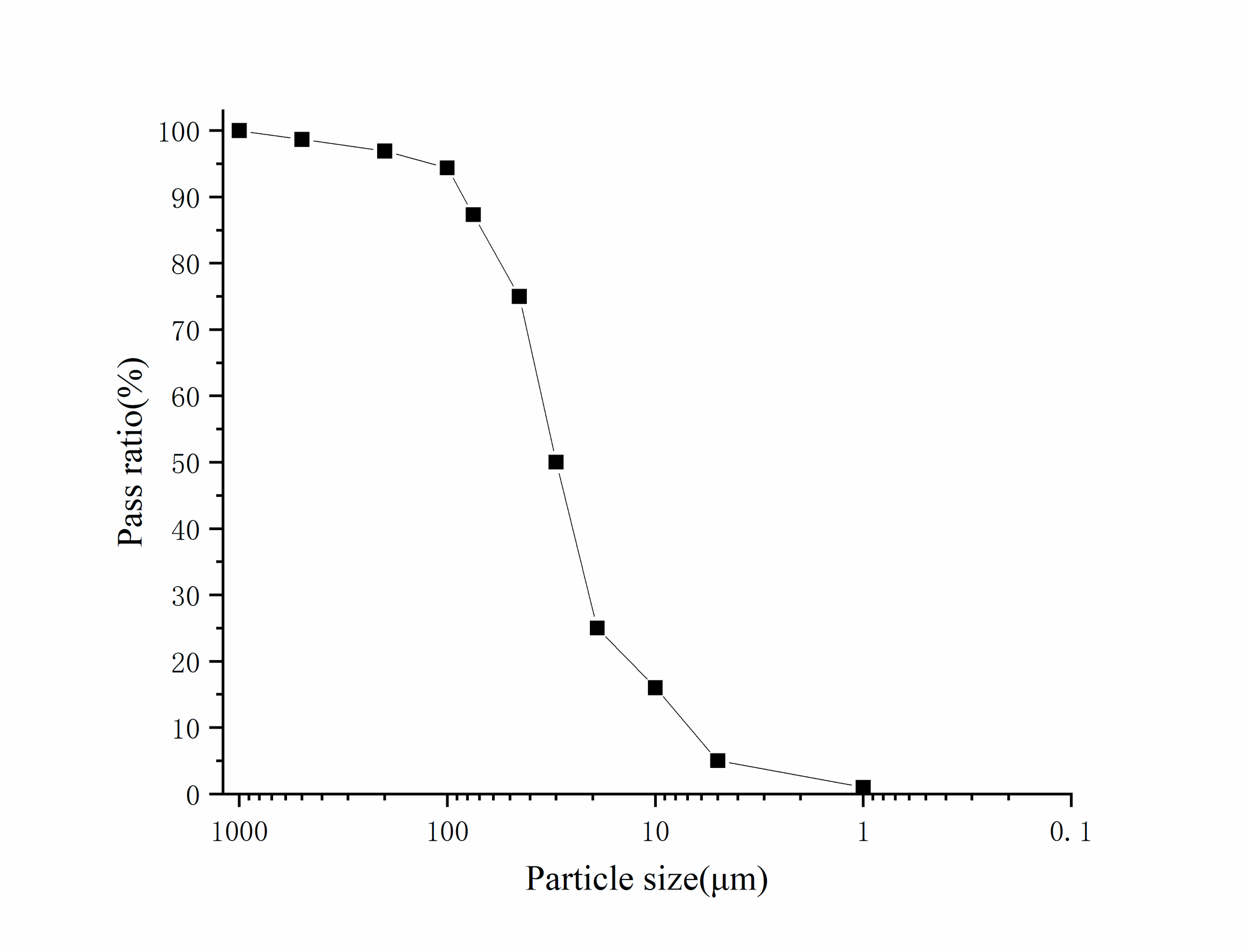

Supplement: S1 Fig — (ZIP) [file pone.0321058.s002.zip › S1 Figures/Figure 1.tif]

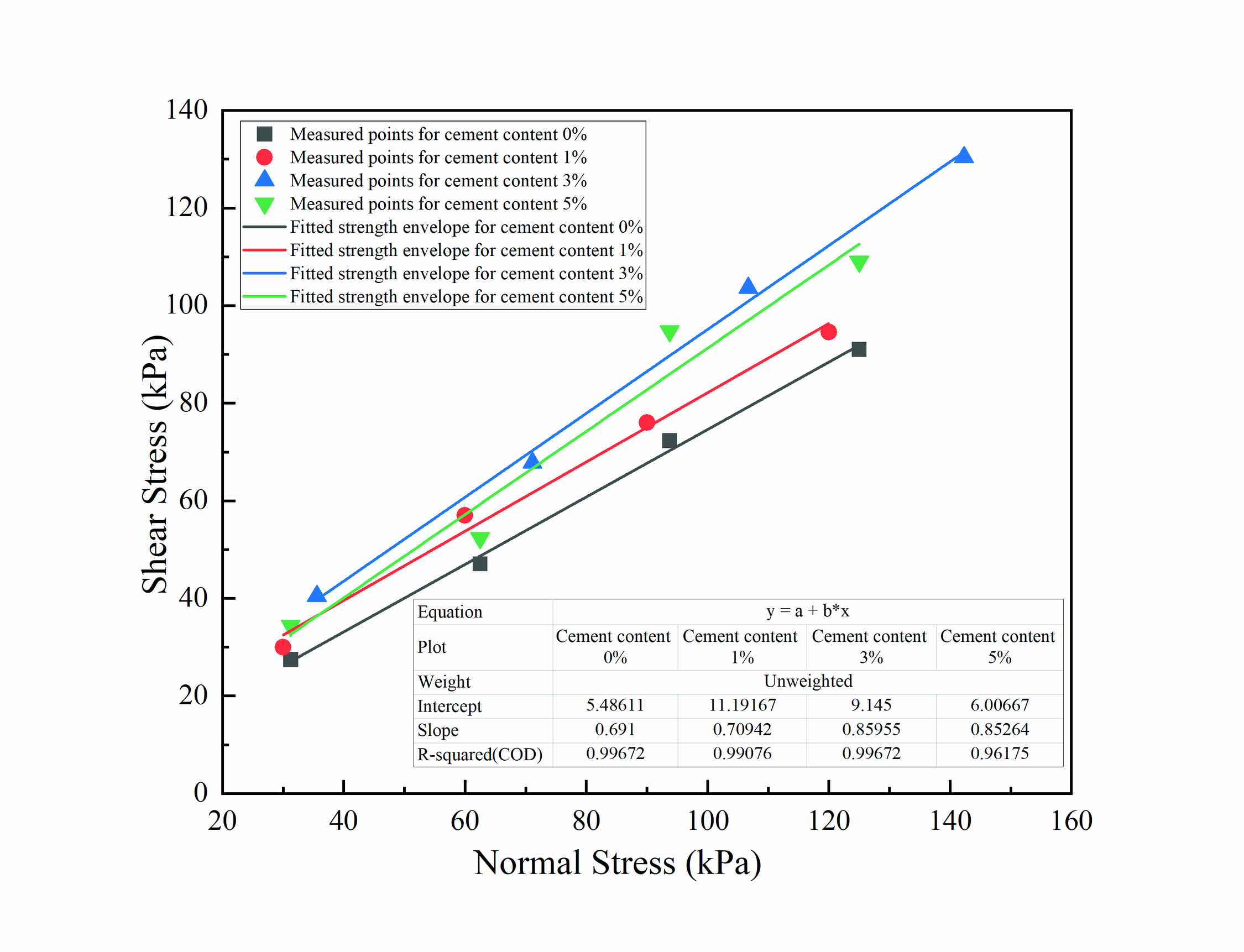

Supplement: S1 Fig — (ZIP) [file pone.0321058.s002.zip › S1 Figures/Figure 10.tif]

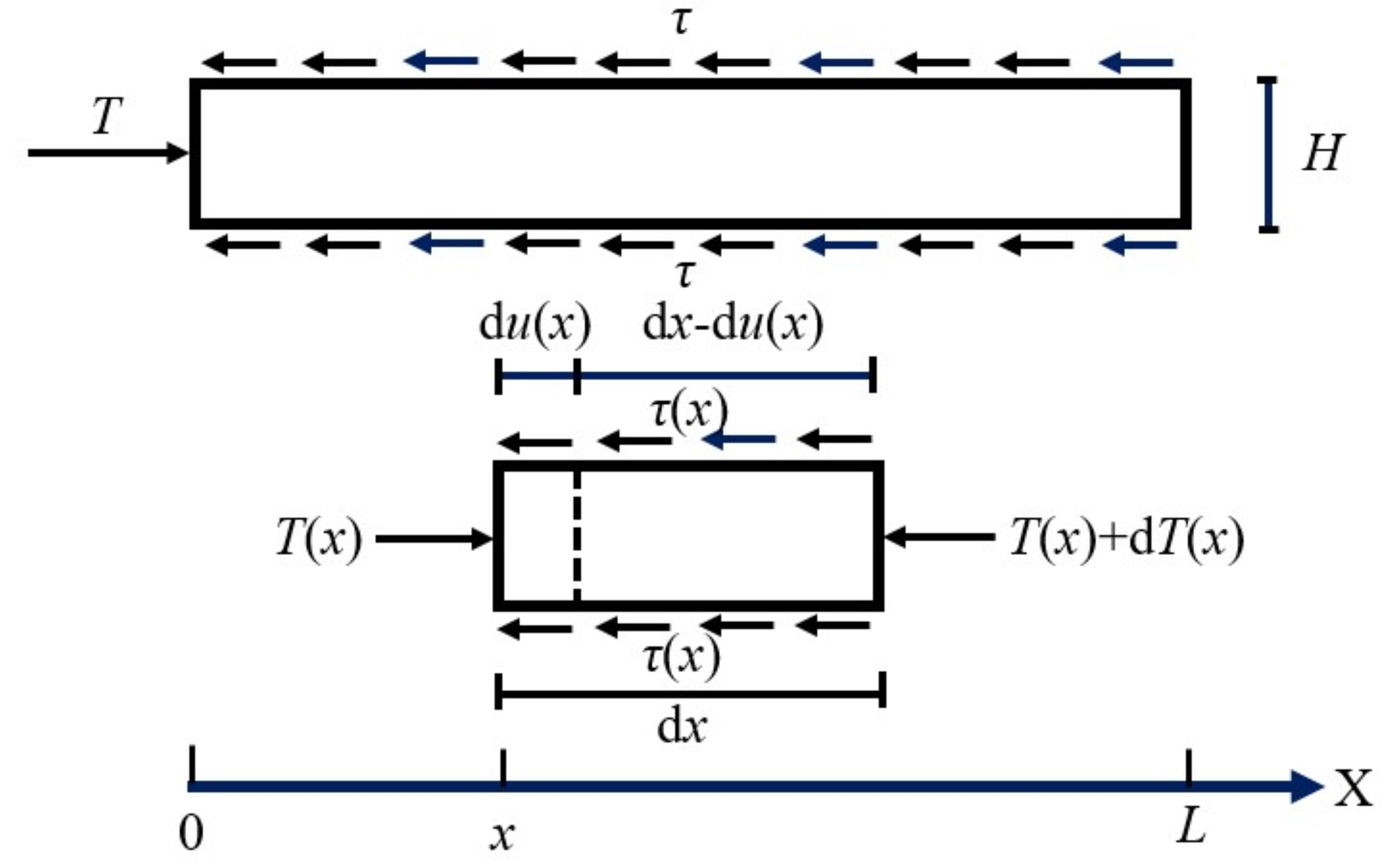

Supplement: S1 Fig — (ZIP) [file pone.0321058.s002.zip › S1 Figures/Figure 11.tif]

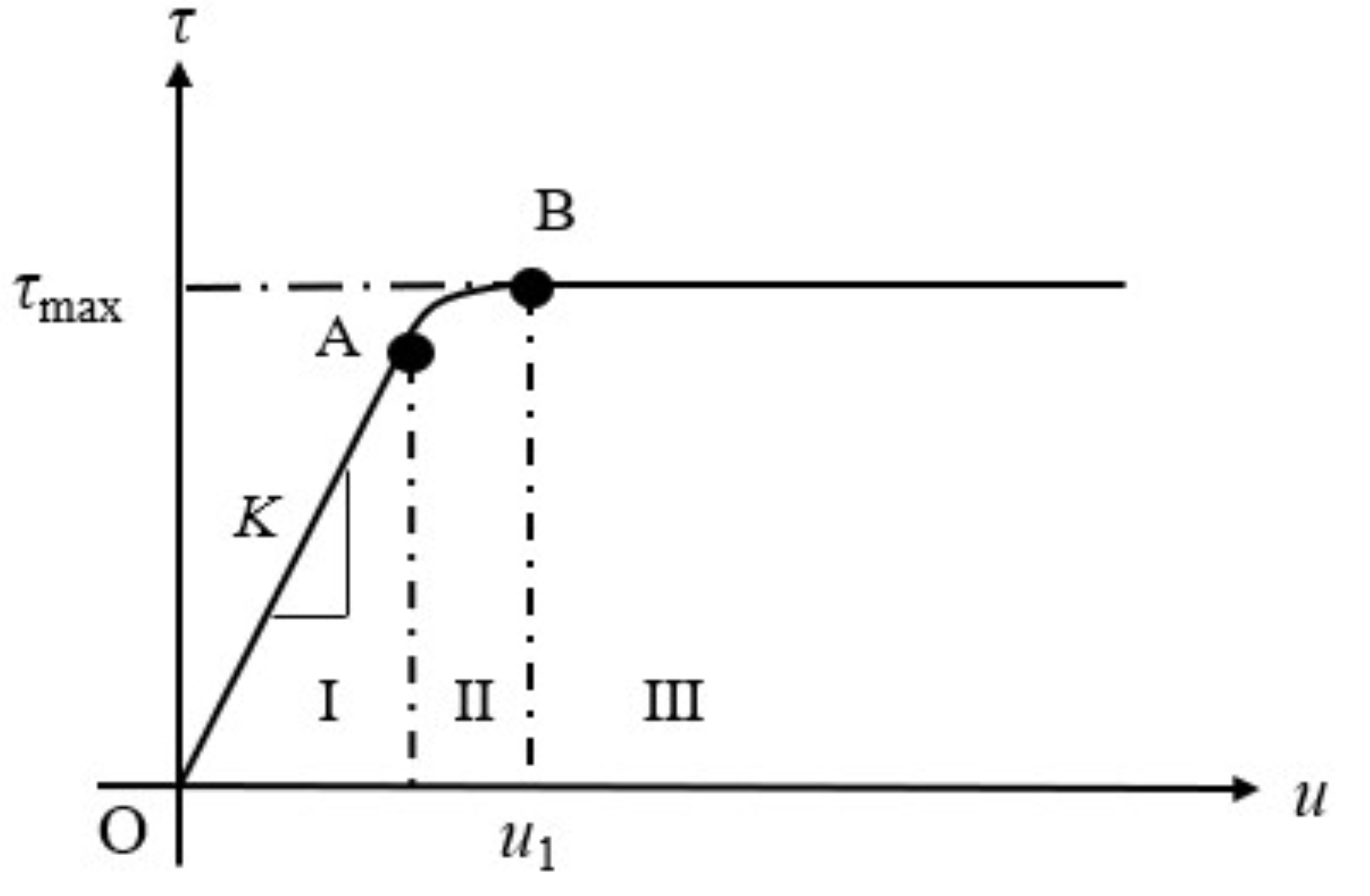

Supplement: S1 Fig — (ZIP) [file pone.0321058.s002.zip › S1 Figures/Figure 12 (a).tif]

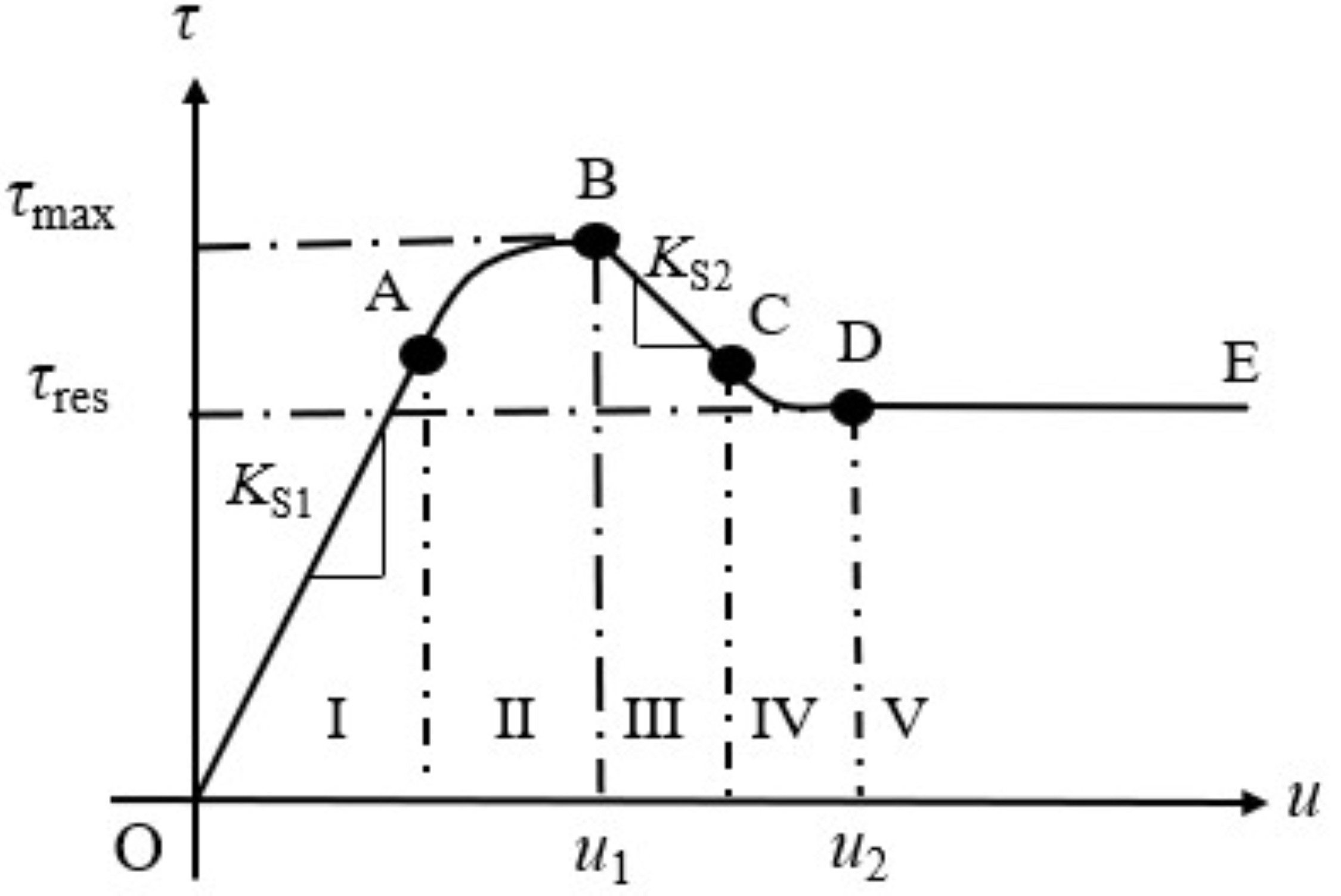

Supplement: S1 Fig — (ZIP) [file pone.0321058.s002.zip › S1 Figures/Figure 12 (b).tif]

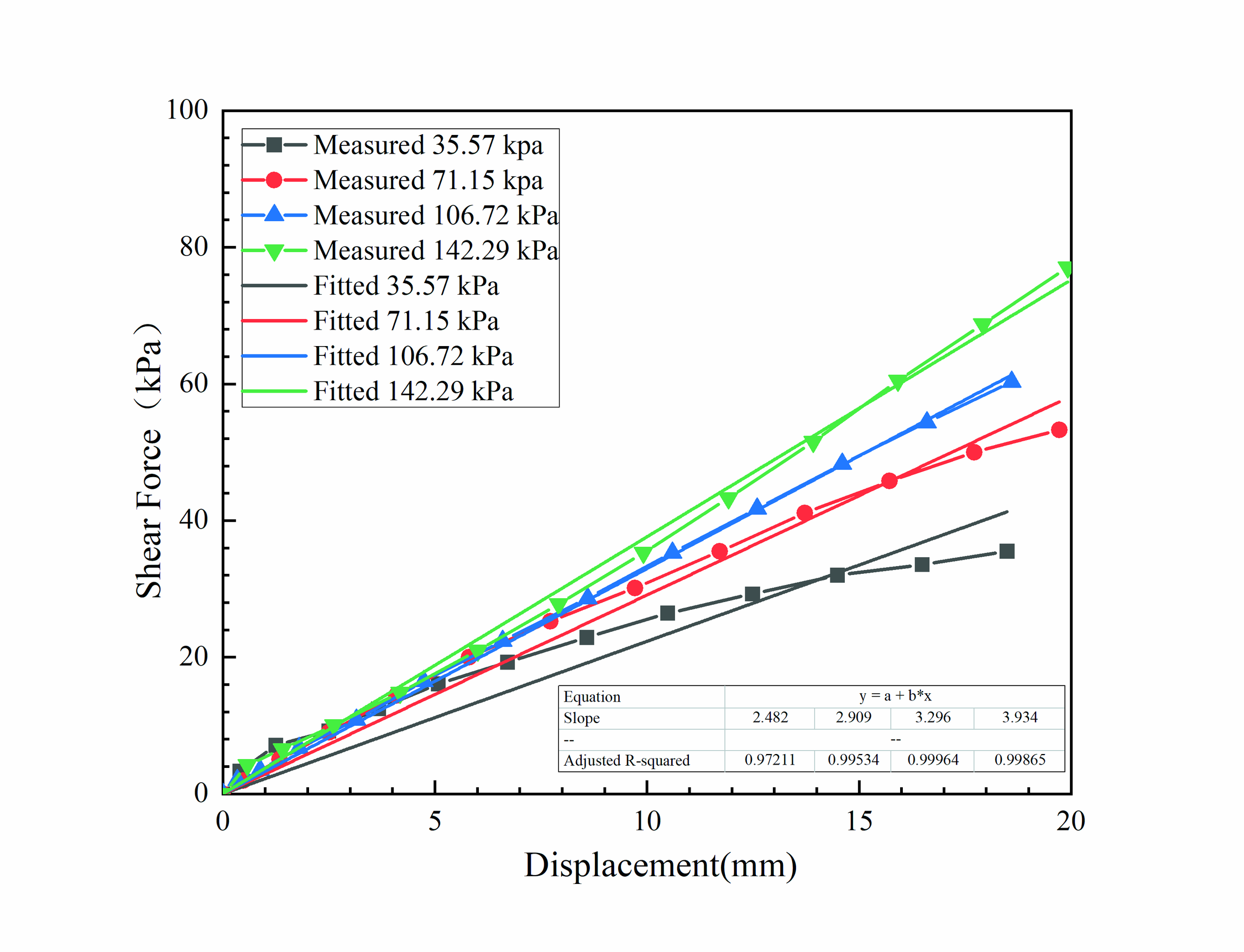

Supplement: S1 Fig — (ZIP) [file pone.0321058.s002.zip › S1 Figures/Figure 13.(a).tif]

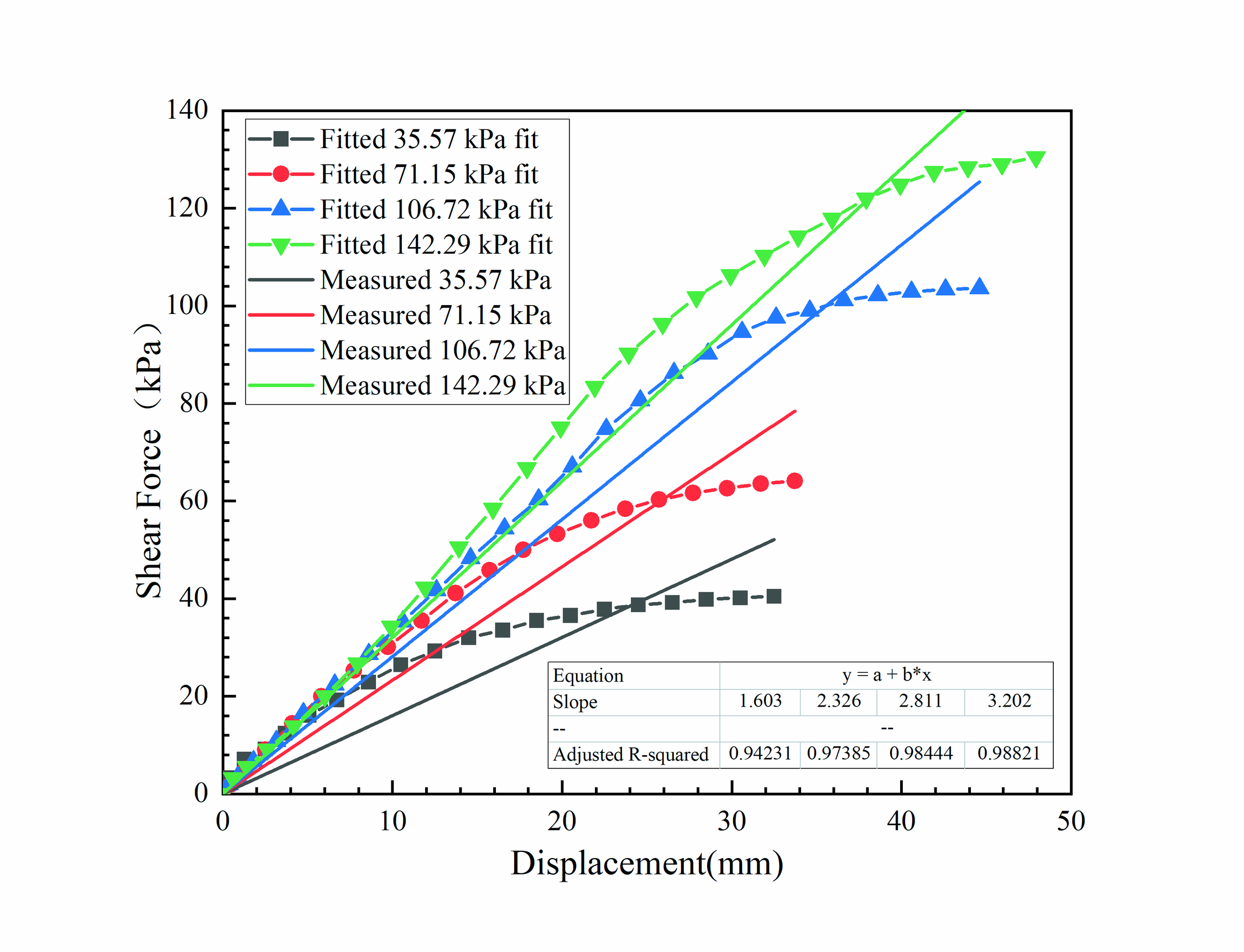

Supplement: S1 Fig — (ZIP) [file pone.0321058.s002.zip › S1 Figures/Figure 13.(b).tif]

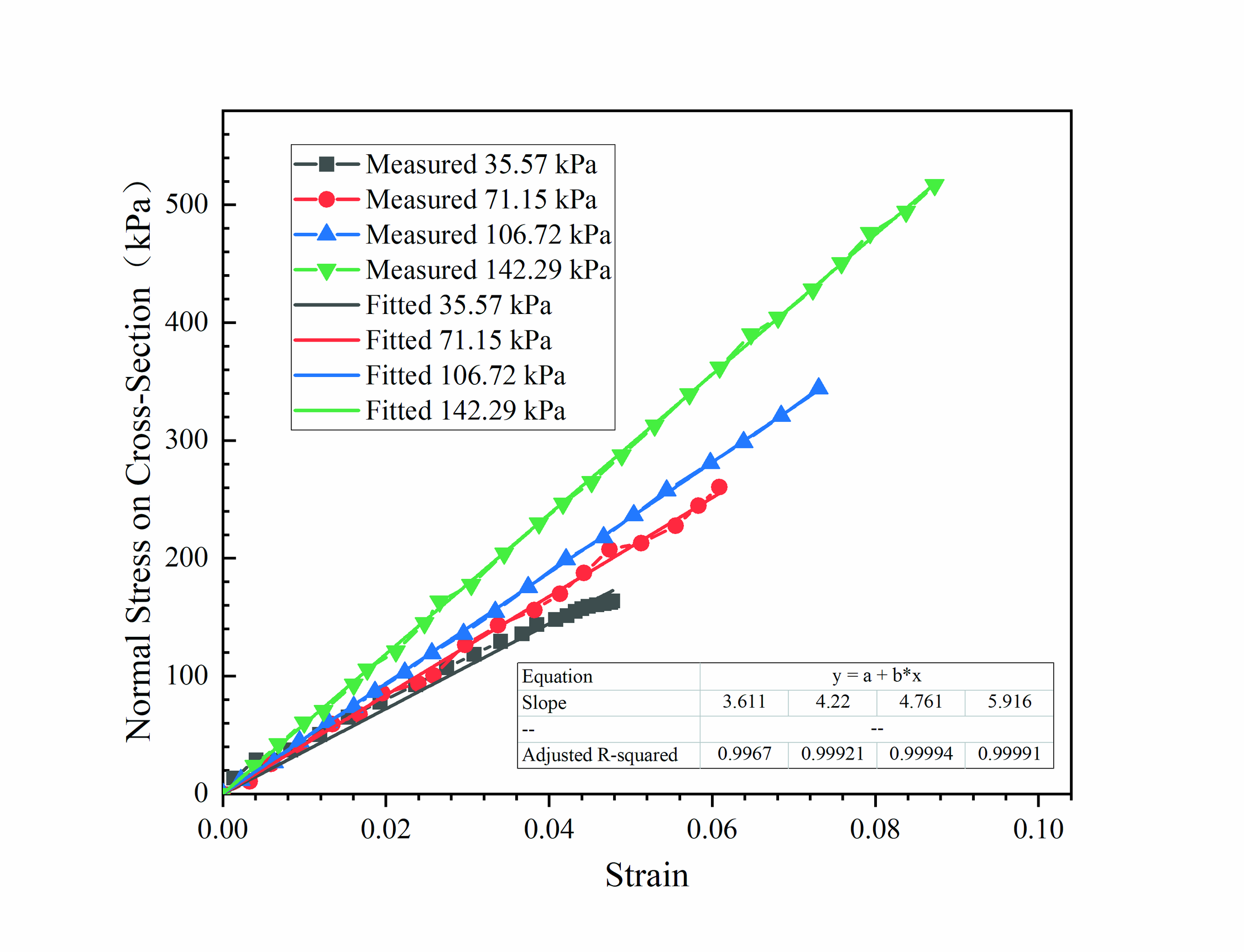

Supplement: S1 Fig — (ZIP) [file pone.0321058.s002.zip › S1 Figures/Figure 14.tif]

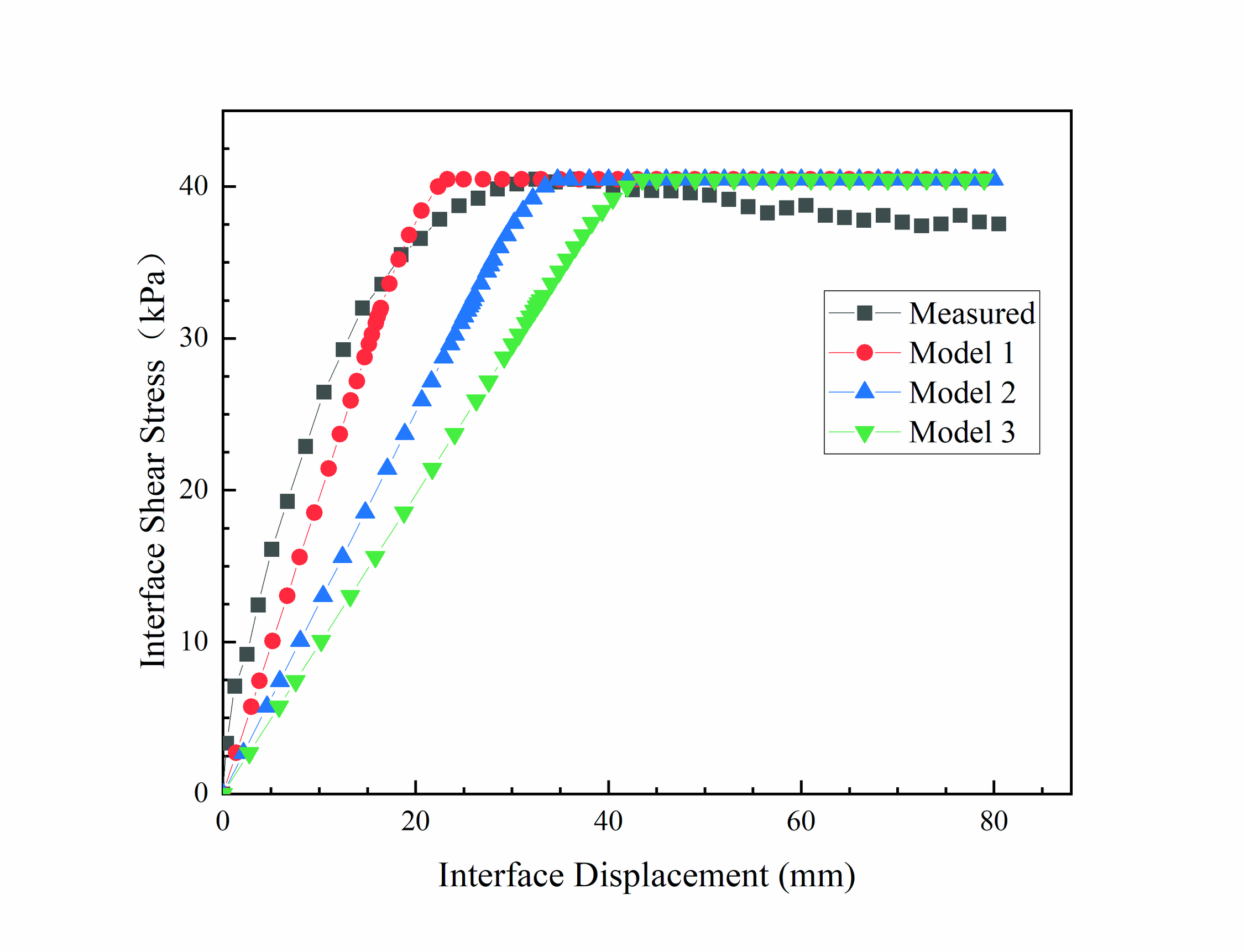

Supplement: S1 Fig — (ZIP) [file pone.0321058.s002.zip › S1 Figures/Figure 15 (a).tif]

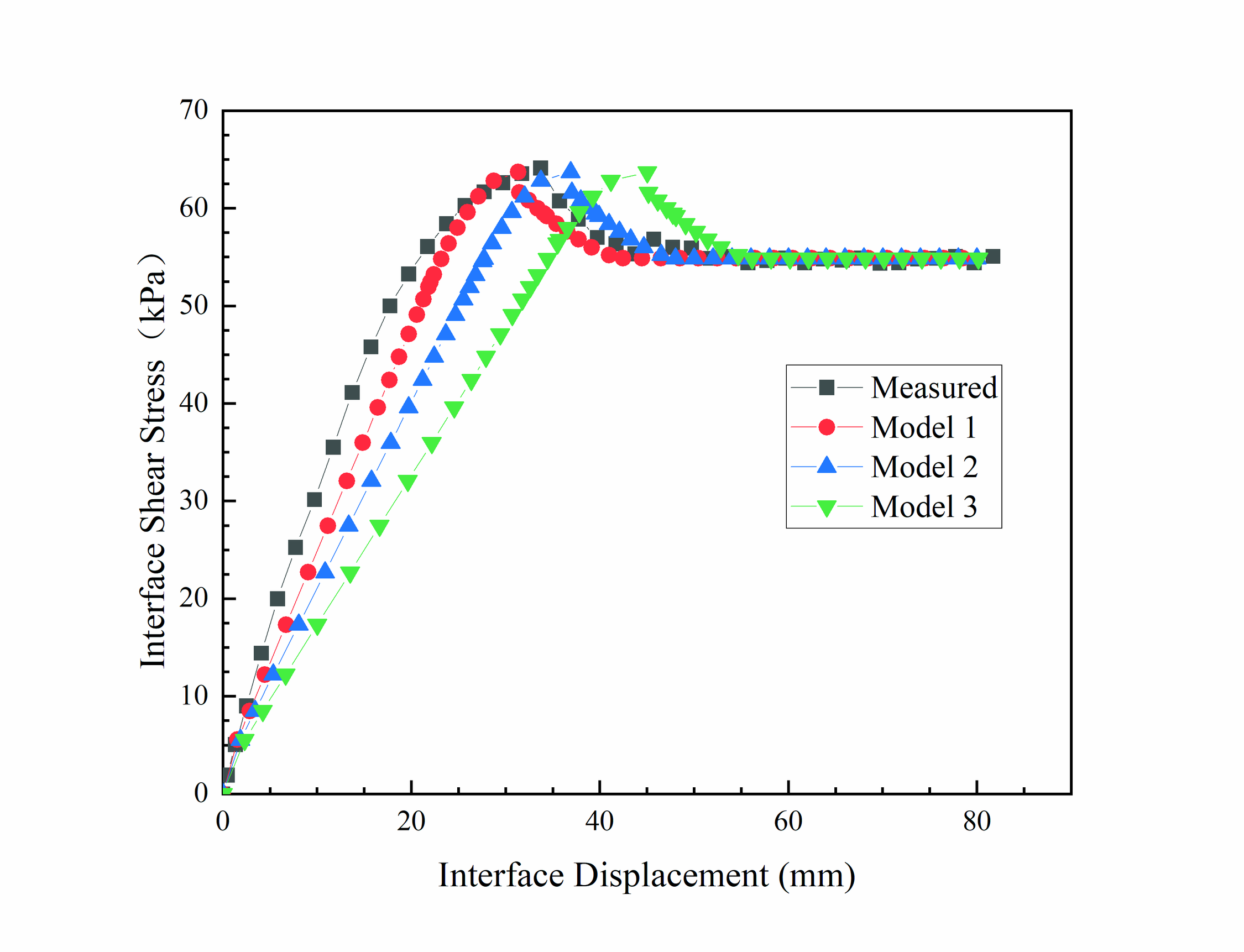

Supplement: S1 Fig — (ZIP) [file pone.0321058.s002.zip › S1 Figures/Figure 15 (b).tif]

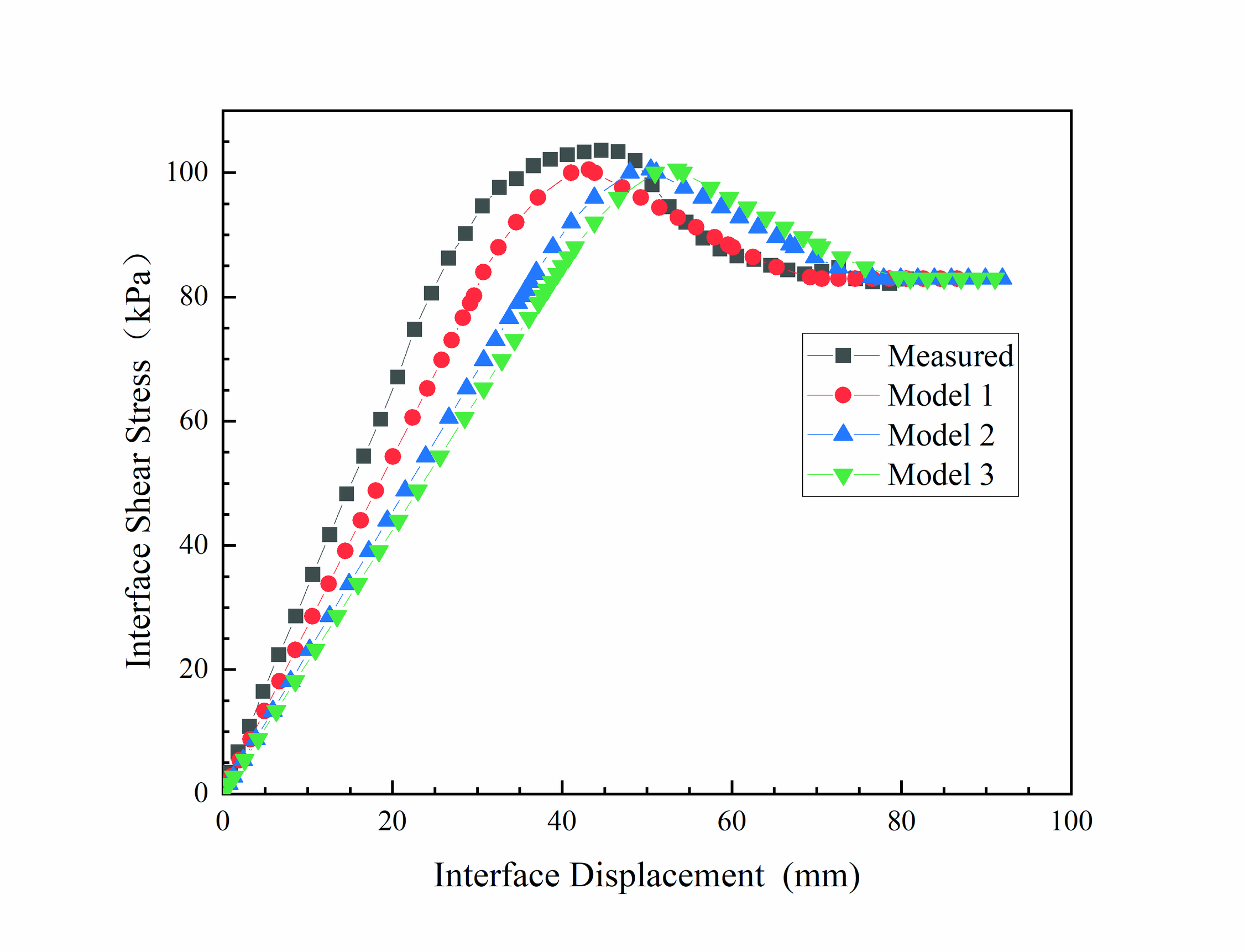

Supplement: S1 Fig — (ZIP) [file pone.0321058.s002.zip › S1 Figures/Figure 15 (c).tif]

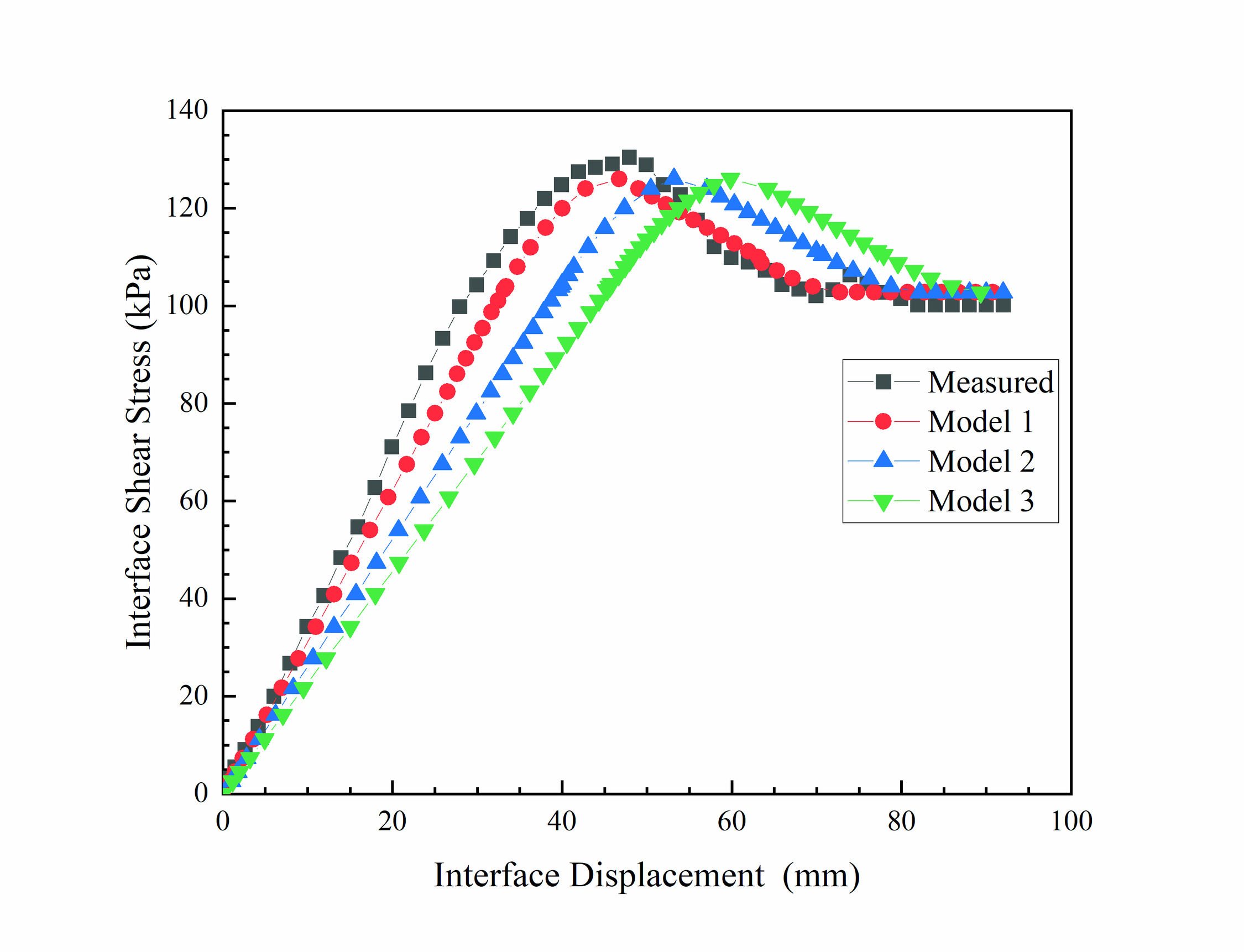

Supplement: S1 Fig — (ZIP) [file pone.0321058.s002.zip › S1 Figures/Figure 15 (d).tif]

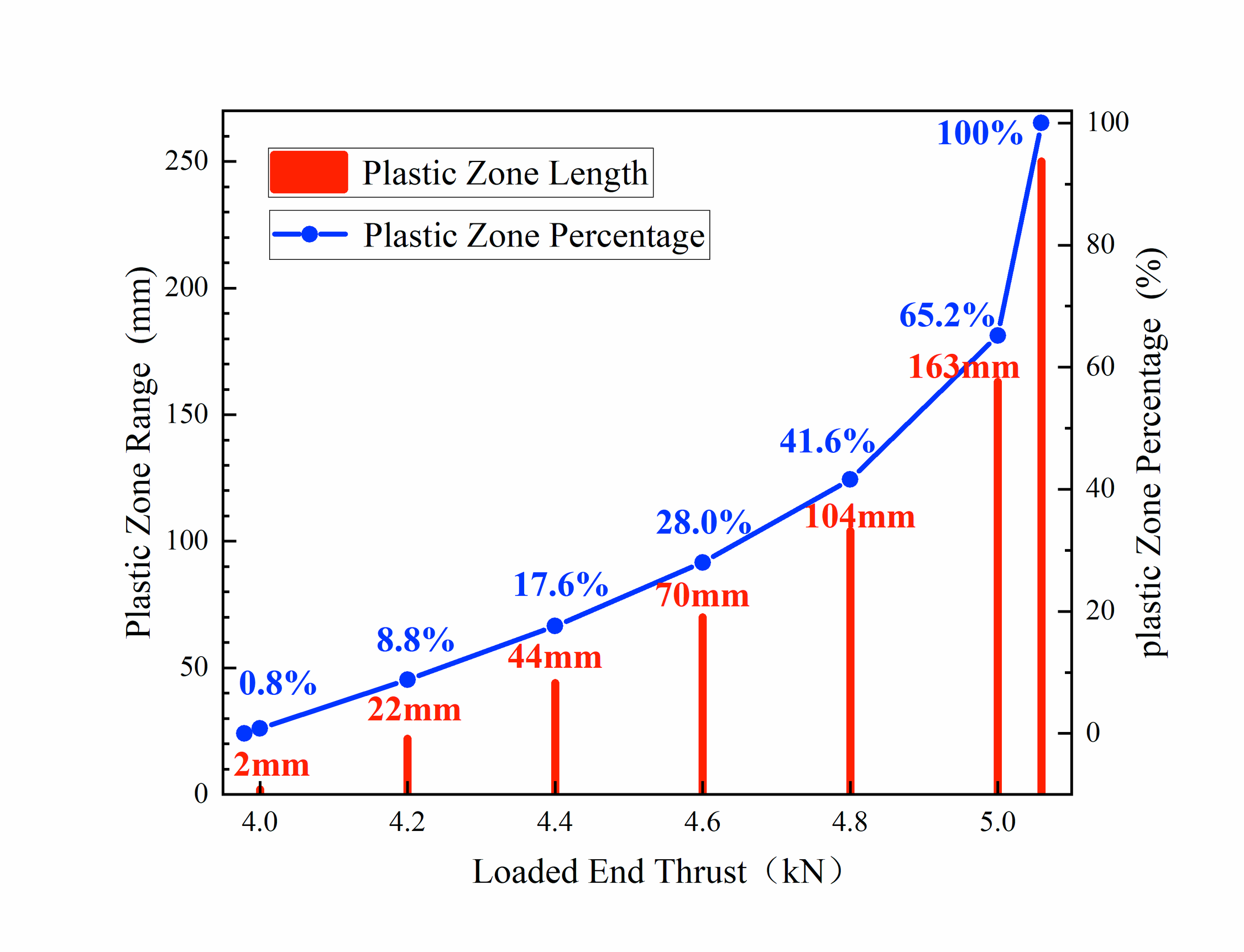

Supplement: S1 Fig — (ZIP) [file pone.0321058.s002.zip › S1 Figures/Figure 16 (a).tif]

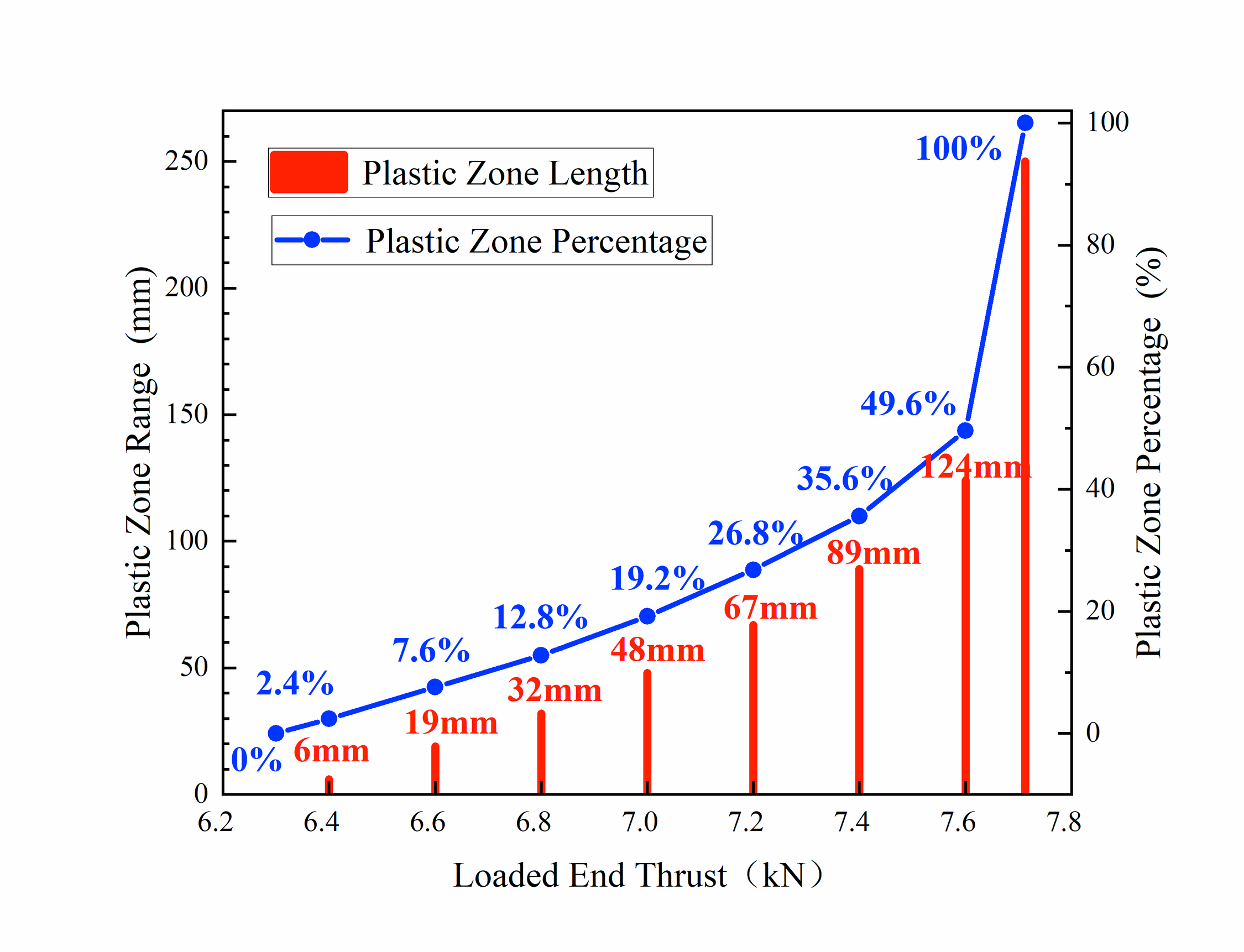

Supplement: S1 Fig — (ZIP) [file pone.0321058.s002.zip › S1 Figures/Figure 16 (b).tif]

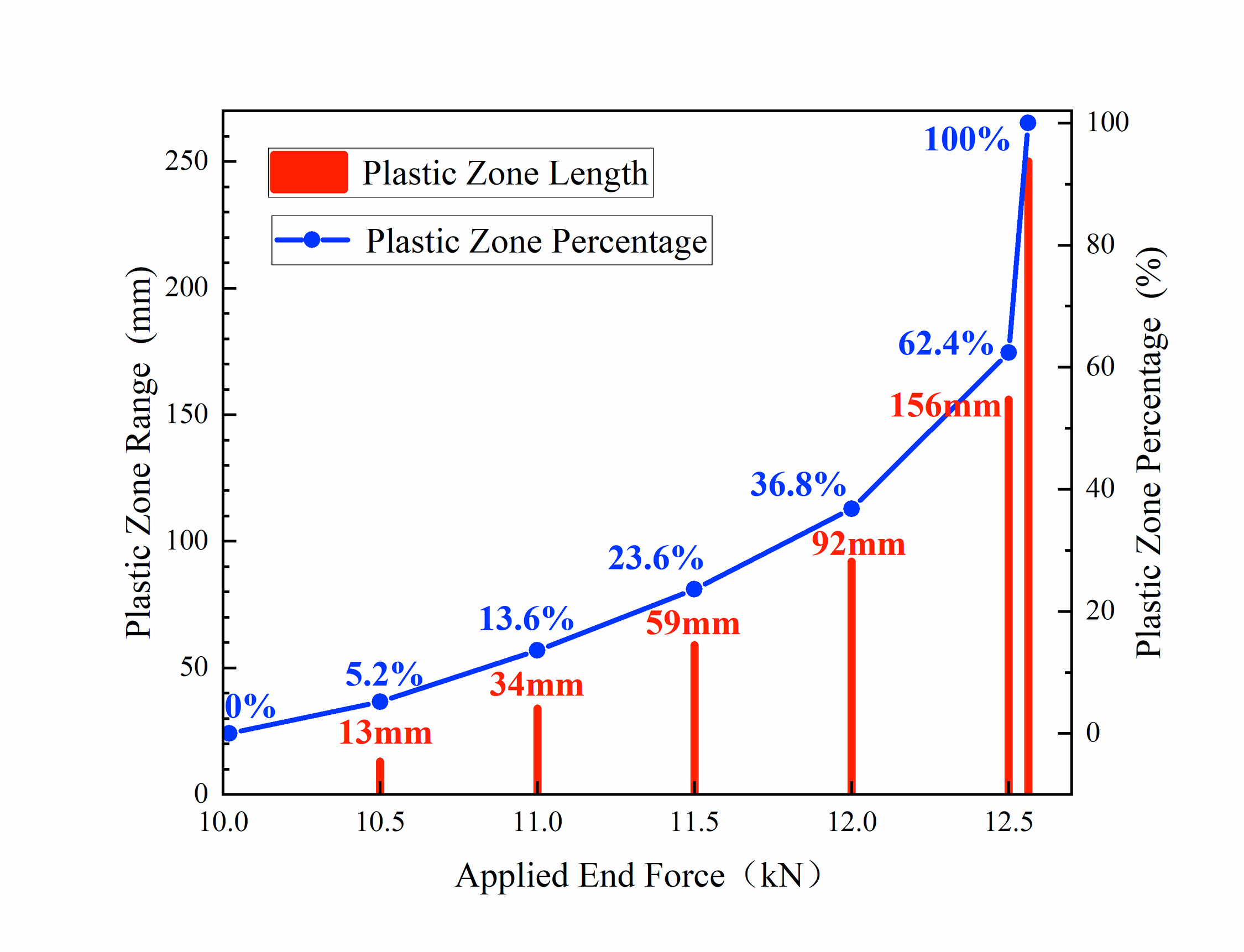

Supplement: S1 Fig — (ZIP) [file pone.0321058.s002.zip › S1 Figures/Figure 16 (c).tif]

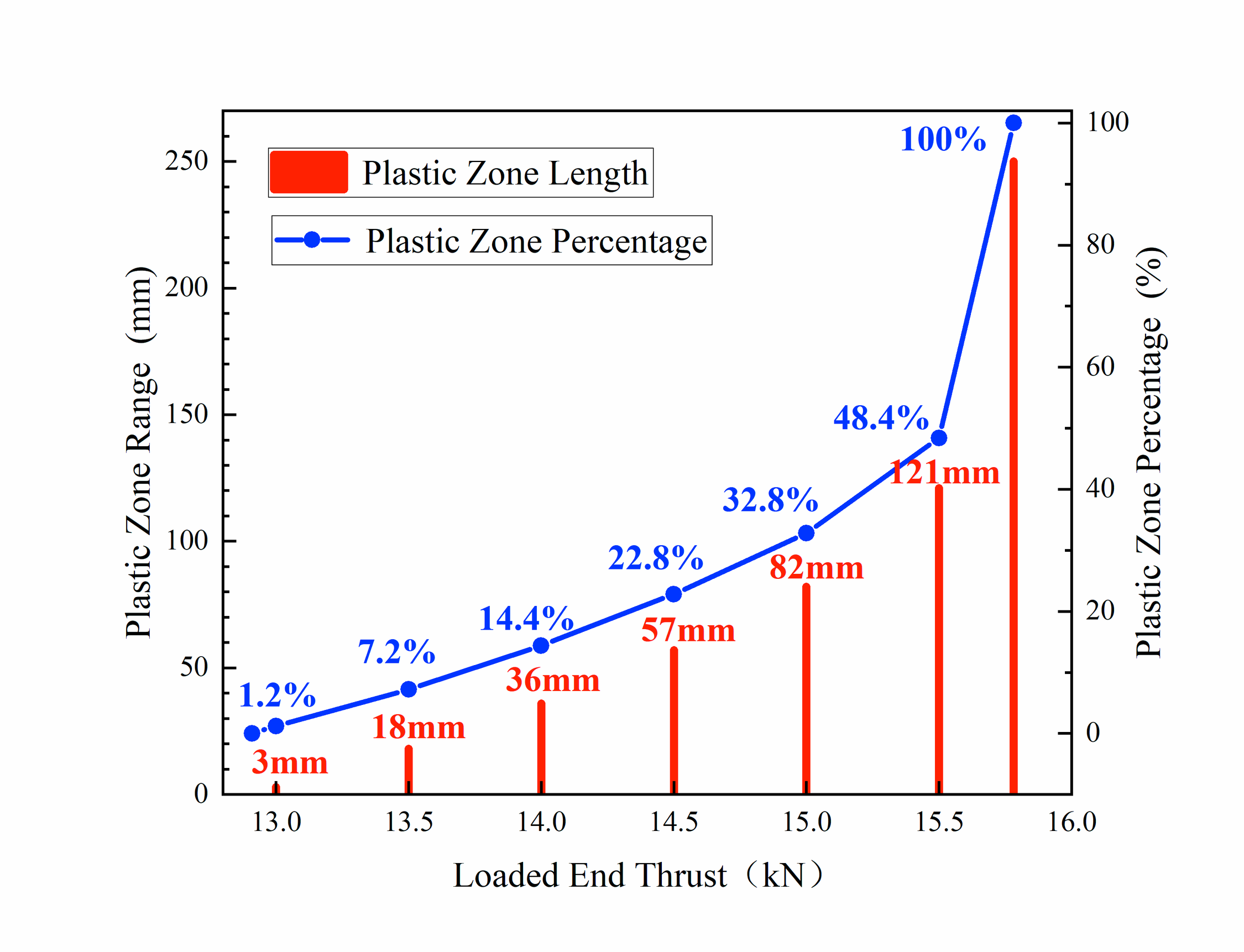

Supplement: S1 Fig — (ZIP) [file pone.0321058.s002.zip › S1 Figures/Figure 16 (d).tif]

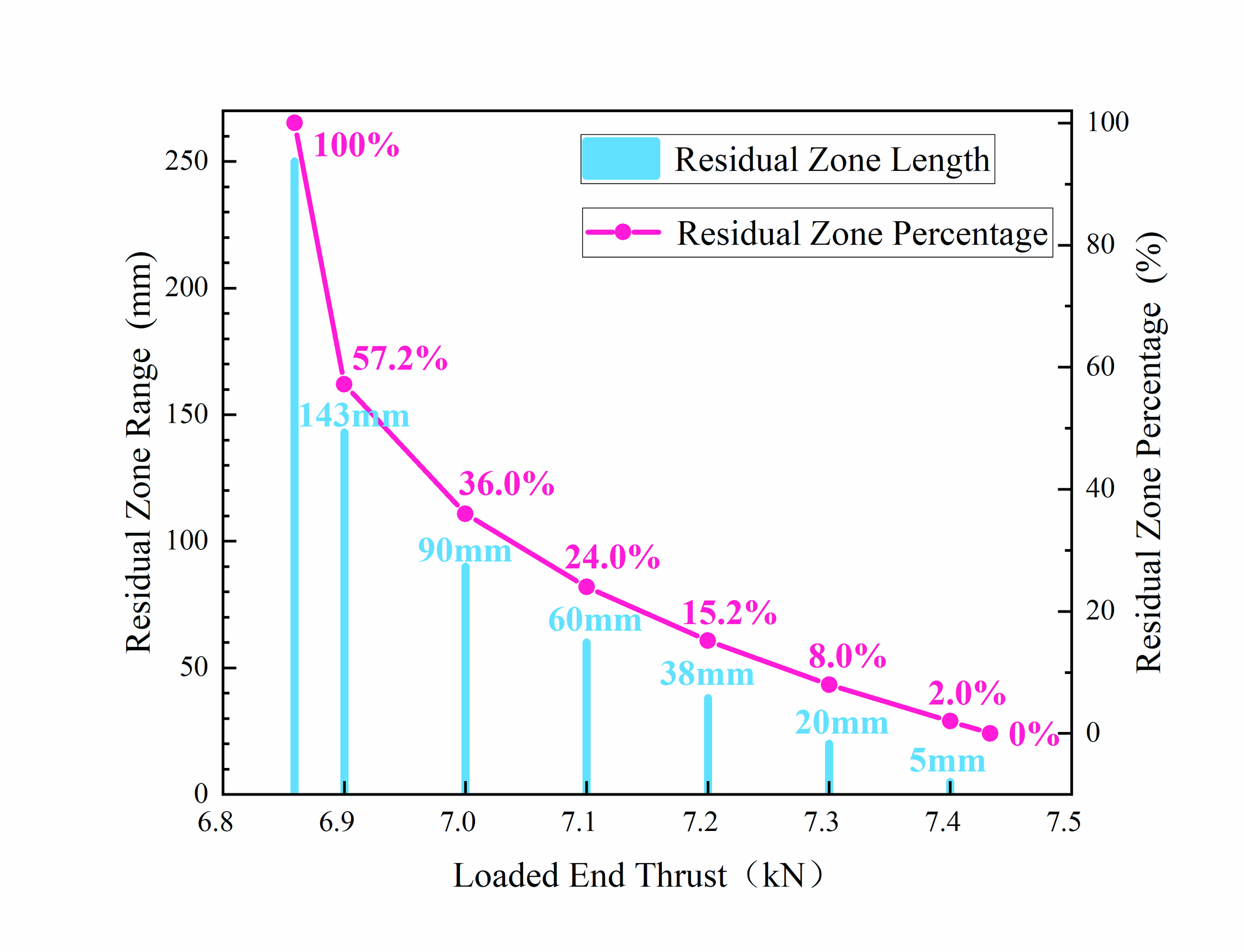

Supplement: S1 Fig — (ZIP) [file pone.0321058.s002.zip › S1 Figures/Figure 17 (a).tif]

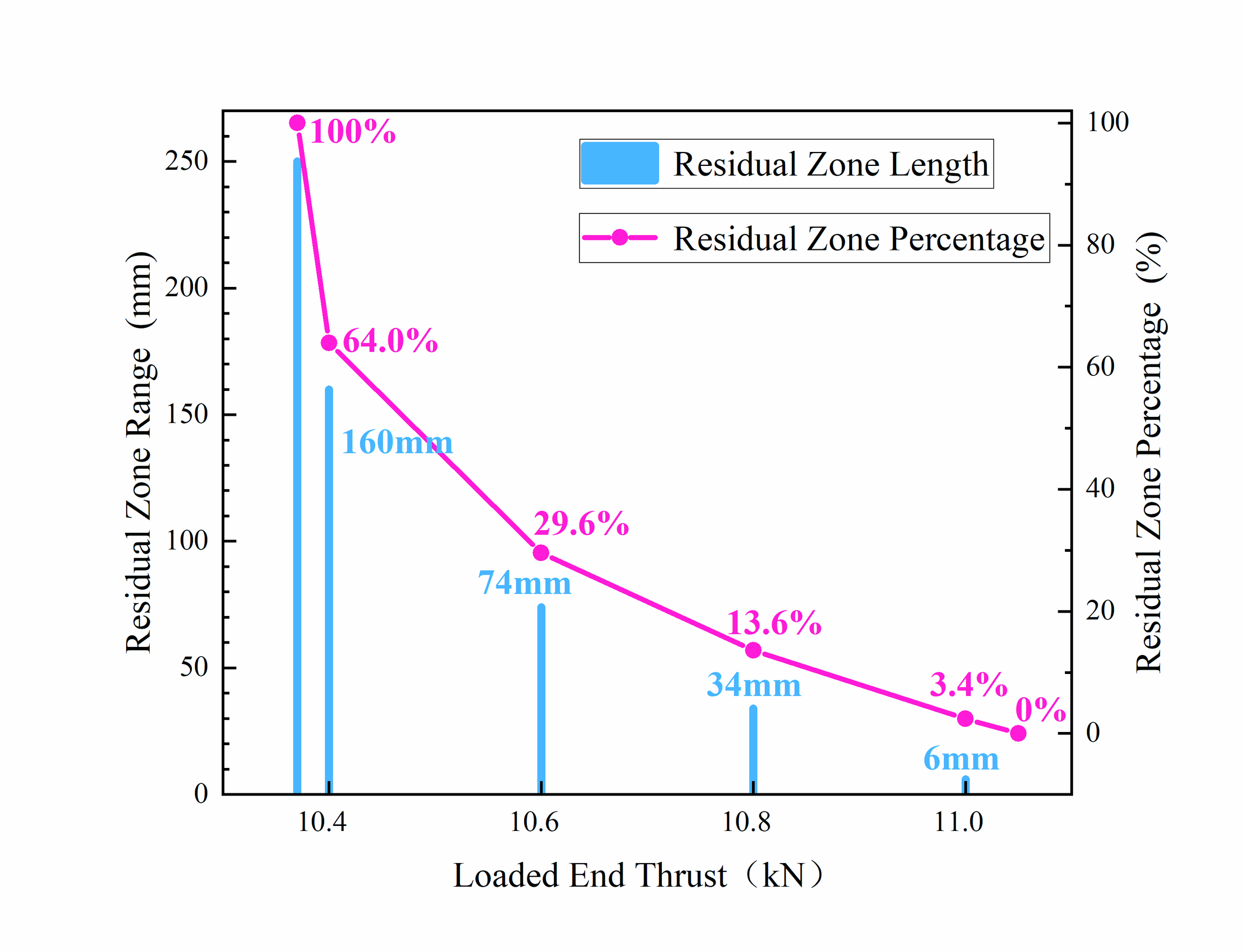

Supplement: S1 Fig — (ZIP) [file pone.0321058.s002.zip › S1 Figures/Figure 17 (b).tif]

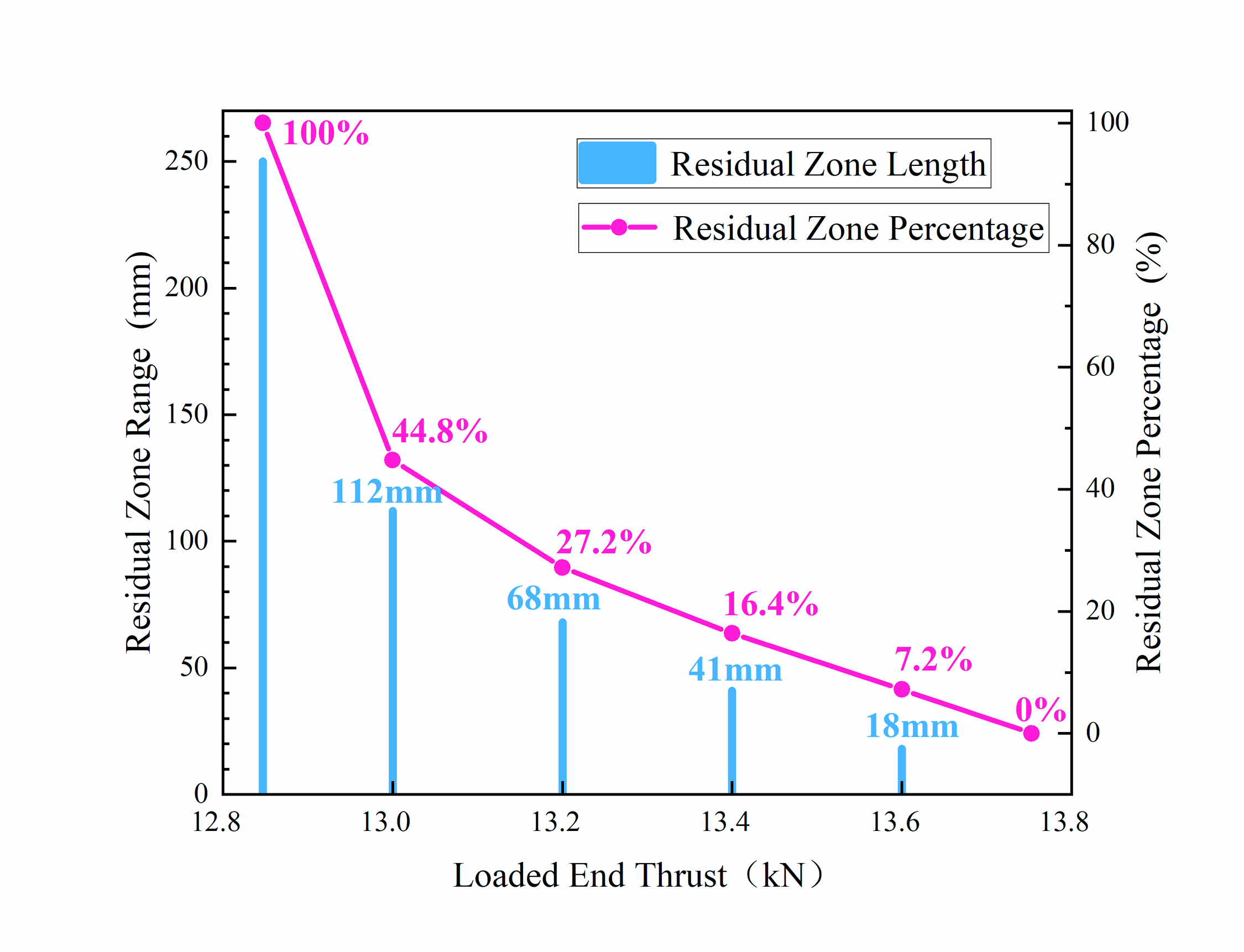

Supplement: S1 Fig — (ZIP) [file pone.0321058.s002.zip › S1 Figures/Figure 17 (c).tif]

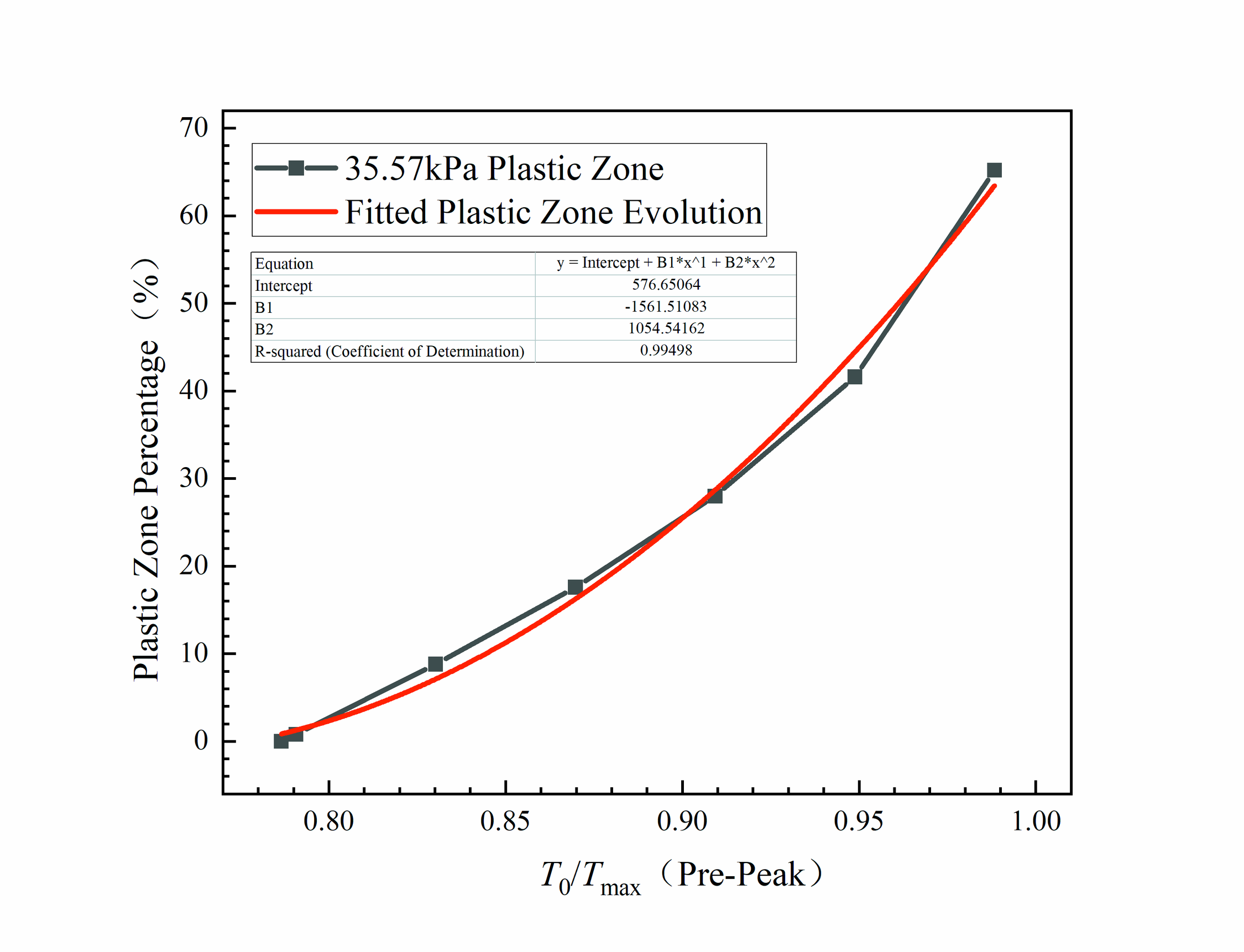

Supplement: S1 Fig — (ZIP) [file pone.0321058.s002.zip › S1 Figures/Figure 18 (a).tif]

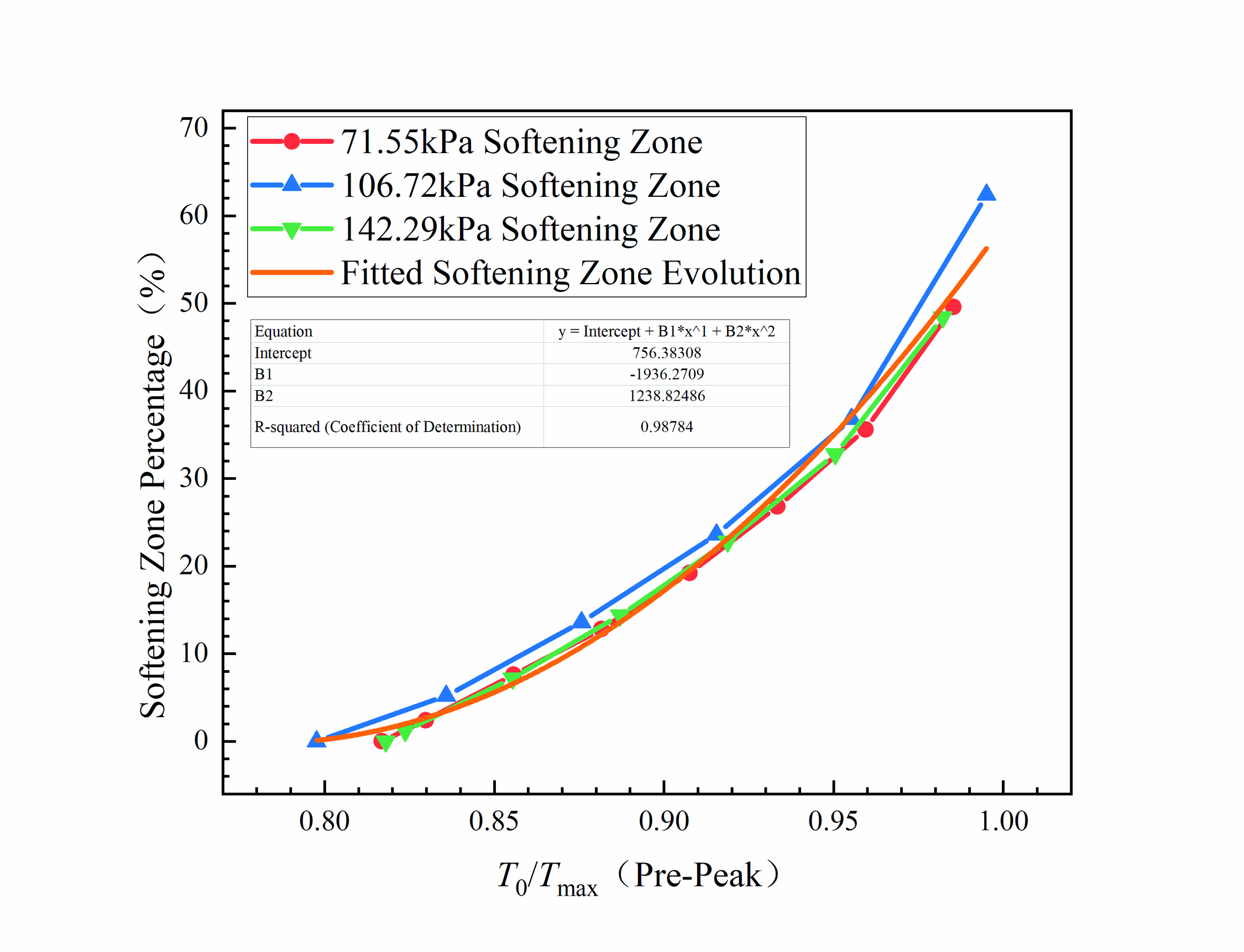

Supplement: S1 Fig — (ZIP) [file pone.0321058.s002.zip › S1 Figures/Figure 18 (b).tif]

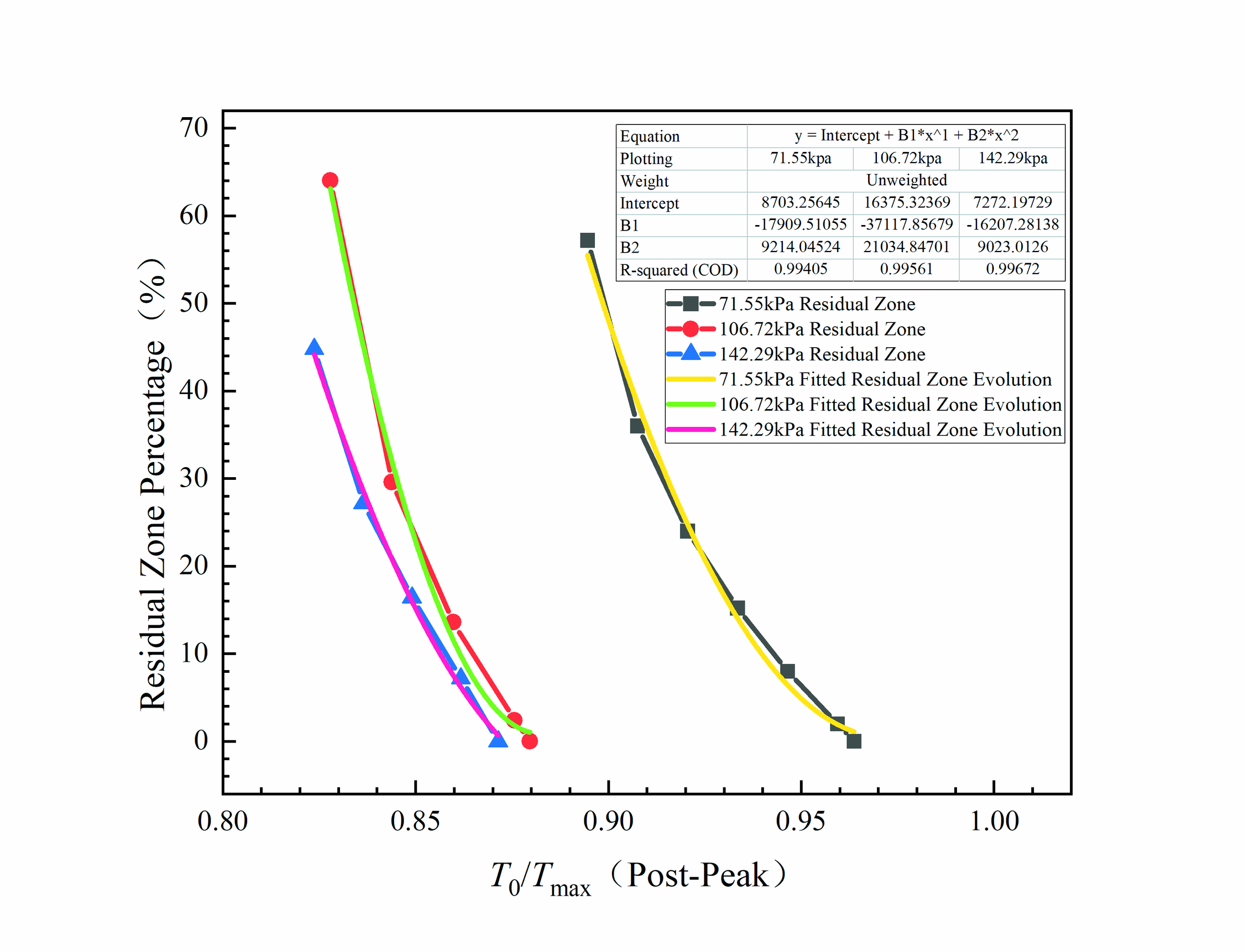

Supplement: S1 Fig — (ZIP) [file pone.0321058.s002.zip › S1 Figures/Figure 18 (c).tif]

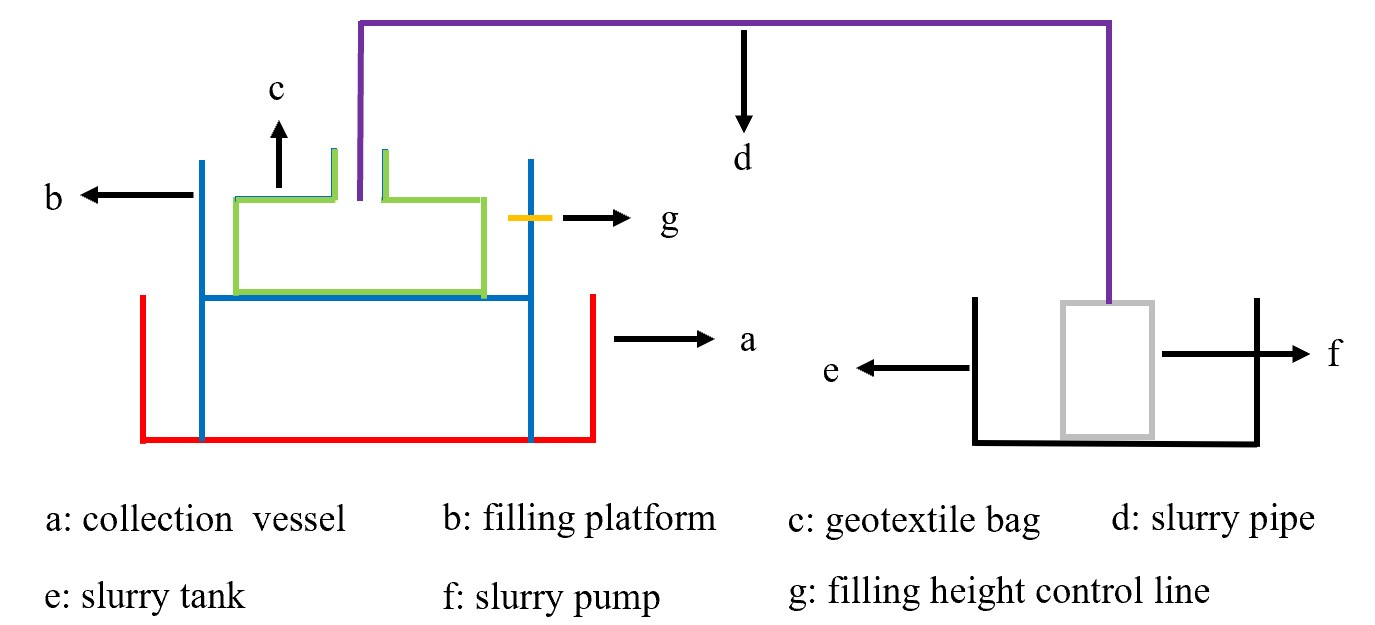

Supplement: S1 Fig — (ZIP) [file pone.0321058.s002.zip › S1 Figures/Figure 2.tif]

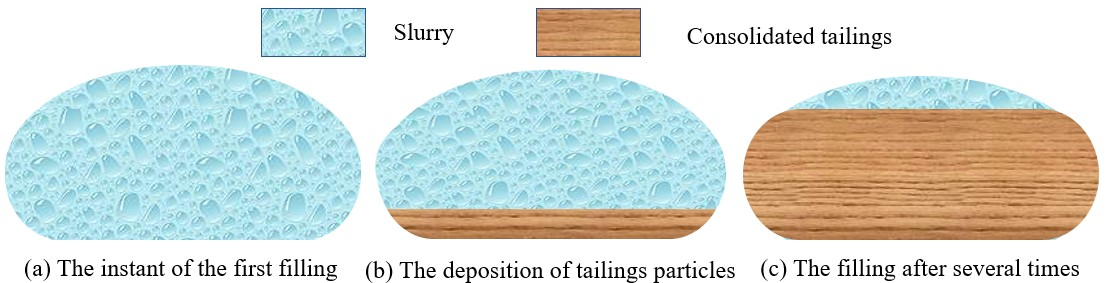

Supplement: S1 Fig — (ZIP) [file pone.0321058.s002.zip › S1 Figures/Figure 3.tif]

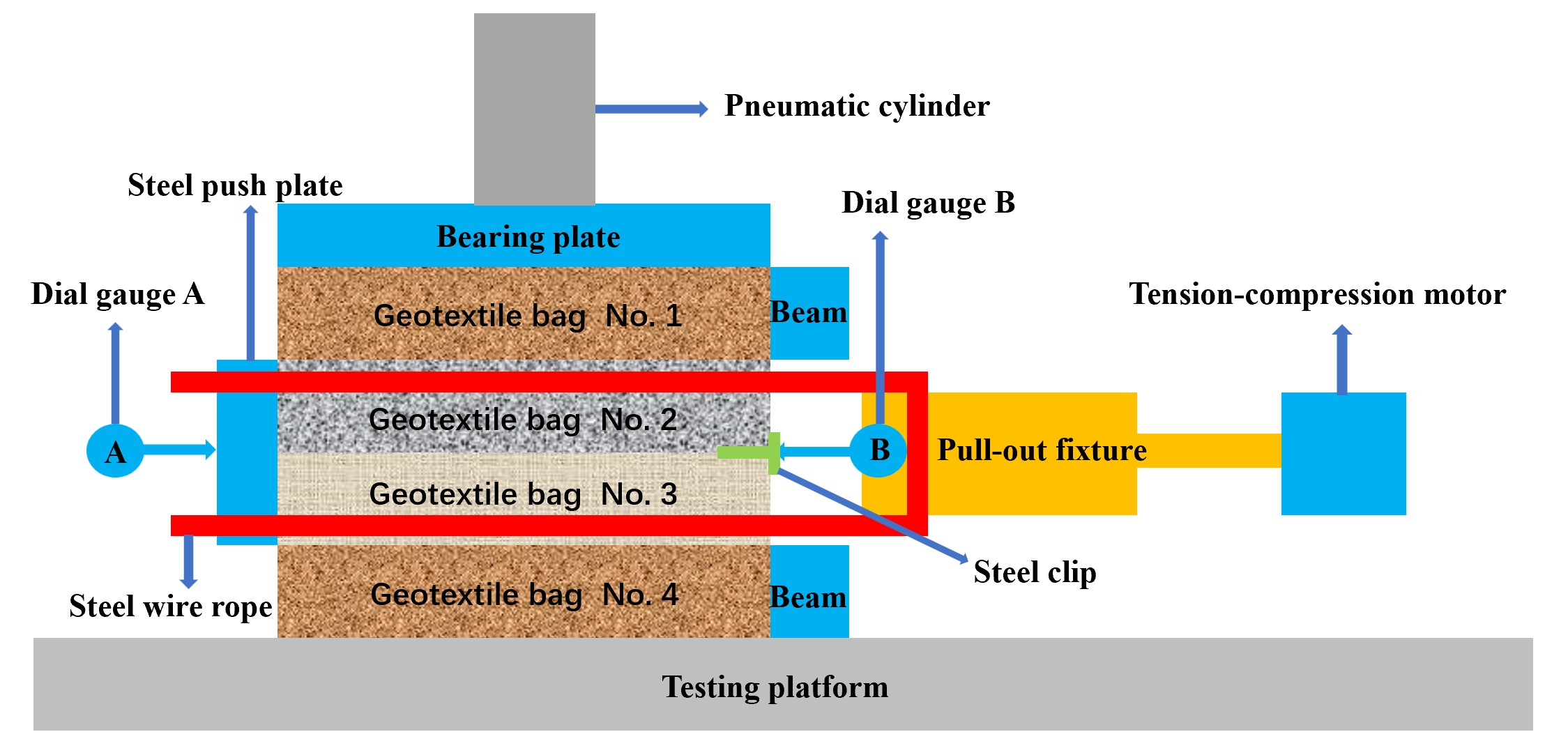

Supplement: S1 Fig — (ZIP) [file pone.0321058.s002.zip › S1 Figures/Figure 4.tif]

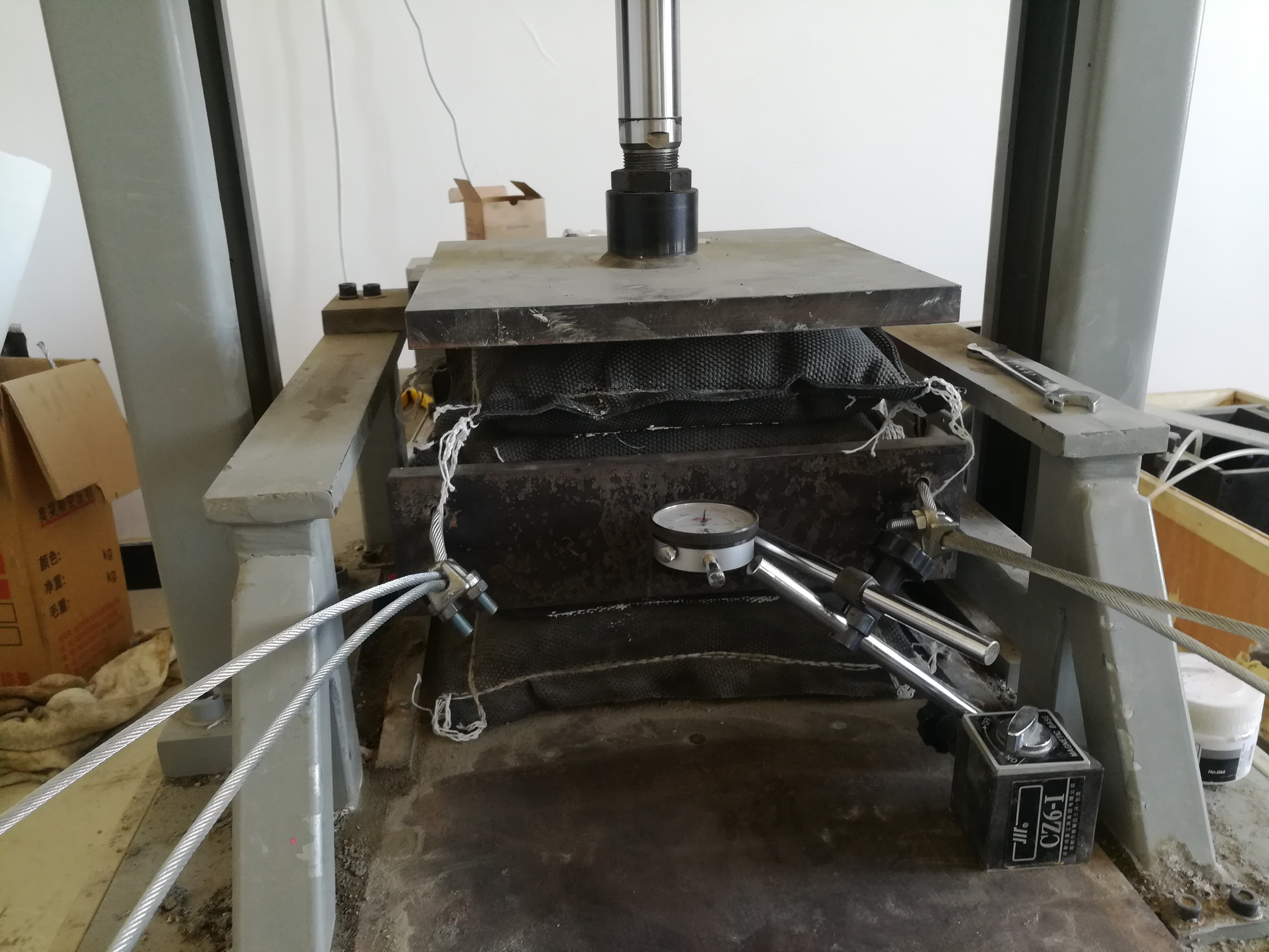

Supplement: S1 Fig — (ZIP) [file pone.0321058.s002.zip › S1 Figures/Figure 5(a).tif]

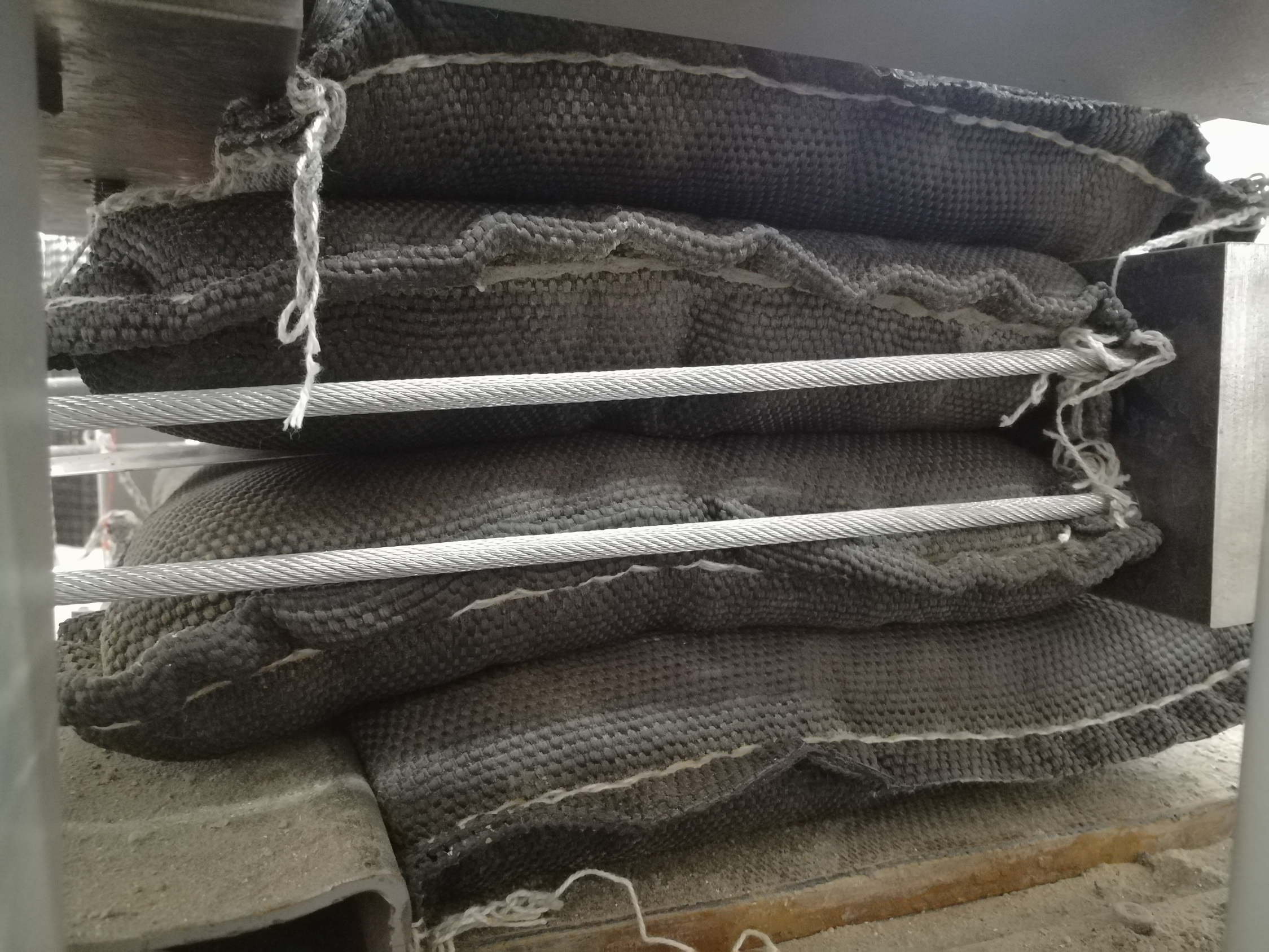

Supplement: S1 Fig — (ZIP) [file pone.0321058.s002.zip › S1 Figures/Figure 5(b).tif]

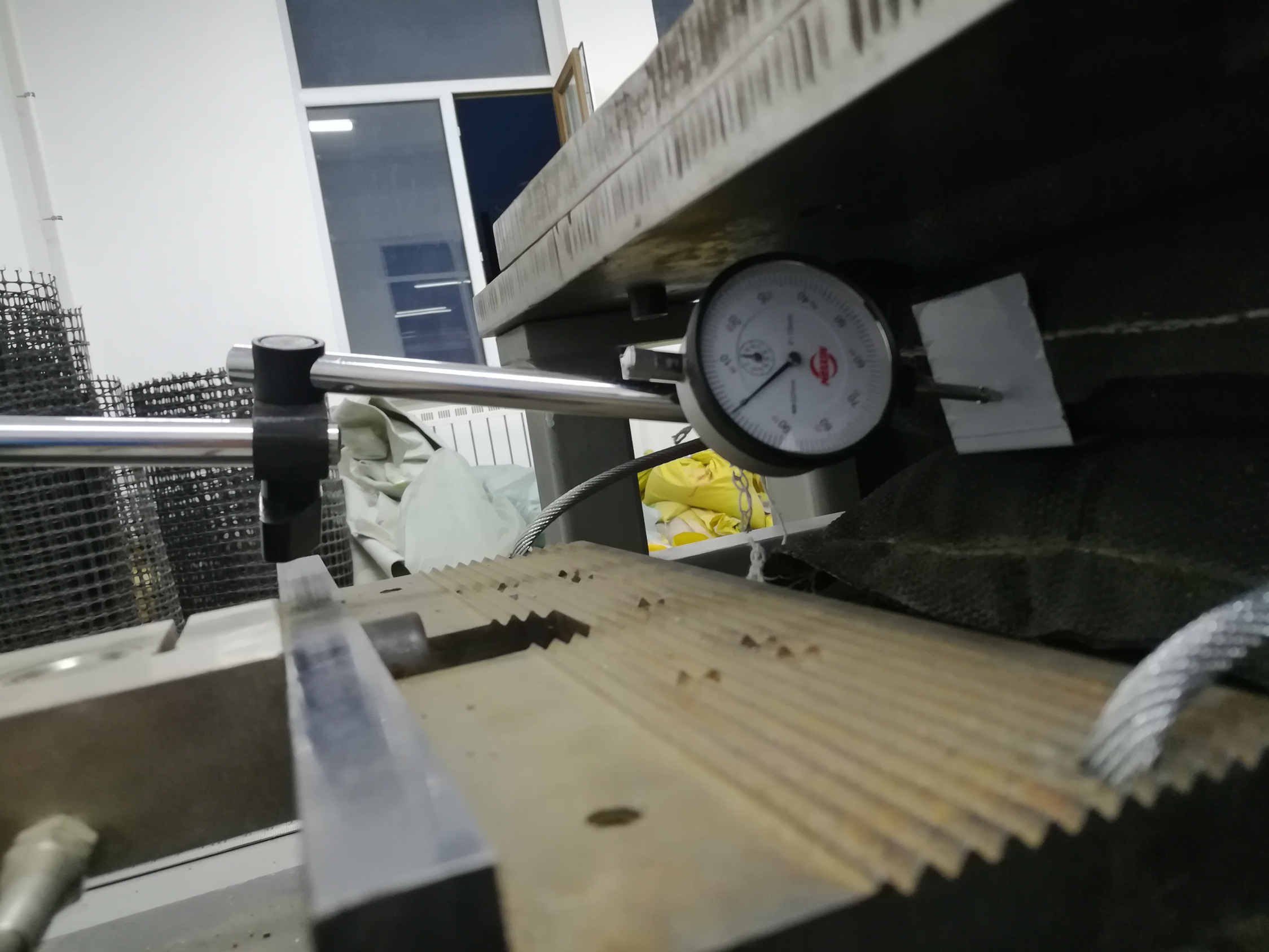

Supplement: S1 Fig — (ZIP) [file pone.0321058.s002.zip › S1 Figures/Figure 5(c).tif]

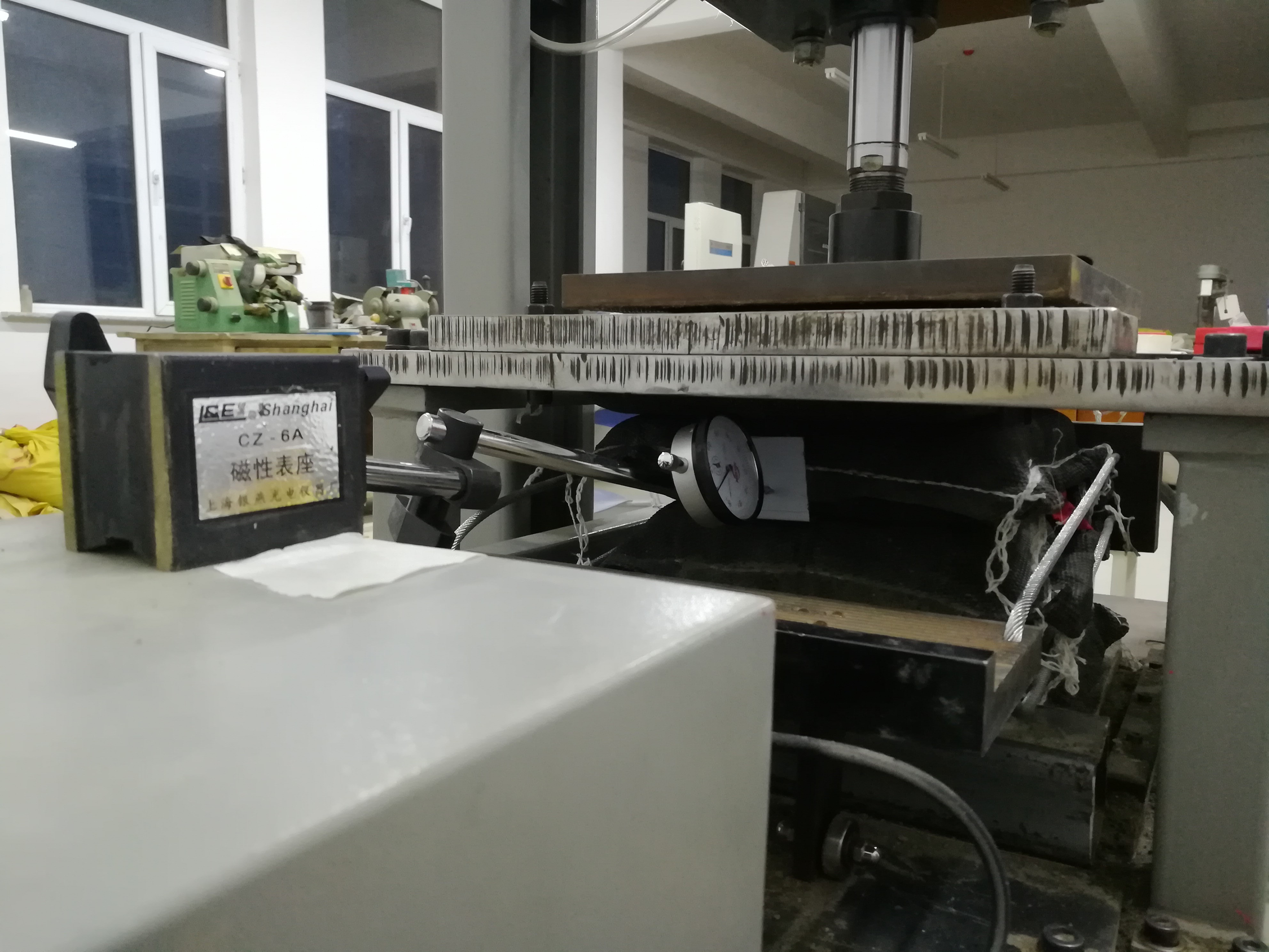

Supplement: S1 Fig — (ZIP) [file pone.0321058.s002.zip › S1 Figures/Figure 5(d).tif]

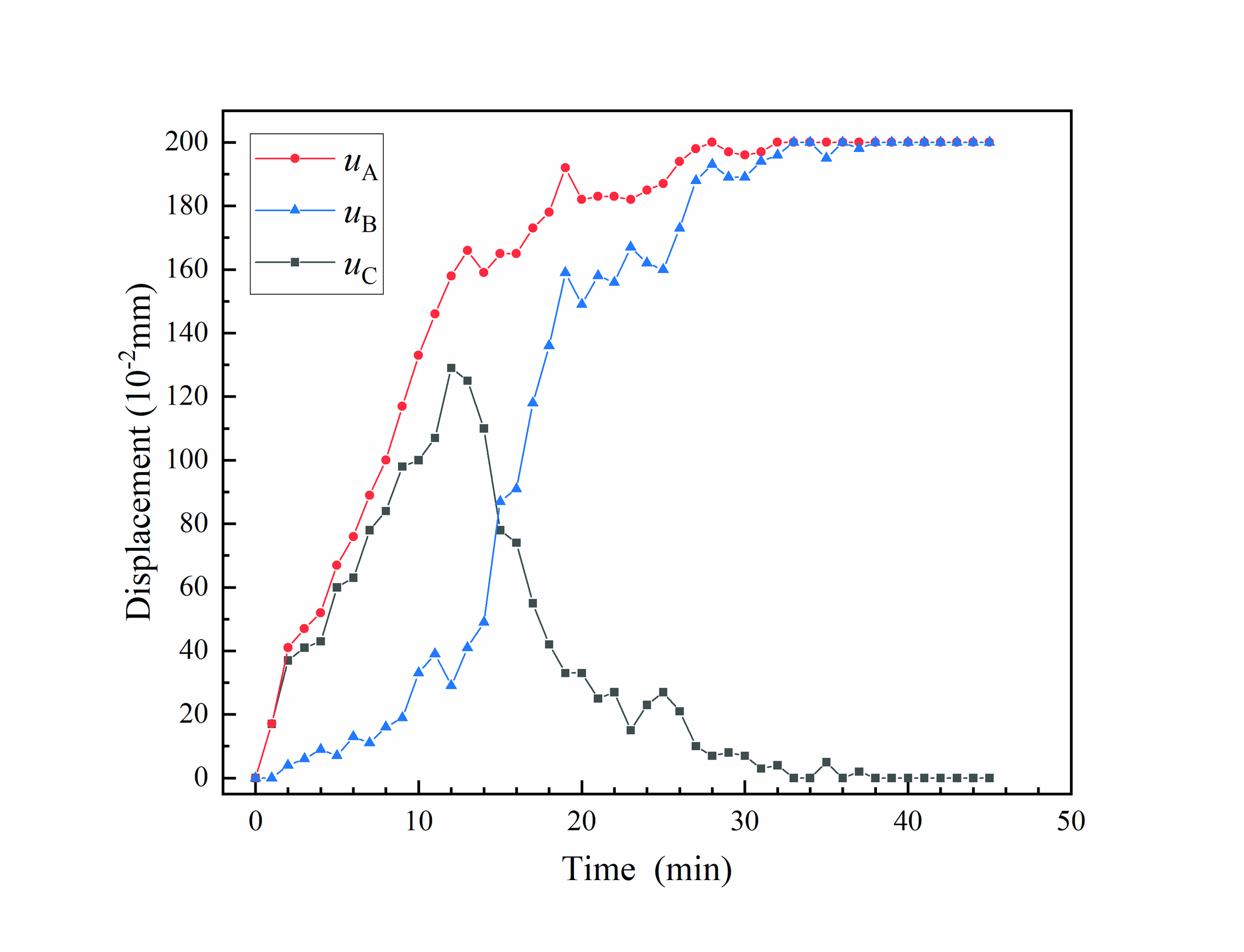

Supplement: S1 Fig — (ZIP) [file pone.0321058.s002.zip › S1 Figures/Figure 6. (b).tif]

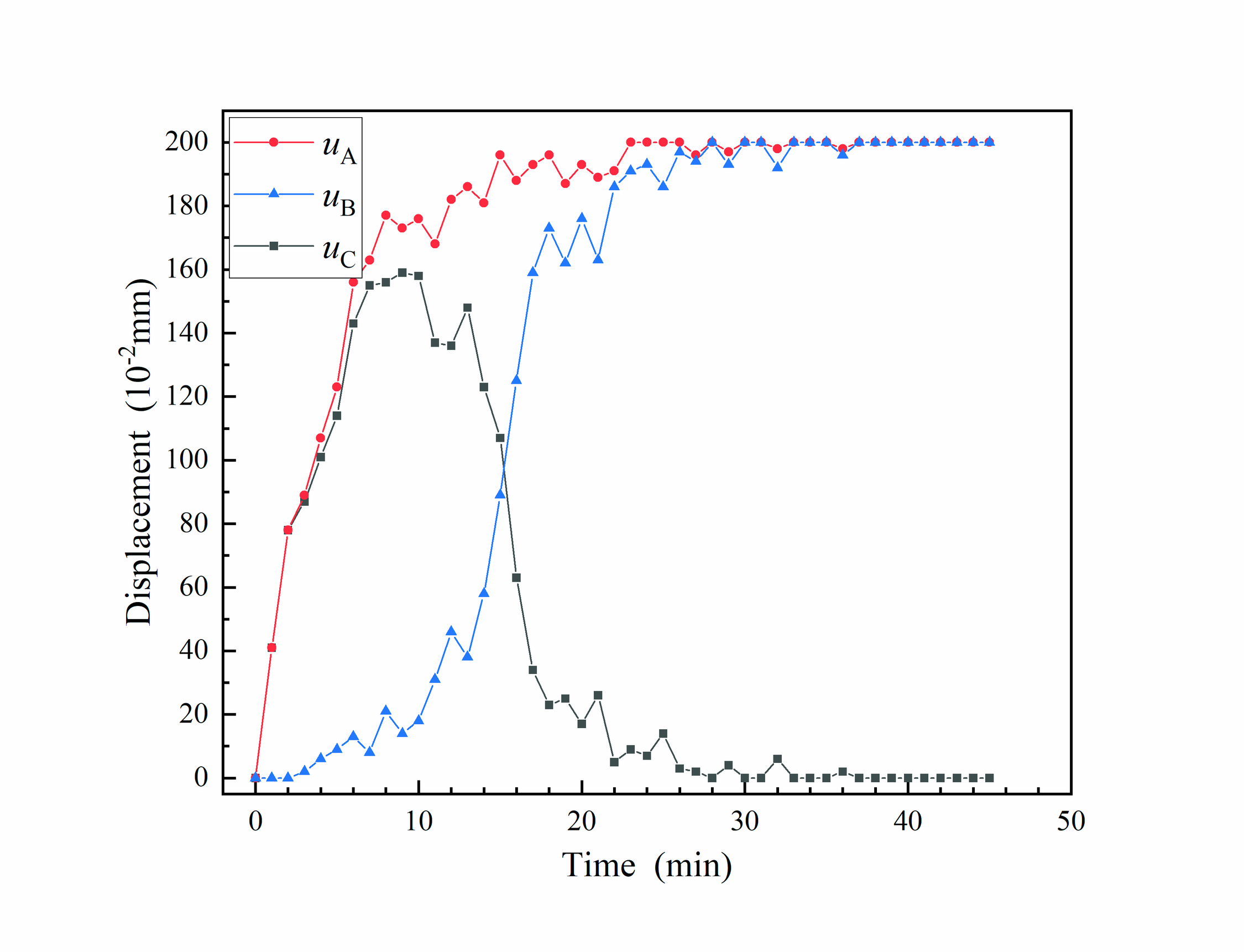

Supplement: S1 Fig — (ZIP) [file pone.0321058.s002.zip › S1 Figures/Figure 6. (c).tif]

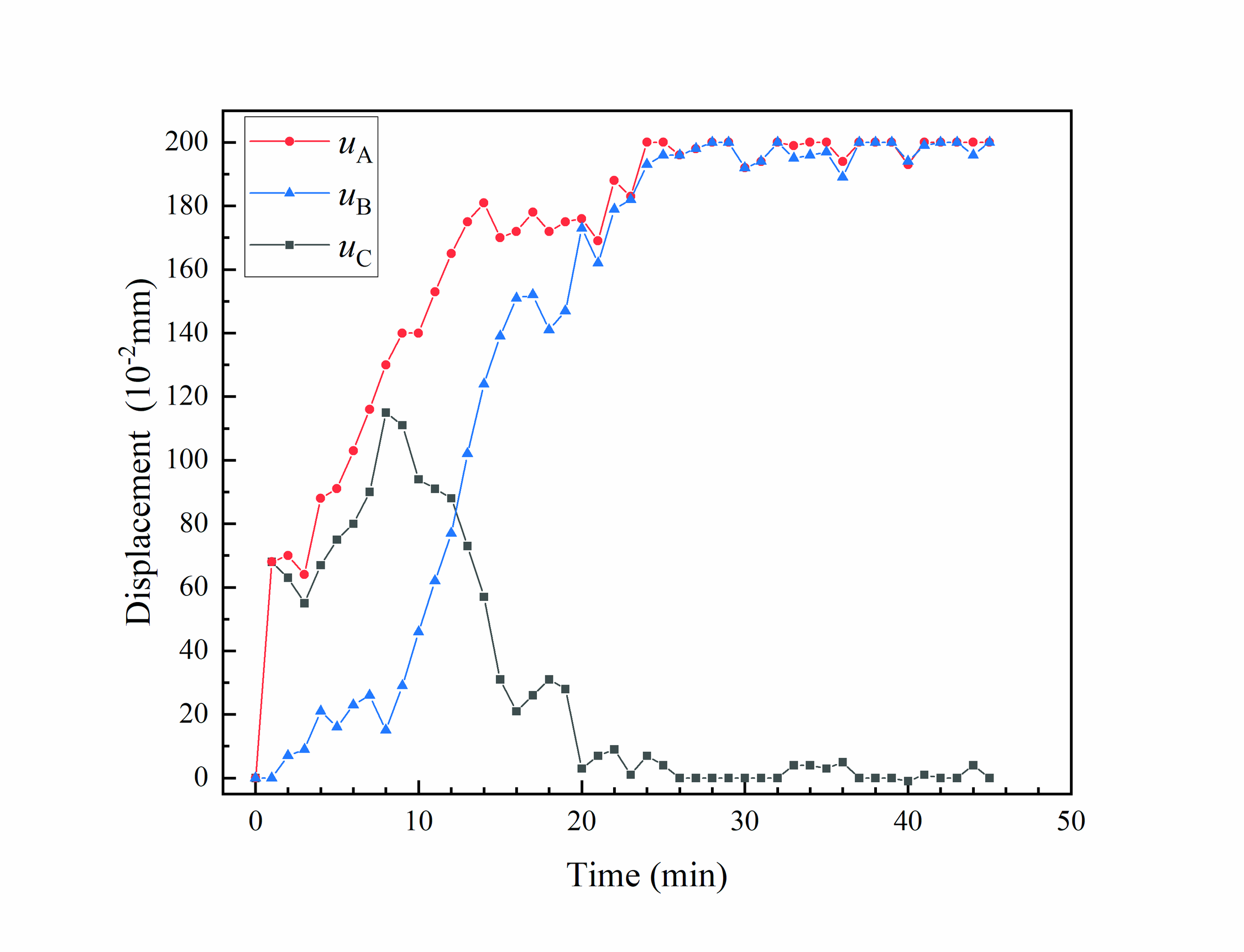

Supplement: S1 Fig — (ZIP) [file pone.0321058.s002.zip › S1 Figures/Figure 6. (d).tif]

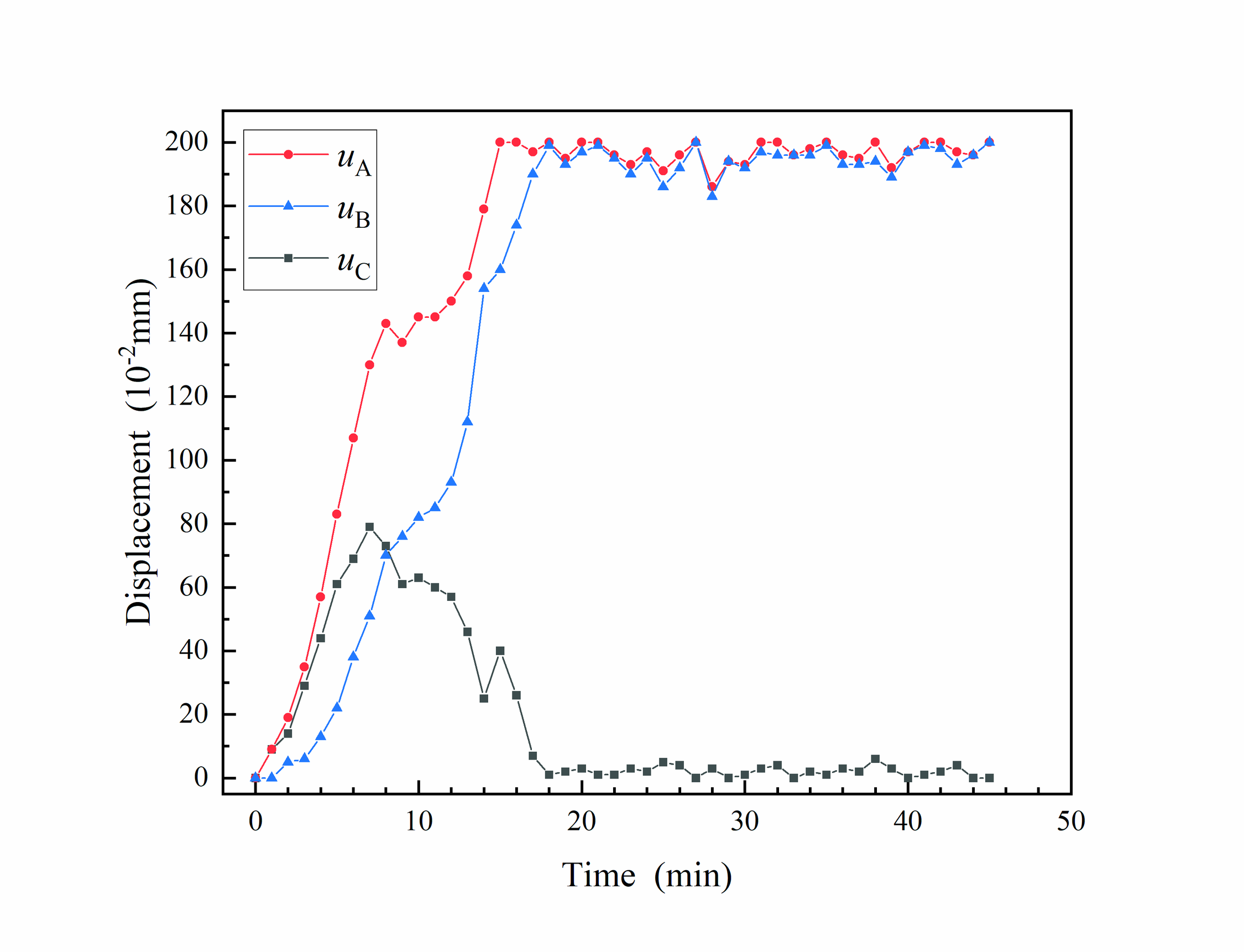

Supplement: S1 Fig — (ZIP) [file pone.0321058.s002.zip › S1 Figures/Figure 6.(a).tif]

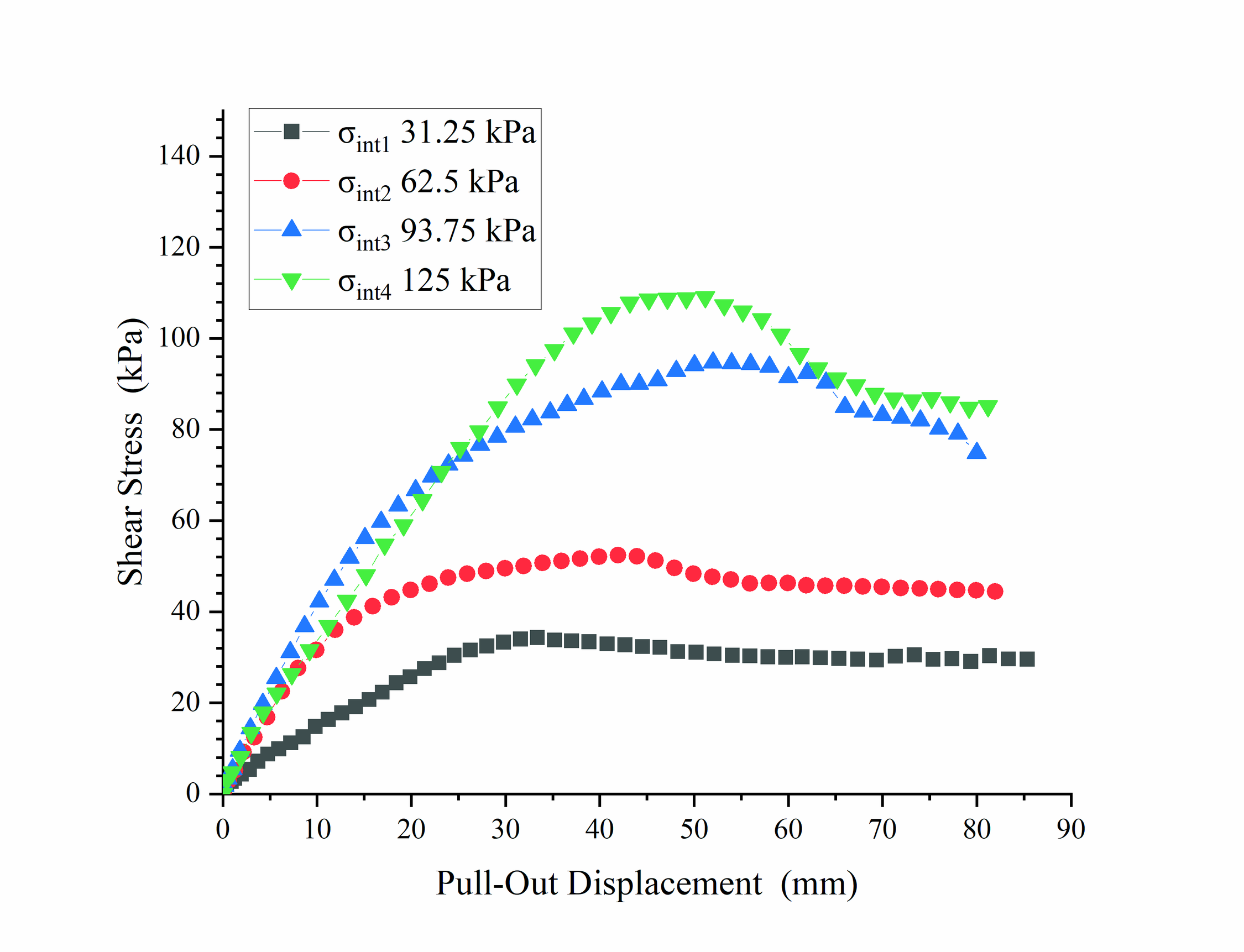

Supplement: S1 Fig — (ZIP) [file pone.0321058.s002.zip › S1 Figures/Figure 7.(a).tif]

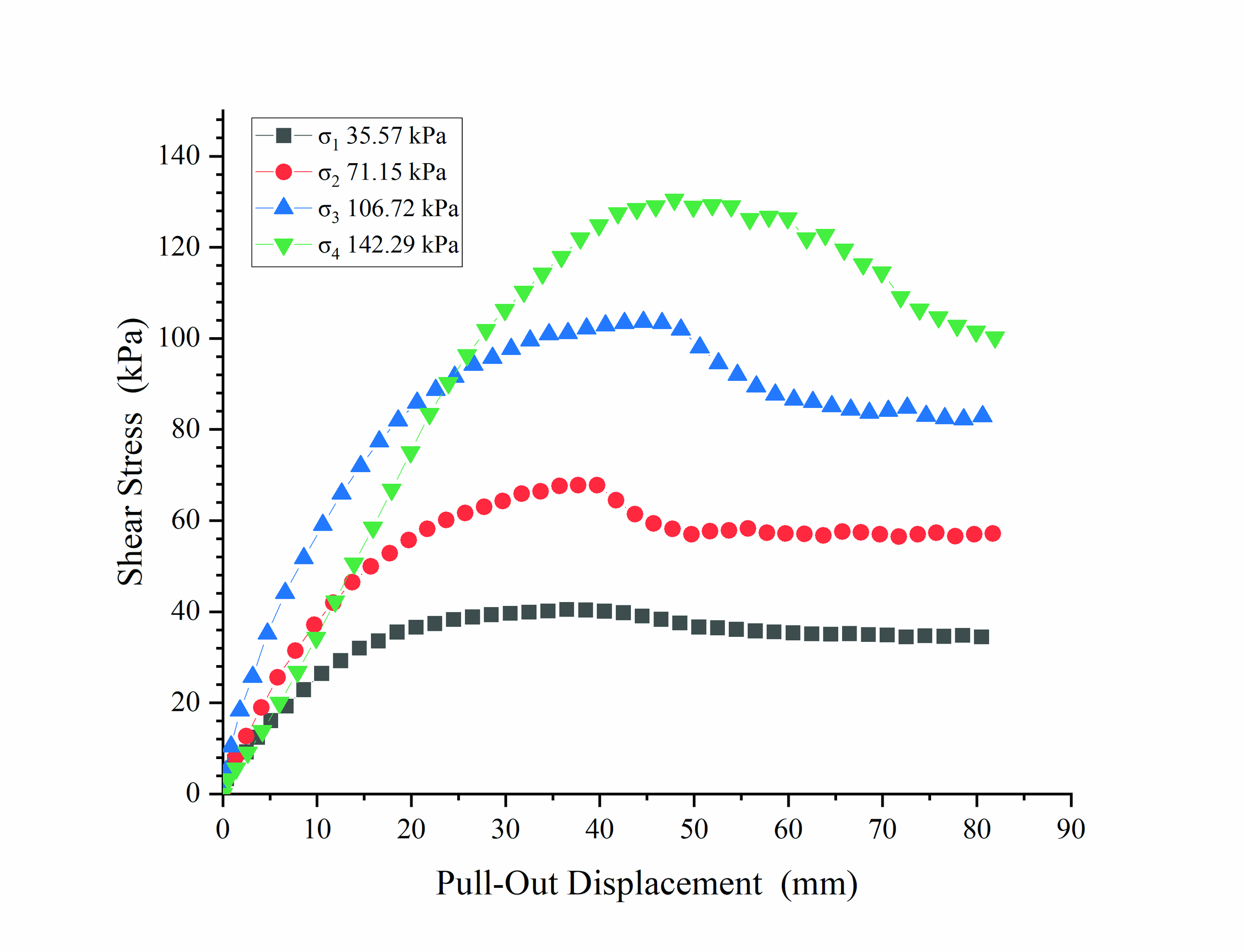

Supplement: S1 Fig — (ZIP) [file pone.0321058.s002.zip › S1 Figures/Figure 7.(b).tif]

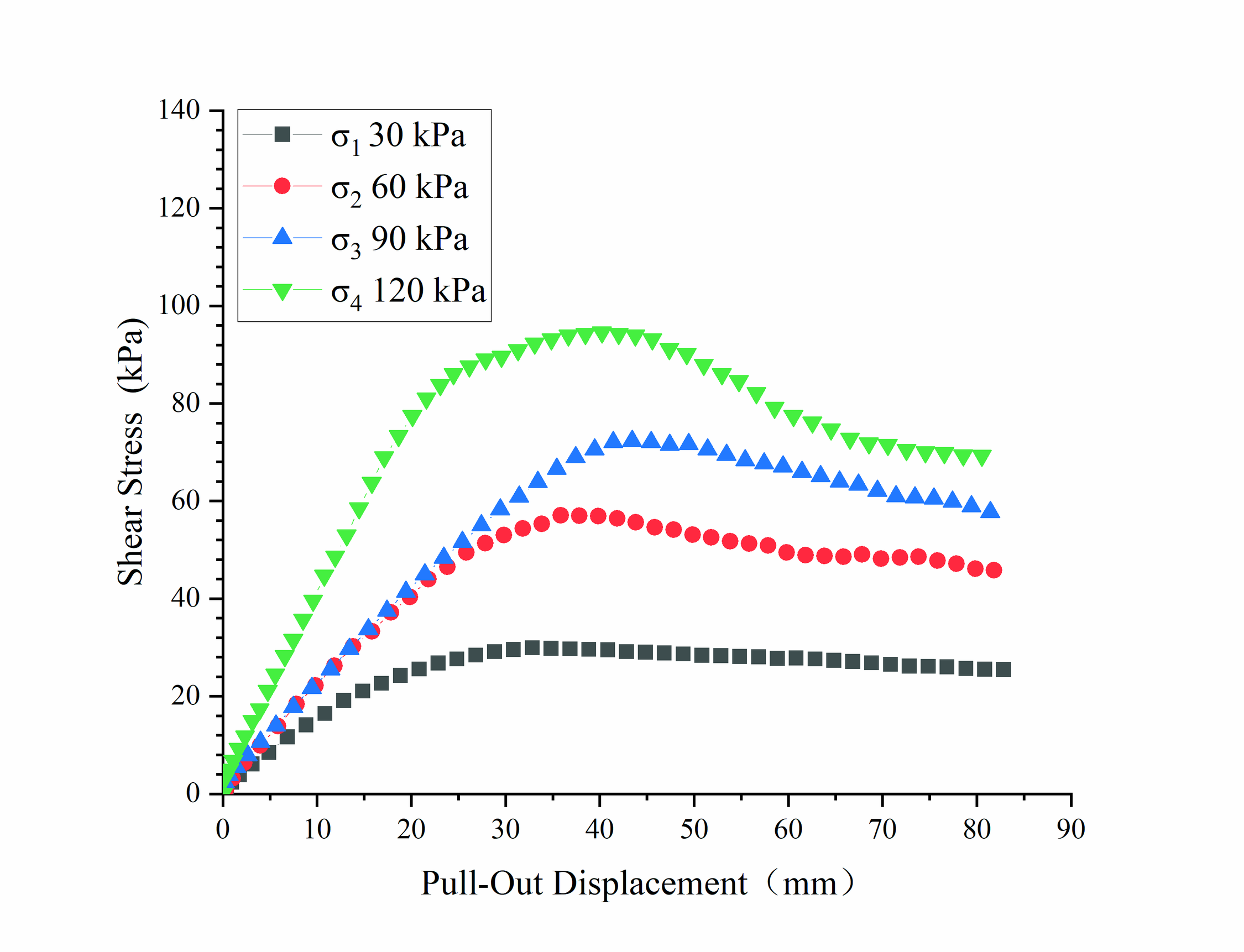

Supplement: S1 Fig — (ZIP) [file pone.0321058.s002.zip › S1 Figures/Figure 7.(c).tif]

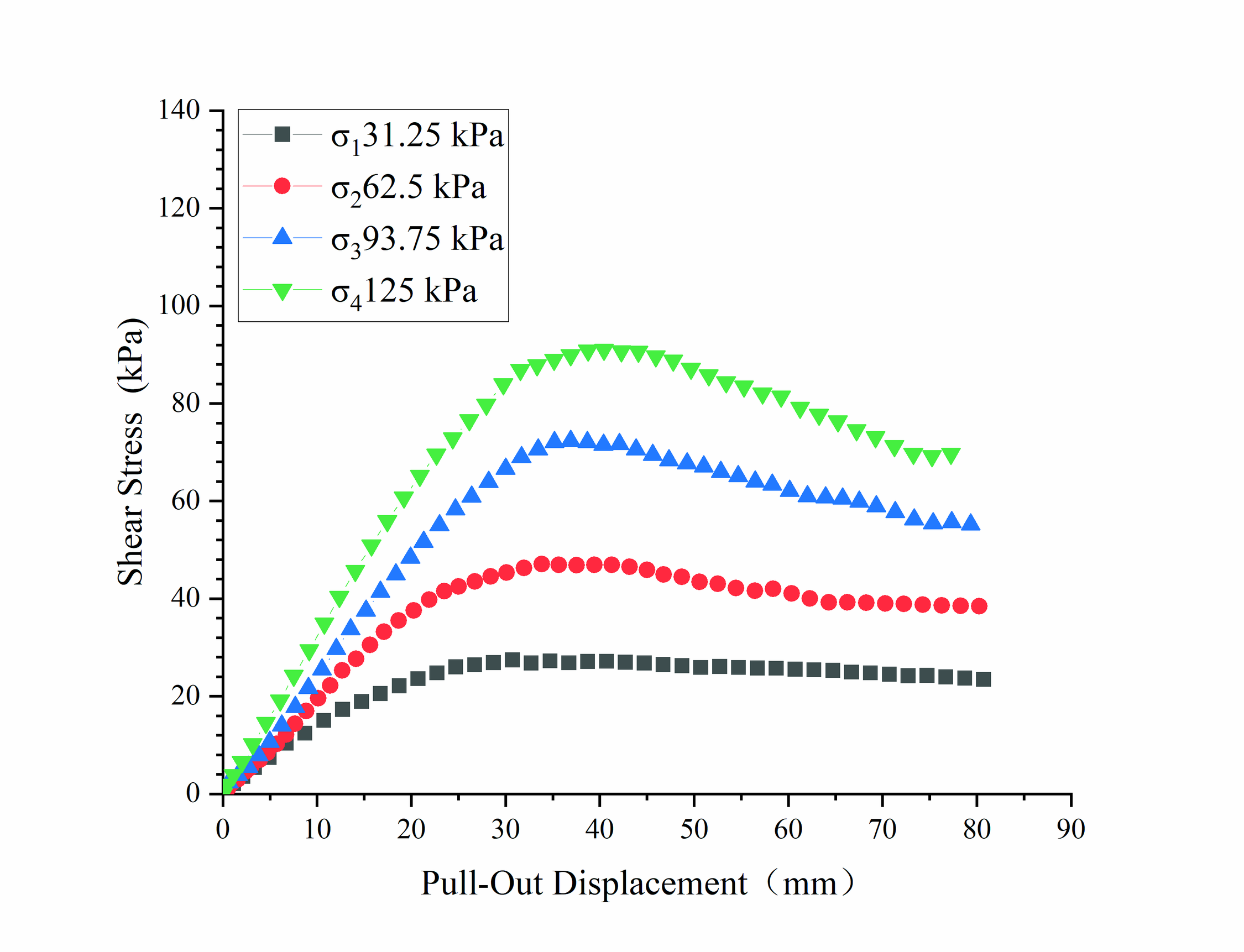

Supplement: S1 Fig — (ZIP) [file pone.0321058.s002.zip › S1 Figures/Figure 7.(d).tif]

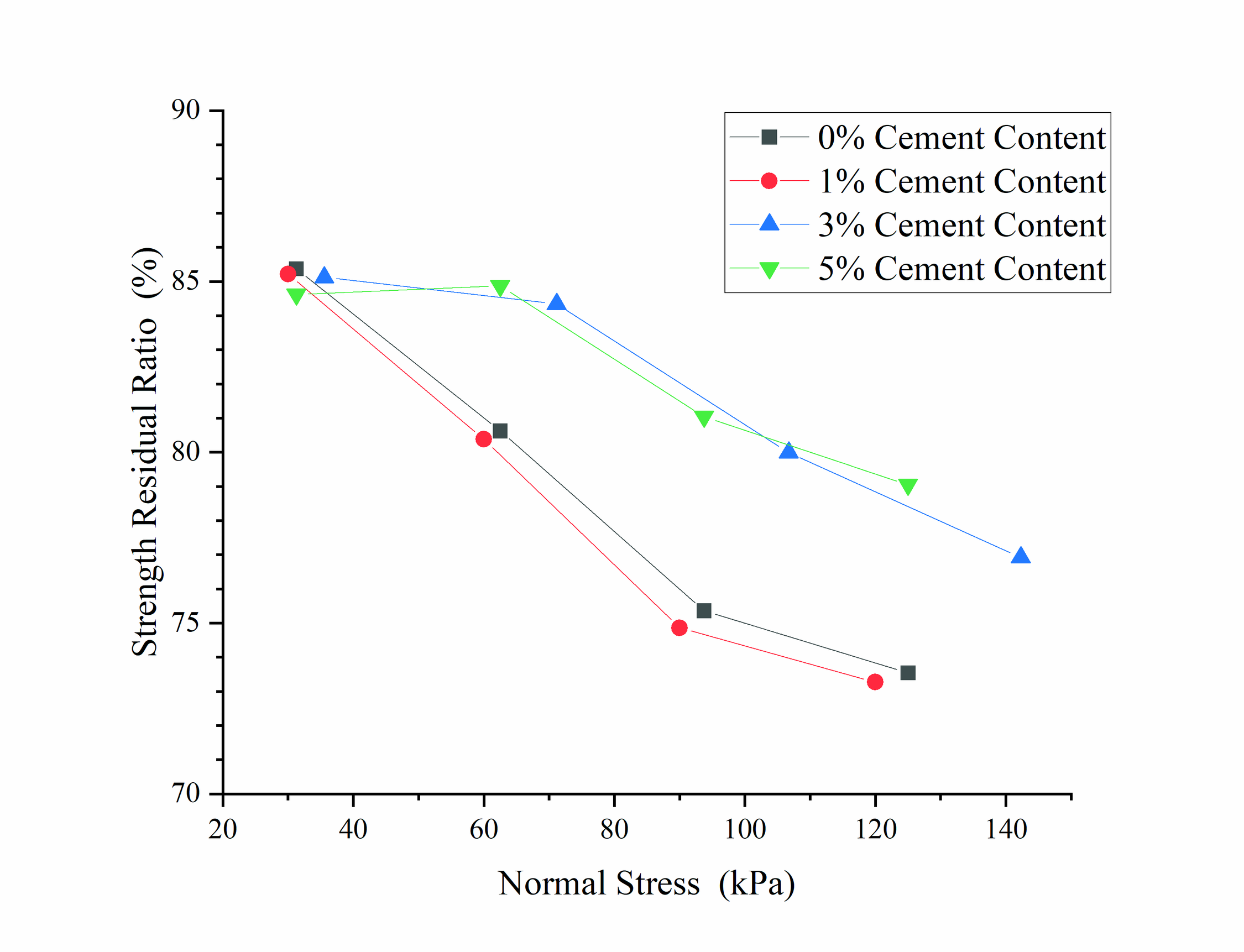

Supplement: S1 Fig — (ZIP) [file pone.0321058.s002.zip › S1 Figures/Figure 8.tif]

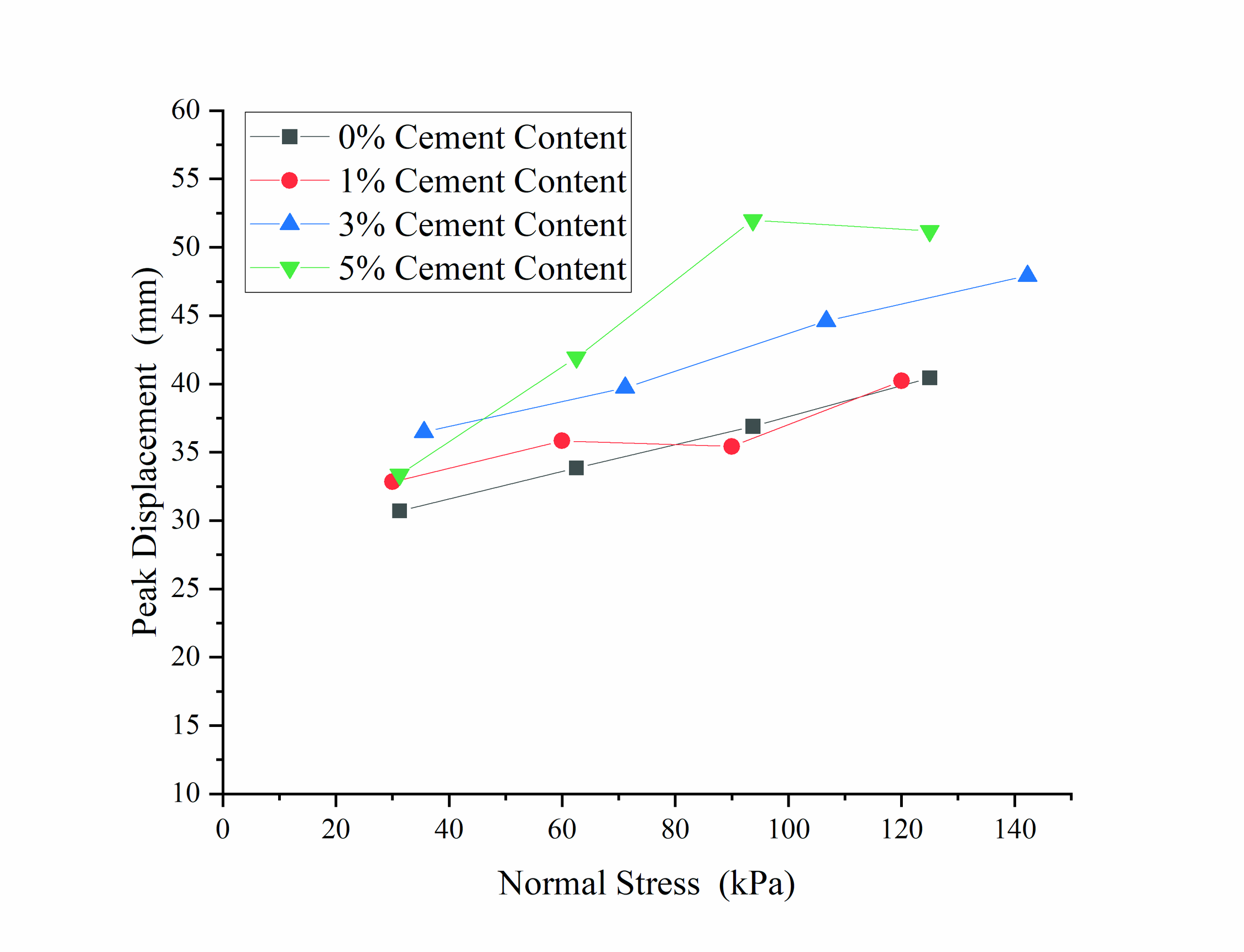

Supplement: S1 Fig — (ZIP) [file pone.0321058.s002.zip › S1 Figures/Figure 9.tif]
